# Energy‐Saving Pathways for Thermoelectric Nanomaterial Synthesis: Hydrothermal/Solvothermal, Microwave‐Assisted, Solution‐Based, and Powder Processing

**DOI:** 10.1002/advs.202106052

**Published:** 2022-07-17

**Authors:** Nagaraj Nandihalli, Duncan H. Gregory, Takao Mori

**Affiliations:** ^1^ National Institute for Materials Science (NIMS) International Center for Materials Nanoarchitectonics (WPI‐MANA) Namiki 1‐1 Tsukuba 305‐0044 Japan; ^2^ WestCHEM School of Chemistry University of Glasgow Glasgow G82 5EZ UK

**Keywords:** green synthesis, hydrothermal/solvothermal synthesis, mechanical alloying, microwave assisted synthesis, solution‐based synthesis, thermoelectric nanoparticles

## Abstract

The pillars of Green Chemistry necessitate the development of new chemical methodologies and processes that can benefit chemical synthesis in terms of energy efficiency, conservation of resources, product selectivity, operational simplicity and, crucially, health, safety, and environmental impact. Implementation of green principles whenever possible can spur the growth of benign scientific technologies by considering environmental, economical, and societal sustainability in parallel. These principles seem especially important in the context of the manufacture of materials for sustainable energy and environmental applications. In this review, the production of energy conversion materials is taken as an exemplar, by examining the recent growth in the energy‐efficient synthesis of thermoelectric nanomaterials for use in devices for thermal energy harvesting. Specifically, “soft chemistry” techniques such as solution‐based, solvothermal, microwave‐assisted, and mechanochemical (ball‐milling) methods as viable and sustainable alternatives to processes performed at high temperature and/or pressure are focused. How some of these new approaches are also considered to thermoelectric materials fabrication can influence the properties and performance of the nanomaterials so‐produced and the prospects of developing such techniques further.

## Graphical abstract

Soft chemistry techniques such as hydrothermal/solvothermal, microwave‐assisted, solution‐based as well as powder‐based techniques are energy‐saving synthesis techniques, which can be utilized for thermoelectric materials for example. Understanding the underlying mechanism/factors behind such techniques is crucial for high product yield, high product purity, and high reproducibility in short time while spending less energy, and this review examines these aspects in detail.

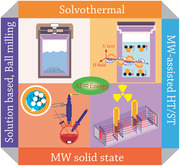

## 1 Introduction

Thermoelectric (TE) materials are energy harnessing solid‐state semiconductors that transform thermal energy into electricity or create a temperature difference from an applied voltage.^[^ ^1^ ^]^ The potential applications of TE materials are numerous.^[^ ^2^ ^]^ Among many exciting and prominent emerging applications are wearable TE devices that can convert body heat into electrical energy. Such devices could drive low power consumption implants such as deep‐brain stimulators, artificial cochlea, health parameter sensors, and various similar components within the internet‐of‐things (IoT) to monitor patient health remotely.^[^ ^3^ ^]^ Even in contemporary society, many state‐of‐the‐art devices require little input power so that sensors such as electronic tracking tags, which are able to operate at 0.1 mW, are ideal candidates to be driven by TE devices.^[^ ^4^ ^]^ Other potential applications of TEs lie at completely the opposite scale; TE generator systems that can harvest industrial and automotive waste heat to produce electrical energy are likely to be major contributors to sustainable power generation and could play a major role in the reduction of carbon emissions over the years to come.^[^ ^5^ ^]^ TE devices have a number of salient features that characterize their attractiveness as a superior energy conversion technology: high reliability, easy miniaturization, geometrically accommodative, an absence of moving parts, noise‐free, low maintenance, extended lifetimes, high‐precision temperature control, and an ability to function in extreme environments (which can be ideal for both power generation and remote sensing).

### 1.1 Governing Parameters in Thermoelectrics and the Role of Nanostructuring

**Figure 1:**
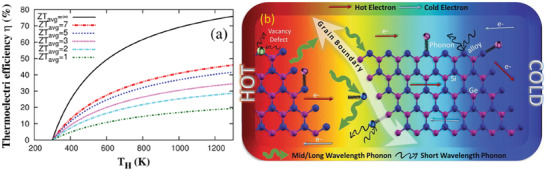
a) TE efficiency as a function of the hot side temperature (*T*_H_) with a fixed cold‐side temperature, *T*_C_ = 300 K for different *zT*_avg_ values. Adapted with permission.^[^ ^10^ ^]^ Copyright 2019, Springer Nature. b) Scheme of the most often used strategies for reducing *κ* and their effect on phonon scattering in SiGe alloy. Grain boundaries scatter mid‐long wavelength phonons, while alloy atoms, dopants, defects, lattice vibrations, and nanoinclusions scatter short‐wavelength phonons. The electrons, represented as arrows, are supposedly not scattered and so *σ* is unaffected. Adapted with permission.^[^ ^11^ ^]^ Copyright 2017, In Tech.

The parameter that enumerates the performance of a thermoelectric (TE) material is its figure‐of‐merit (*zT*) and is given by

(1) $$\mathrm{zT}=\frac{S^{2}\sigma T}{\kappa }$$

where *S* is the Seebeck coefficient (also called thermopower), *σ *is electrical conductivity, *κ *is thermal conductivity, and *T* is temperature. From the above relation, it is clear that for a high *zT*, a TE material requires a high *S*, high *σ* and, low *κ*.^[^ ^1^ ^]^ When a temperature differential (Δ*T*) between the hot and cold ends of a thermoelement is formed, charge carriers on the hot side begin to diffuse to the cold side, accumulating a charge and potential difference (Δ*V*). The parameter ‐Δ*V*/Δ*T* is the Seebeck coefficient. The thermal conductivity, *κ*, generally consists of two parts: the lattice contribution, *κ*_l_ and the electronic contribution, *κ*_e (_i.e., *κ* = *κ*_l +_ *κ*_e)._The *σ *and *κ_e_* are directly related according to Wiedemann–Franz law, *κ*_e =_ *L*_o_ *σΤ*. Where *T* is the temperature and *L*_o_ is the Lorenz number (2.45 × 10^−8^ V^2^ K^−2^ for metals and degenerate semiconductors). The parameters *σ* and *S* are reciprocally connected. Decoupling these parameters is a formidable challenge.^[^ ^6^ ^]^ The power factor is defined as *PF* = *S* ^2^ *σ*, which provides a means to assess the combined effect of *σ* and *S* on TE properties. The relationship between *zT* and the maximum theoretical efficiency for a TE material is expressed by

(2) $$\eta_{\max}=\frac{\Delta T}{T}\frac{\sqrt{1+{\mathrm{zT}}_{\mathrm{avg}}}-1}{\sqrt{1+{\mathrm{zT}}_{\mathrm{avg}}}+\frac{T_{c}}{T_{H}}}$$

where *T*_C_ and *T*_H_ are the cold side and hot side temperatures of the material respectively, *zT*_avg_ is the arithmetic average of *zT*_H_ and *zT*_C_. The efficiency is plotted as a function of *T*_H_ assuming *T*_C_ = 300 K and for a series of *zT*_avg_ values (**Figure** **1**a). From the figure, it is clear that to increase the efficiency both high *zT* and a large temperature difference across the material are needed. An efficiency range of 20–30% is a threshold window to compete with traditional power generators, which are capable of reaching 40% of Carnot efficiency. This efficiency is needed to use TE materials in commercial applications.

The lattice thermal conductivity of a bulk TE material is given by

(3) $$\kappa_{l}=\left(\frac{1}{3}\right)C_{v}v_{g}l$$

where, *C*_V_ is the specific heat, *ν*_g_ is the phonon group velocity and *l* is the mean‐free path of phonons. From this relation, it can be readily understood that a low phonon group velocity *ν*_g_ can contribute to an intrinsically low value for *κ*_l_ in TE materials. One method to reduce *ν*_g_ is via nanostructuring, i.e., to create nanoparticles of the TE material of a suitable size and shape or to generate nanostructures within the bulk TE material itself. Nanostructuring approaches within bulk materials (such as introducing dopants and point defects) provide the means to scatter both mid‐ and long‐wavelength phonons, collectively reducing the phonon group velocity. Moreover, by following nanostructuring principles, one can significantly increase the number of interfaces (grain boundaries; extended defects) enabling additional grain boundary phonon scattering and further reduction of the *κ*_l_.^[^ ^7^ ^]^ Reducing the grain/particle size of TE materials from the bulk to the nanoscale is another practical way to increase the density of interfaces. By analogy to the “internal” nanostructuring of bulk TE materials, by employing defects across length scales, a wide size distribution of nanoparticles can facilitate the scattering of a wide spectrum of phonon modes. Figure 1b highlights some of the mechanisms of phonon (and charge carrier) interactions with nanoparticle arrays and with multiscale point and extended defects under a temperature gradient. An understanding of how different types of defects/nanostructures scatter different wavelength of phonons (i.e., the frequency‐dependence of phonon scattering rates) has advanced considerably in recent years. In bulk thermoelectric materials, the overall scattering rate of phonons is given by^[^ ^8^ ^]^

(4) $$\tau_{p}^{-1}=\tau_{\mathrm{PD}}^{-1}+\tau_{\mathrm{DC}}^{-1}+\tau_{\mathrm{DS}}^{-1}+\tau_{B}^{-1}+\tau_{U}^{-1}$$

Where each scattering process is frequency (*ω*) dependent. The subscript stands for scattering event: point defect scattering ($\tau_{\mathrm{PD}}^{-1}∼\omega^{4})$, dislocation scattering ($\tau_{\mathrm{DC}}^{-1}∼\omega^{3}$ and $\tau_{\mathrm{DS}}^{-1}∼\omega$ for dislocation cores and dislocation strains, respectively), boundary scattering$\left(\tau_{B}^{-1}∼\omega^{0}\right)$, phonon–phonon Umklapp scattering ($\tau_{U}^{-1}$∼*ω* ^2^). The boundary scattering of phonons targets low‐frequency phonons as its scattering rate is frequency independent $\left(\tau_{B}^{-1}∼\omega^{0}\right)$.^[^ ^8^ ^]^

However, the phonon frequency distribution varies with temperature implying in a certain temperature regime, one or more scattering event dominate. The frequency dependence of different scattering events gives us the opportunity to selectively scatter phonons of different wavelengths (without deteriorating the mobility, *μ*, of charge carriers) via rational design of nanostructured thermoelectric materials.^[^ ^9^ ^]^

As we will demonstrate later in this article, the nanostructuring of TEs can be achieved very effectively by judicious choices of synthesis and processing techniques and the design of such nanostructures can be realized to a high level of sensitivity without expending large amounts of energy. Solvothermal, solution‐based and ball‐milling (BM) methods are just some of the ways in which such scattering phenomena can be introduced and the performance of TE materials optimized.

### 1.2 Working Principle of Thermoelectric Devices and Their Applications

**Figure 2:**
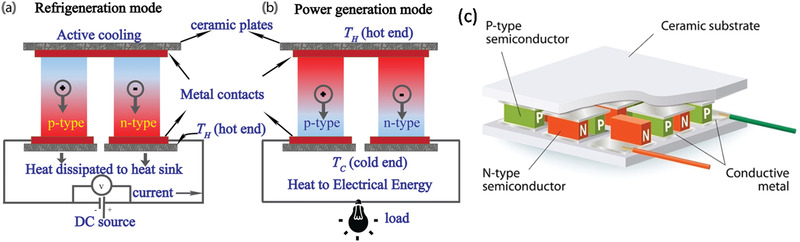
TE device in operation. a) Refrigeration mode; b) power generation mode; c) cascaded p–n pairs for large voltage output.

The typical basic unit of a thermoelectric device consists of a pair of cuboids of *n*‐type and *p*‐type semiconducting TE materials that are connected electrically in series and thermally in parallel with a temperature gradient as shown in **Figure** **2**a,b. Active cooling is achieved when a direct current (DC) current passes through this pair (Figure 2a). Other previously published articles describe the operation of thermoelectric devices in cooling and power generation modes.^[^ ^2^ ^]^ To generate appreciable power, many p–n pairs need to be cascaded to as shown in Figure 2c.

**Figure 3:**
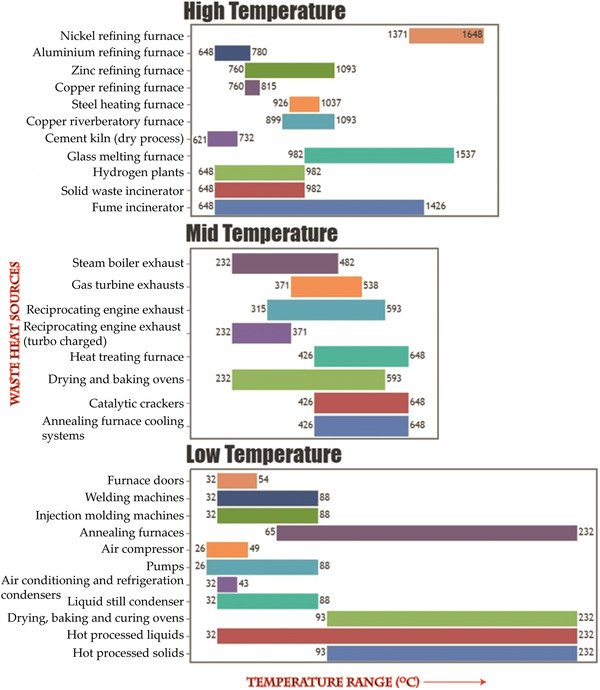
Grades of waste heats from different industrial processes. Data are adapted from ref. [12]. The colors of the bars have no particular meaning.

Medium and heavy industries (such as manufacturing, processing, and power generating plants) use thermal energy for heating and a major fraction of this energy is expelled into the external environment usually in the form of hot gases, steam, or water. **Figure** **3** depicts the grades(temperature range) of waste heat that are typical from a selected range of industrial activities.^[^ ^12^ ^]^ The production of iron and steel, basic chemicals, food, pulp, and paper nonferrous metals (mainly aluminum) and nonmetallic minerals (primarily cement) together with fuel refining can be considered to be archetypal energy‐intensive industrial processes. Collectively, these processes consume half of the energy delivered to all industrial sectors with the bulk chemical industry as the largest single consumer followed by refining and mining (EIA, 2019). This vast amount of currently wasted heat could be harnessed in several ways either to improve the overall energy efficiency of the respective industrial processes or to be stored and utilized as electrical energy elsewhere in other applications. The efficacy with which waste heat can be put to use relies upon several factors, such as: a) the degree of compatibility between the exhaust heat quality and that required for a particular application; b) the availability of copious quantities of waste heat to achieve economies of scale; c) the distance between the source of the waste heat and its ultimate point of use. Although in recent years the efficiency of energy use in industry has improved considerably compared to the 1990s, the magnitude of waste heat being expelled to the atmosphere is still large.^[^ ^13^ ^]^ Similarly, in fossil‐fuel‐based automotive engines, almost 60% of the spent energy is wasted as heat.^[^ ^14^ ^]^ Because the fleet of passenger vehicles is increasing year by year globally, the amassed volume of waste heat from these vehicles is rising precipitously. Moreover, these and other vehicles spew harmful greenhouse gases such as CO_2_ in large quantities. It is unsurprising, therefore, that the waste heat from the industrial and automotive sectors has become primary targets for thermoelectric waste heat recovery.^[^ ^15^ ^]^ A roof‐top solar thermoelectric generator (STEG) has the advantage to help the rural mass, who do not have easy access to power plant and electricity grid to drive their daily economic activities.^[^ ^16^ ^]^ Bi_2_Te_3_‐based nanostructured TE material coupled with and spectrally selective solar absorber STEG achieved a peak efficiency of 5.2% with a temperature difference of 180 °C (*T*_C_ = 20 °C, *T*_H_ = 200 °C).^[^ ^17^ ^]^ Similarly, doped Bi_2_Te_3_ and skutterudite materials based STEG assisted with solar energy concentrator showed a system efficiency of 7.4% and a peak efficiency of 9.6% with a maximum absorber temperature of 600 °C.^[^ ^18^ ^]^ Recently, a non‐Bi_2_Te_3_ 8‐pair TE module based on Mg‐Sb p‐ and *n*‐type materials exhibited 7.3% efficiency with a hot temperature side of 320 °C.^[^ ^19^ ^]^ Since most of the reported TE material efficiency is still below the expectation, there is a lot of room to improve the efficiency especially through energy‐saving routes.

Furthermore, the operation of a contemporary manufacturing enterprise relies on numerous types of prompt decision‐making at different levels and domains. It is a complex system with a large number of design variables that require real‐time data from machines, processes, and operations management. Therefore, the information technology (IT) infrastructure for data gathering, processing, and distribution can influence the performance and success of an enterprise greatly.^[^ ^20^ ^]^ It is in this context that a thermoelectric device‐powered internet‐of‐things (IoT) could play a crucial role in streamlining a given industrial process, utilizing ubiquitously available waste heat at high efficiency in the industrial environment.

Historically, distinct classes of TE materials have been developed that are suitable for operation across different ranges of temperatures (“high,” “mid,” and “low”).^[^ ^21^, ^22^ ^]^All, however, suffer from a common problem in that their thermal to electrical energy conversion efficiency is still generally very low in terms of what it is required for successful device design and deployment. Therefore, the thermoelectric performance of the component materials in devices needs further and considerable improvement (for example, via rational design or nanostructuring) and the synthesis and processing methods used to produce these materials must themselves be energy efficient. The material requirements for TE devices go beyond only the component TE materials; there are complex issues when it comes to device making. It is these bottlenecks that have been chiefly responsible for preventing the wide‐scale application of TE power generation.

Thermoelectric research is primarily focused on: a) developing TE materials using environmentally benign and Earth‐abundant raw materials; b) improving TE performance of existing materials via nanotechnological routes; c) developing TE modules with optimal output characteristics for specific applications.

This review article is categorized into two sections. 1) A brief overview of several of the classical state of the art TE materials. 2) A comprehensive review on the synthesis aspects of thermoelectric nanomaterials focusing on solvothermal/hydrothermal, microwave‐assisted, solution‐based, and powder processing methods along with a summary of recent work carried out in the field as well as the thermoelectric properties of the reported materials.

### 1.3 Some Prominent Thermoelectric Material Classes

Many distinct classes of TE material systems have been produced over the last few decades, and the reader is directed to a number of good and extensive review publications that address these various types of materials in detail: (Bi,Sb)_2_(Te,Se)_3,_^[^ ^23^ ^]^ SnSe,^[^ ^24^ ^]^ Cu_2_Se,^[^ ^25^ ^]^ and SnTe,^[^ ^26^ ^]^ PbTe,^[^ ^27^ ^]^ and skutterudites.^[^ ^28^ ^]^ These carefully selected materials are endowed with the electrical and thermal transport properties that synergically give rise to a figure‐of‐merit close to the universally accepted benchmark of *zT* = 1 or above. In this review, we focus on just five of these important classes of materials, selecting classes of established and emerging TE materials that might serve over each of the three traditionally identified application temperature ranges (“low‐,” “mid‐,” and “high‐”). Among them, for example, the group 15 metal chalcogenides (V‐VI compounds), which operate best at lower temperatures, are very suitable for wearable thermoelectric devices.^[^ ^29^ ^]^ In the later, main, sections of the review, we will present these five families as case studies when demonstrating how high‐performance TE materials can be successfully produced by sustainable means.

#### 1.3.1 (Bi,Sb)_2_(Te,Se)_3_‐Based Materials

**Figure 4:**
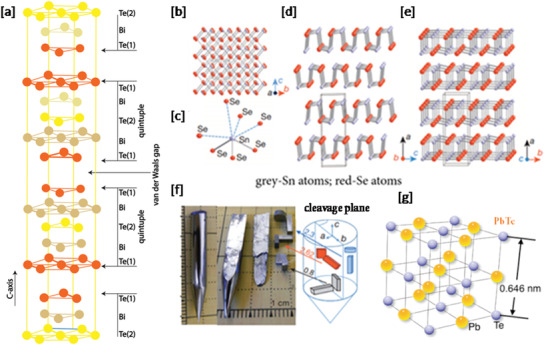
a) Schematic of Bi_2_Te_3_ crystal structure of D_3d_^5^‐*R*(‐3)*m* space group. b) SnSe crystal structure along the *a* axis; c) highly distorted SnSe_7_ coordination polyhedron with three short and four long Sn–Se bonds; d) SnSe structure along the *b* axis; e) structure along the *c* axis; f) SnSe crystals (left side); samples cut along the three axes (right side). Adapted with permission.^[^ ^46^ ^]^ Copyright 2014, Nature. g) Crystal structure of PbTe.

Bismuth telluride (Bi_2_Te_3_) is a semiconductor with an indirect bandgap of approximately 0.15 eV and is the most important and widely used TE material for room temperature (RT) applications. It can be alloyed either with antimony (Bi_2‐_ *_x_* Sb*_x_* Te_3_; *x* ≈ 1.5) or selenium (Bi_2_Te_3‐_ *_y_* Se*_y_*; *y* ≈ 0.3) to deliver *p*‐type or *n*‐type TE materials, respectively and both materials exhibit *zT* ≈ 1. The best performing bulk (nanostructured) *p*‐type Bi_2_Te_3_ material was reported with *zT* = 1.4 at 300 K.^[^ ^30^ ^]^ This material, like many TE materials, was prepared by ball‐milling the Bi_0.5_Sb_1.5_Te_3_ alloy ingots followed by hot‐pressing. The enhancement in *zT* was attributed to reduced *κ* and a PF comparable to bulk *p*‐type Bi_0.5_Sb_1.5_Te_3_. Bi_2_Te_3_ crystallizes as plates with a rhombohedral structure (*a* = 0.4384 nm and *c =* 3.045 nm) comprising five monoatomic layers (Te(1)‐Bi‐Te(2)‐Bi‐Te(1)), often denoted as “quintuple layers,” which are stacked by van der Waals interactions along the *c*‐axis of the unit cell.^[^ ^31^, ^32^ ^]^ The indices (1) and (2) denote two different crystallographic sites (chemical environments) for the Te^2–^anions. The presence of the van der Waals gap between the quintuple layers gives rise to an easy cleavage plane between the adjacent Te(1) layers as shown in **Figure** **4**a.

Sb_2_Te_3_ has also attracted considerable technological interest because of its potential applications in mini power‐generation systems, charge‐coupled device (CCD) technology, microcoolers, and infrared detectors.^[^ ^33^ ^]^ Similar to Bi_2_Te_3_, Sb_2_Te_3_ is composed of layers of similar atoms following the sequence of a quintet ‐Te(1)–Sb‐Te(2)‐Sb‐Te(1). These layers once more stack up along the *c*‐axis of the Sb_2_Te_3_ unit cell and as with Bi_2_Te_3_, the functional properties of antimony telluride are anisotropic as a result. Sb_2_Te_3_ is also classed as a *p*‐type 3D topological insulator^[^ ^34^ ^]^ (insulators having metallic states on their surfaces but insulating states in the interior) which find applications in spintronics and quantum computing.^[^ ^35^ ^]^ Over two decades ago it was predicted that the quantum confinement of electrons in 2D Bi_2_Te_3_ layers can enhance the PF.^[^ ^36^ ^]^ Experimental observation of surface electronic states assisted by time‐reversal symmetry in Bi‐chalcogenides (Bi_2_Te_3_, Bi_2_Se, and BiSbTeSe) has also led to the theoretical prediction of enhancement in TE in ultrathin layers of so‐called topological insulators.^[^ ^37^ ^]^ That can be done with fine control of the Fermi level and carrier concentration. Several methods have been proposed to optimize both carrier concentration and Fermi energy in ultrathin samples. These techniques entail doping of Ca^[^ ^38^ ^]^ and Sb^[^ ^39^ ^]^ on the Bi atomic sites in order to optimize the Fermi energy and maximize the contribution from the protected surface states. To tune the Fermi energy in Bi_2_Te_3_ involve doping with Sb^[^ ^39^ ^]^ to create (Bi_1−_ *_x_* Sb*_x_*)_2_Te_3_ in the range between *x* = 0 (Bi_2_Te_3_, electrons rich samples) and *x* = 1 (Sb_2_Te_3_, hole rich samples). In that direction, thin (Bi_1−_ *_x_* Sb*_x_*)_2_Te_3_ nanoplates with *x* ranging from 0.07 to 0.95 were prepared.^[^ ^40^ ^]^ The carrier concentration in the nanoplates was changed by Sb substitution, whereas the surface potential was tuned by direct deposition of a thin layer of F4‐TCNQ (hole‐injecting molecule), onto all surfaces of individual suspended nanoplates. In F4‐TCNQ‐surface doped (Bi_1−_ *_x_* Sb*_x_*)_2_Te_3_ nanoplates, a maximum *p*‐type *S* (129 µV K^−1^), and corresponding maximum *zT* (0.33 at ≈350 K) at *x* = 0.5 and a general *p*‐type doping effect upon exposure to F4‐TCNQ was observed. The surface effect was enough to allow observation of majority carriers from electrons to holes in a lightly doped (*x* = 0.0), 9 nm thick sample.^[^ ^39^, ^40^ ^]^ Multiscale nanostructured *p*‐type (Bi_0.48_Sb_1.52_Te_3_) bulk material, with 10–20 nm nanocrystals with coherent grain boundaries were prepared from the precursor single elements via single‐element‐melt spinning followed by a SPS method.^[^ ^41^ ^]^ This method yielded a *κ*_l_ of 0.4–0.5 W m^−1^ K^−1^ between 300 and 400 K (compared to 0.8–0.9 W m^−1^ K^−1^ for the zone‐melted (ZM) sample) and a *zT* of ≈1.5 at 390 K.

Recently, a novel technique for improving the TE performance of skutterudites was presented, in which magnetic nanoparticles were incorporated into the skutterudites matrix to optimize both the electrical and thermal conductivities of the resulting nanocomposites.^[^ ^42^ ^]^ It was determined that magnetic nanoparticles had three major effects on the TE characteristics of their materials. First, each nanoparticle works as a reservoir for free electrons, boosting *σ*. Second, the oscillating superparamagnetic moments scatter electrons preferentially, increasing the *S*. Finally, phonons are scattered by the presence of randomly dispersed nanoscale particles. Subsequently, this approach was tested for (Bi,Sb)_2_(Te,Se)_3_ based TE materials. For example, incorporation of 0.4 mol% Ni nanoparticles in Bi_2_Te_2.7_Se_0.3_ nanoplate sample simultaneously increased the carrier concentration and provided additional scattering centers, which resulted in enlarged *σ*s and *S*s, finally yielding a *zT* of 0.66 at 425 K (43% increase as compared to pure Bi_2_Te_2.7_Se_0.3_ nanoplates).^[^ ^43^ ^]^ Similarly, the incorporation of 0.15 wt% Fe_3_O_4_ nanoparticles in Bi_0.5_Sb_1.5_Te_3_ sample simultaneously increased the *S* and enhanced phonon scattering, which resulted in a 32% enhancement of *zT* to 1.5 at 340 K.^[^ ^44^ ^]^ Many reports suggest that incorporation of magnetic nanoparticles in TE materials can be a good strategy to enhance the TE performance.^[^ ^42^, ^45^ ^]^

Here have been given several classical examples of nanostructuring of (Bi,Sb)_2_(Te,Se)_3_ based materials leading to high *zT*s, and in the main part of the review, we will review and discuss energy‐saving pathways for such thermoelectric nanomaterials synthesis.

#### 1.3.2 Lead and Other Metal Chalcogenides

##### Pb*X*

Rock‐salt structured, lead chalcogenides Pb*X* (IV–VI compounds with *X* = Te, Se, S and) are narrow bandgap semiconductors with a history of use as sensors for infrared radiation, photoresistors, and lasers in addition to finding application in thermoelectric devices.^[^ ^47^ ^]^ For several decades PbTe (Figure 4g) was the premier TE material for mid‐range (400–800 K) applications^[^ ^48^ ^]^ including its successful deployment in deep space missions.^[^ ^49^ ^]^ PbSe has a higher melting point (1355 K) compared to PbTe (1253 K) and is therefore suitable for higher temperature applications including solar energy conversion. PbTe can be prepared into *n*‐type or *p*‐type TEs using suitable dopants. The alkali metals, Li, Na and K are typically used as acceptor dopants at the Pb site to produce *p*‐type materials, whereas either halides (I and Br) or Sb, Bi, Al, Ga, and In are doped at the Te or Pb site, respectively to produce *n*‐type TEs. The *κ*_l_ of PbTe is relatively low, measuring ≈2.2 W m^−1^ K^−1^ at RT and this can be reduced even further via nanostructuring.^[^ ^50^ ^]^ One of the main drawbacks of PbTe‐based TE compounds, however, is the use of highly toxic lead, which has considerable impacts on both the environment and human health.^[^ ^51^ ^]^ Moreover, tellurium has a low abundance in the Earth's crust, which places it among the less sustainable elements, with both environmental and economic consequences.^[^ ^52^ ^]^ These shortcomings reduce the likelihood of these compounds becoming viable TE materials and limit their application. Although there is as yet no completely satisfactory replacement for lead (but see the tin chalcogenides below), tellurium can be replaced by selenium without hugely impacting on the performance of the material. Se is inexpensive, has longer‐term price stability, and is 50 times more abundant than Te.^[^ ^52^ ^]^ Consequently, research is being actively pursued on PbSe as an alternative TE.^[^ ^53^ ^]^

##### SnX (X = *S, Se, Te*)

Tin‐based binary chalcogenide compounds are also “IV‐VI” compounds and the most promising are also those of a 1:1 stoichiometry, Sn*X* (*X* = S, Se, Te). The monochalcogenides, tin selenide (SnSe),^[^ ^46^, ^54^ ^]^ tin sulfide (SnS),^[^ ^55^ ^]^ and tin telluride (SnTe)^[^ ^56^ ^]^ have attracted considerable interest given their propensity for exceptional thermoelectric performance without the disadvantages associated with toxic heavy metals, such as lead. Whereas SnTe adopts a cubic rock salt structure analogous to its lead equivalent, the Sn*X*(*X* = S, Se) compounds form layered structures and have the advantage of containing both non‐toxic and Earth‐abundant metals. SnSe forms an orthorhombic layered structure (itself a distorted rock‐salt structure) which transforms subtly from a primitive (space group *Pnma*) to a base‐centered (space group *Cmcm*) unit cell at high temperature (≈800 K), a process that involves a slight distortion of the Sn–Se layers. At elevated pressure, the orthorhombic form transforms into a monoclinic polymorph (at ≈12 GPa). At RT and pressure, the orthorhombic SnSe structure contains SnSe slabs with a thickness of two atoms with strong Sn–Se bonds within the *b–c* plane of slabs, and weaker bonds along the *a*‐axis (Figure 4b). The perspective view along the *b*‐axis can be viewed as a zigzag, accordion‐like projection and as an “armchair” form along the *c*‐axis.^[^ ^24^, ^57^ ^]^ SnSe has an exceptionally low *κ*_l_ of 0.23  ±  0.03 W m^−1^ K^−1^ at 973 K (owing to a high Gruneisen parameter) yielding an unequaled *zT* value of 2.6 at 923 K in single crystals aligned along the *b*‐axis, and a *zT* of 2.3 along the *c*‐axis. By contrast,  *zT*  = 0.8 along the *a*‐axis, emphasizing the extreme anisotropy in the structure and properties of the chalcogenide.^[^ ^46^ ^]^ Although single‐crystalline SnSe possesses exceptional TE properties, its synthesis as dense ingots cannot be achieved without recourse to a highly energy‐intensive stepwise process, including Bridgman crystal growth.^[^ ^58^ ^]^

##### Copper Tellurides (Cu*_x_* Te)

Among other metal chalcogenides, copper tellurides, selenides, and sulfides are perhaps attracting the greatest level of interest as TEs that could possibly rival the SnX materials. Copper tellurides (Cu*_x_* Te, 1 < *x* < 2) have proven to be worthy of study not only for their inherently high TE PFs^[^ ^59^ ^]^ but also on account of their direct bandgaps (≈1.1–1.5 eV)^[^ ^60^ ^]^ and superionic conductivity.^[^ ^61^ ^]^ A high‐density Cu_2_Te sample can be obtained via direct annealing without a sintering process. With direct annealing, sample's composition could be well controlled to optimized carrier concentration and thermal conductivity could be reduced, finally leading to improved *zT* ≈ 1.1 at 1000 K. This approach shortens the synthesis time and lowers the cost of sample growth.^[^ ^62^ ^]^

##### Copper Selenides (Cu_2_Se) and Copper Sulfides (Cu_2‐_ *_x_* S)

**Figure 5:**
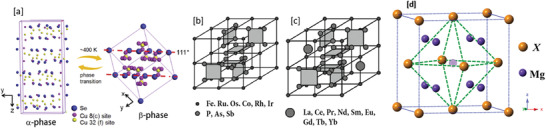
a) The structures and phase transition between *α*‐Cu_2‐_ *_x_* Se and *β*‐Cu_2‐_ *_x_* Se. Reproduced with permission.^[^ ^63^ ^]^ Copyright 2016, Elsevier. b,c) Crystal structure of skutterudites: b) unfilled; c) filled. Reproduced with permission.^[^ ^64^ ^]^ Copyright 2015, Pleiades Publishing, Ltd.; d) Crystal structure of Mg_2_ *X* (*X* = Si, Ge, Sn). Dashed circle position is potentially occupied by interstitial atoms. Adapted with permission.^[^ ^65^ ^]^ Copyright 2015, Wiley‐VCH.

Environmentally benign copper selenide (Cu_2_Se, energy gap of 1.23 eV), adopts rather complex crystal structures as manifested by two phases: a low‐temperature *α*‐phase and a high‐temperature *β*‐phase. The *β*‐phase of Cu_2_Se is an established Cu^+^ superionic conductor.^[^ ^66^ ^]^ As shown in **Figure** **5**a, the *α*‐phase of Cu_2_Se has a monoclinic crystal structure at RT (space group *C*2/*c*, *a* = 0.7138 nm, *b* = 1.238 nm, *c* = 2.739 nm, and *β* = 94.308°) with Cu ions located in 12 in equivalent crystallographic positions.^[^ ^67^ ^]^ However, above 400 K, *α*‐Cu_2_Se transforms into a cubic FCC structured *β*‐phase (space group $Fm\bar{3}$ *m*; *a* = 0.58 nm).^[^ ^67^ ^]^ In *β*‐Cu_2_Se, Cu^+^ ions partially occupy the 8(c) and 32(f) interstitial sites leading to a superionic liquid‐like behavior with high mobility (*μ*) within the {111} planes of the close packed Se sublattice.^[^ ^68^ ^]^ High *zT* values have been recorded in polycrystalline bulk ingots, however as with SnSe, the techniques of production need a large amount of energy input over multiple steps, including melting followed by spark plasma sintering.^[^ ^68^ ^]^ Cu_2_Se ingots registered *PF*s of ≈6–8 µW cm^−1^ K^−2^ for the *α*‐phase and 7–12 µW cm^−1^K^−2^ for the *β*‐phase (over a temperature range of 420–1000 K). Due to the very low *κ*_l_ exhibited by Cu_2_Se (0.4–0.6 W m^−1^ K^−1^), *zT* values of ≈1.5 could be reached at 1000 K. Cu ion migration has been an obvious problem when considering TE applications of Cu_2_Se and related materials. There have been recent efforts to address this problem, such as utilizing a barrier layer^[^ ^69^ ^]^ and graphene. Such studies will be discussed in the synthesis section. The many attractive physical properties of Cu_2_Se have resulted in the material's consideration for several other applications besides as a component for TE devices, namely in optical filters^[^ ^70^ ^]^ (due to Cu_2_Se thin film's transparency and easy deposition) and solar cells.^[^ ^71^ ^]^ Cuprous chalcogenide films of Cu_2_S, Cu_2_Se, and Cu_2_Te are generally *p*‐type and can form highly conductive semitransparent films. The junction formed by vacuum evaporation of a Cu_2‐_ *_x_* Se film onto an *n*‐type semiconductor is found to have excellent photovoltaic properties.^[^ ^72^ ^]^

Copper sulfides (Cu_2‐_ *_x_* S (0 ≤ *x* ≤ 1)), also exist with different Cu stoichiometries, varying from Cu‐rich Cu_2_S to Cu‐deficient CuS. Compared to mainstream PbTe based materials, the sulfides also less impactful on the environment and also carry the benefit of being relatively inexpensive. In fact the sulfides have multiple uses as functional and energy materials in solar cells, lithium batteries, and optoelectronic devices.^[^ ^73^ ^]^ Among the copper‐rich Cu_2‐_ *_x_* S compounds, Cu_1.8_S (digenite; bandgap ≈1.5 eV) is notable as a steady phase,^[^ ^73^ ^]^ in which two types of digenite Cu_1.8_S phases exist, in fact, depending on temperature. These are distinguished as so‐called low‐digenite and high‐digenite, respectively with a phase transition temperature at ≈364 K^[^ ^74^ ^]^ (i.e., at RT, the stable phases are djurleite (Cu_1.97_S), anilite (Cu_1.75_S) and covellite (CuS)). Above 364 K, anilite decomposes to high digenite and covellite.^[^ ^75^ ^]^ Above 364 K, the Cu atoms in the crystal structure switch from ordered to disordered arrangements.^[^ ^76^ ^]^ The structure of the disordered high‐digenite phase offers a low activation barrier for Cu^+^ ion motion, indicative of liquid‐like behavior (ostensibly similar to the improved cationic mobility in *β*‐Cu_2_Se, which is the kinetically stable phase of bulk Cu_2_Se). The crystals of the digenite are found to be cubic or hexagonal based on the fcc sublattice of S. The structure of the high‐digenite phase is similar to that of cubic Cu_2_S (chalcocite), where S atoms maintain a rigid sublattice, while only 9/10 of the Cu sites are occupied.^[^ ^74^ ^]^ With *x* > 0, Cu_2−_ *_x_* Se is a good *p*‐type electrical conductor with a high Seebeck coefficient and its *σ*increases with increasing Cu deficiency.^[^ ^68^ ^]^ Measurements of transport reveal that a Cu deficiency results in an equivalent concentration of holes.^[^ ^77^ ^]^ The *β*‐Cu_1.75_Se has a mixed conduction and at RT, the Cu ion conductivity is found to be 3 × 10^–2^ Ω^−1^ cm^−1^.^[^ ^78^ ^]^ The nonstoichiometric, copper‐rich sulfides, Cu_1.8_S and Cu_1.97_S, each prepared by the direct melting of starting materials, yielded *zT* values of 1.4 and 1.7 at 1000 K, respectively.^[^ ^25a^ ^]^

As pointed out above, the liquid‐like behavior of materials such as Cu_2_Se and Cu_2‐_ *_x_* S, can be an obstacle for thermoelectric applications. There has been significant research carried out on stable Cu based sulfides.^[^ ^79^ ^]^ Relatively early work on CuFeS_2_ chalcopyrite, for example, showed high *PF*s were achievable at RT and indicated that these were enhanced by magnetic interactions.^[^ ^80^ ^]^ Similarly, carrier doping of CuCr_2_S_4_ led to relatively high TE performance.^[^ ^81^ ^]^ High *zT* was achieved in Cu sulfides like tetrahedrites,^[^ ^82^ ^]^ and colusites.^[^ ^83^ ^]^

#### 1.3.3 Skutterudites

Skutterudites represent classic examples of the concept of a phonon glass–electronic crystal (PGEC) and comprise a class of compounds with isotypic crystal structures such as CoAs_3_, CoP_3_, CoSb_3_, IrSb_3_, RhP_3_, RhAs_3_, RhSb_3_, IrP_3_, and IrAs_3_, generally denoted by “*M*X_3_.” The unit cell contains 8 transition metal atoms and 24 p‐block elements from group 15 (the pnictides).^[^ ^64^ ^]^ The cell can be divided into eight octants, of which 6 octants are occupied by pnictides leaving two octants empty (Figure 5b). These voids or empty spaces can be filled with different elements: either lanthanides, actinides, alkaline‐earth metals, alkali metals, thallium or the metalloids from Group 14.^[^ ^28^, ^84^ ^]^ These void filling atoms not only act as electron donors or acceptors leading to optimum carrier concentrations but also they act as “rattlers” enhancing the phonon scattering and reducing the *κ*_l_.^[^ ^28^, ^84^ ^]^ The *κ* of native, unfilled skutterudites is relatively high (8.9 W m^−1^ K^−1^ at 300 K) and therefore not suitable for practical implementation as a TE. Many of the research efforts directed at skutterudites are therefore focused on reducing this intrinsic thermal conductivity as a means to achieving a higher *zT*.^[^ ^85^ ^]^ Modifying CoSb_3_, for example, with multiple fillers (atoms of different masses), instills a significant reduction in *κ*_l_ up to the glass limit.^[^ ^86^ ^]^ Hence, CoSb_3_ with a high electrical conductivity and high Seebeck coefficient is capable of achieving a *zT* of ≈1 with appropriate doping.^[^ ^87^ ^]^ Whereas at RT, *zT* for skutterudites is small, with typical values of ≈0.2, at elevated temperature, *zT* for these materials can increase to ≈1.4 (for CeFe_4−_ *_x_* Co*_x_* Sb_2_ at 1000 K, for example).^[^ ^88^ ^]^ Therefore, these materials are particularly useful in high temperature TE applications.^[^ ^64^ ^]^ Unfilled skutterudite alloy Co_23.4_Sb_69.1_Si_1.5_Te_6.0_ containing all scale, irregular and randomly oriented pores (tens of nm to few μms), exhibited a *κ* ≈ 1.4 W m^−1^ K^−1^ and *zT* ≈ 1.6 at 773 K. The large reduction in *κ* was ascribed to randomly oriented pores that provided large scattering cross section for a wide spectrum of phonons.^[^ ^89^ ^]^ The mechanical properties of skutterudites has also been actively investigated with an aim to make effective modules.^[^ ^90^ ^]^

Zhao et al. have shown that the insertion of soft magnetic Co nanoparticles into a CoSb_3_ type material (Ba_0.3_In_0.3_Co_4_Sb_12_) has been found to affect the phonon and electron‐transport characteristics.^[^ ^42^ ^]^ When Co nanoparticles account for 0.2% of the overall mass of the material, the *zT* rises from around 1.3 to ≈1.8. In *x*BaFe_12_O_19_/In_0.25_Co_4_Sb_12_ magnetic nanocomposites, BaFe_12_O_19_ (permanent magnetic material) nanosuspension is shown to suppress the high‐temperature performance deterioration of In‐filled CoSb_3_ material.^[^ ^91^ ^]^

#### 1.3.4 SiGe Alloys

Recent studies have shown the relevance of relating the material's price, its conversion efficiency, and manufacturing costs when evaluating different TE materials for a specific application.^[^ ^92^ ^]^ At this juncture, silicon‐germanium (SiGe) based alloys could overcome this bottleneck by providing large power density, low toxicity, and cost‐efficiency. Silicon is the second most prevalent substance on the planet's surface. SiGe alloys crystallize in a zinc blend structure. In GeSi alloys, *μ*_e_ and *μ*_h_ and *κ* show a typical U‐shape compositional dependence having a minimum at *x* = 0.5–0.8. The energy gap of SiGe alloys can be tuned (1.12–0.67 eV) by changing the composition and therefore these alloys find applications in photovoltaics,^[^ ^93^ ^]^ transistors,^[^ ^94^ ^]^ optoelectronic‐photonic devices,^[^ ^95^ ^]^ and have been investigated for their thermoelectric properties (*zT* ≈ 1–1.9)^[^ ^96^ ^]^ for high‐temperature applications.^[^ ^97^ ^]^ Apart from attractive TE and physical properties, SiGe devices can operate at temperatures up to ≈ 1050 °C without significant power degradation.^[^ ^98^ ^]^ SiGe alloys are characterized by a high‐melting point, good chemical stability, a low coefficient of thermal expansion mismatch, and high mechanical strength.^[^ ^99^ ^]^ Radioisotope thermoelectric generators (RTGs) based on SiGe have been employed in deep‐space applications,^[^ ^98^, ^100^ ^]^ automotive waste heat recovery,^[^ ^15a^ ^]^ and other applications.^[^ ^101^ ^]^ The compatibility of SiGe alloys with complementary metal‐oxide semiconductor (CMOS) technology is a big advantage. Because SiGe alloys have ultrahigh frequency capabilities, they can be utilized in circuits that operate at frequencies well beyond 100 GHz. In the past, several methods have been developed to synthesize SiGe alloys: grain‐refined alloys,^[^ ^102^ ^]^ nanoinclusions,^[^ ^103^ ^]^ SiGe superlattices fabrication,^[^ ^104^ ^]^ high‐energy ball milling,^[^ ^105^ ^]^ spark plasma sintering,^[^ ^106^ ^]^ chill casting method,^[^ ^1^ ^]^ etc. A *zT* of ≈1.3 at 1173 K was observed in a hot‐pressed *n*‐type nanostructured dense SiGe.^[^ ^96c^ ^]^ A *zT* of ≈1.5 at 1173 K was reported for SPSed *n*‐type SiGe alloys.^[^ ^99a^ ^]^ Nanostructured *n*‐type SiGe bulk alloy (prepared via ball milling and hot pressing) exhibited a *zT* of ≈1.84 at 1073 K.^[^ ^96a^ ^]^ Optimized sample exhibited a *S* of ≈284 µV K^−1^, resistivity (*ρ*) ≈ 45 μΩ m and a *κ *of ≈ 0.93 W m^−1^ K^−1^ at 1073 K. The improved TE performance in these materials was attributed to a decrease in *κ*_l_ as a result of nanostructuring. Boron and phosphorus are found to have high solubilities in SiGe; therefore they are useful as *p*‐type and *n*‐type impurities, respectively. At 1200 K, the maximum values of *zT* were 0.8 for *p*‐type Ge_0.15_‐Si_0.85_ alloy (doped to 2.1 × 10^20^ cm^−3^ holes), and 1.0 for *n*‐type Ge_0.15_–Si_0.85_ alloy (doped to 2.7 × 10^20^ cm^−3^ electrons) and 4 W m^−1^ K^−1^ for a highly doped *n*‐type Si_0.7_Ge_0.3_ alloy as compared to 150 W m^−1^ K^−1^ for pure Si. The maximum over‐all efficiency of a stable generator operating between 300 and 1200 K was estimated to be 10%.^[^ ^97a^ ^]^

Many reports show that SiGe‐based TE materials exhibit lower *κ* (≈6 W m^−1^ K^−1^) due to alloy scattering than bulk Si.^[^ ^107^ ^]^ This *κ* is far too large to be used in practical TE device applications. In SiGe nanowires (NWs) a minimum *κ* of 1–2 W m^−1^ K^−1^ and a *zT* of 0.46 at 450 K (which is two times that of a bulk alloy with the same composition) are reported.^[^ ^108^ ^]^ Si_0.8_Ge_0.2_ nanomeshed films fabricated via DC sputtering showed a low *κ* = 0.55 ± 0.10 W m^−1^ K^−1^, which is well below the amorphous limit.^[^ ^109^ ^]^ It has been demonstrated that SiGe NWs based μTEGs can generate a power density of 7.1 µW cm^−2^ when the waste heat source temperature is ≈200 °C.^[^ ^101^ ^]^ It has been shown that Si in powder‐form absorbs microwave energy. Therefore, microwaves can be used as an energy source to heat Si and Ge in combination to fabricate SiGe alloys. Findings from such studies are presented in Section 2.3.6.

#### 1.3.5 Mg_2_ *X* (*X* = Si, Ge, Sn) based TE compounds

Mg_2_ *X* (*X* *=* Si, Ge, Sn) intermetallic alloys are being investigated to be one of the most promising classes of TE materials for mid‐temperature (600 < *T* < 1200 K) power generation.^[^ ^110^ ^]^ The constituent elements of these materials are ecofriendly, cheap, and abundant.^[^ ^111^ ^]^ Among the Mg_2_ *X* series, Mg_2_Si has a low density, *d* (1.88 g cm^−3^) and the ratio (*zT*/*d*) for Mg_2_Si is the highest among commercial TE materials.^[^ ^110b^ ^]^ The bandgap decreases with an increase in the atomic mass of *X* (from 0.8 eV for Mg_2_Si to 0.35 eV for Mg_2_Sn).^[^ ^111^, ^112^ ^]^

Due to narrow energy gaps, Mg_2_Sn and Mg_2_Si analogs have been considered as good candidates for infrared optoelectronic devices.^[^ ^113^ ^]^ Mg_2_ *X* compounds crystallize in a face centered cubic (fcc) lattice, CaF_2_‐type structure (space group F*m*3*m*),^[^ ^114^ ^]^ where the F atom is replaced by the Mg atom and the Ca atom is replaced by the *X* atom (Figure 5d). Each atom of the *X* group is surrounded by eight Mg atoms in a regular cube. The bond in all these compounds is covalent. Mg_2_Ge has the highest *μ*_e_ of ≈530 cm^2^ V^−1^ s^−1^ and Mg_2_Sn has the highest *μ*_h_ of ≈260 cm^2^ V^−1^ s^−1^ at RT.^[^ ^110b^ ^]^

Members of the Mg_2_ *X* series form solid solutions with varying degrees of solubility with one another. The Mg_2_Si–Mg_2_Sn system is widely studied due to its high *zT*. Mg_2_ *X* with 1.1 < *zT* < 1.5 has been reported for the *n*‐type materials with the *p*‐type showing dismal performance.^[^ ^115^ ^]^ The poor TE performance of the *p*‐type Mg‐based TE materials poses serious limitations on the fabrication of devices, and most reports on the use of these materials in modules are of *n*‐type legs.^[^ ^116^ ^]^

In Mg_2_ *X* with *X* = Si, Ge, and Sn, the *κ*_l_ is found to be very high: ≈7.9, 6.6, and 5.9 W m^−1^ K^−1^ respectively.^[^ ^117^ ^]^ There are several energy‐intensive methods to fabricate Mg_2_ *X* compounds.^[^ ^118^ ^]^ Direct comelting is one of them.^[^ ^119^ ^]^ This method has some limitations associated with the large disparity in the melting temperature of components and the high vapor pressure of Mg.^[^ ^110b^ ^]^ Therefore, attention is necessary to minimize Mg losses due to component evaporation (Mg_2_Sn in particular). Another issue is related to the large difference in the masses of Mg, Si, Ge, and Sn atoms. To avoid segregation by specific weight, stirring is required. Long‐term annealing is required to achieve homogenous alloys. To shorten the annealing time, hot pressing may be utilized, adding another step to the synthesis process. Ingots of alloys are then pulverized into powder and then pressed in a vacuum. Annealing, on the other hand, is not necessary for samples made from nanosized particles. Mg_2_Si, undoped and doped with Bi and Ag, were grown by a vertical Bridgman method.^[^ ^120^ ^]^ The Bi‐doped (*n*‐type) samples exhibited a *zT* of 0.65 at 840 K, while Ag‐doped (*p*‐type) samples showed a maximum *zT* of 0.1 at 570 K. Induction melting, melt spinning, and spark‐plasma‐sintering (SPS) methods in combination were used to produce *n*‐type Mg_2_Si_0.4_Sn_0.6_ alloys doped with Bi.^[^ ^115d^ ^]^ Methods such as solid‐state reactions,^[^ ^118a^ ^]^ arc‐melting,^[^ ^121^ ^]^ and SPS,^[^ ^118^, ^122^ ^]^ are time consuming, involve several steps, energy intensive, riddled with problems such as Mg volatilization and reaction with the sample holder.

A theoretical study predicts the influence of nanostructuring on the TE performance of n‐Mg_2_Si and n‐Mg_2_Si_0.8_Sn_0.2_ alloys.^[^ ^123^ ^]^ It was shown that the concomitant effect of a large depression in *κ*_l_ and decrease in *σ* due to grain boundary scattering can lead to 10% and 15% increase in *zT* at 850 K in nanostructured Mg_2_Si and Mg_2_Si_0.8_Sn_0.2_, respectively. Similarly, nanoinclusions (≈3.4%) of Mg_2_Si and Mg_2_Ge in the n‐Mg_2_Si_0.4_Sn_0.6_ matrix predicted a considerable decrease in *κ* (60% reduction of *κ* at 300 K and to 40% at 800 K) and increase in the *zT*. The best value of *zT* ≈ 1.9 at 800 K is expected for Mg_2_Si or Mg_2_Ge nanoparticles in Mg_2_Si_0.4_Sn_0.6_.^[^ ^124^ ^]^ Therefore, large scale synthesis of nanostructured alloys fabricated via a solution‐based route is more favorable for achieving a higher *zT*. Recently, MW‐solid‐state synthesis of bulk Mg_2_Sn and Mg_2_Si materials are reported. The underlying principles and recent reports on these synthesis techniques will be discussed in Section 2.3.6.

When it comes to practical TE devices, an increase in thermo‐element's TE efficiency over the whole service temperature range is necessary. Understanding and regulating the mechanical characteristics of TE materials are critical for realizing practical TEGs, especially for bulky conventional devices. The poor mechanical strength of TE materials not only causes failure during element production but also restricts the degree of miniaturization. Mechanical parameters such as elastic modulus, strength, hardness, fracture toughness, and thermal fatigue resistance must be maximized over the whole working temperature range. The elastic constants decide the stiffness of the material, whereas the strength describes the loading conditions under which the material will retain its original shape. The fracture toughness of a material determines the magnitude of the stress envelope in which it may function as well as its susceptibility to intrinsic fabrication flaws. The mechanical properties of the material are mostly determined by the atomic bonding between the atoms that comprise it. Engineering the material microstructure at the micron and submicron dimensions can improve material strength. Further, postsynthesis treatment such as densification methods will also play a very important role in deciding the mechanical properties.

The addition of nanoparticles, the formation of nanoprecipitates in the bulk matrix, doping, or alloying have all been proven to affect the hardness and/or elastic modulus of a TE material (Section 2.3.10). Literature on the mechanical properties of materials fabricated via soft chemistry approaches is scarce, and therefore, this article does not concentrate on the mechanical properties of materials obtained using such approaches.

## 2 Traditional versus Green, Sustainable TE Material Synthesis and Processing Methods

### 2.1 Bulk TE Materials

Advanced inorganic materials, which include the vast majority of metallurgical materials, semiconductors, and other materials, have found use in structural, mechanical, chemical, electrical, electronic, optical, photonic, and medical applications. These materials have typically been manufactured industrially using so‐called high‐technology, in which high temperature, high pressure or vacuum, molecule, atom, ion, plasma, and other processes have been used to produce desired products. In particular semiconductor and ceramic materials face difficulties in shape formation and fixing because of their intrinsic rigidity and brittleness. The technology required for the production of such materials demands fast and large production where a large amount of energy based on fossil‐fuels is needed. However, the fossil‐fuels reserve is limited on earth's crust and therefore continuous investment of fossil‐fuels in fabrication advanced materials is not sustainable.

### 2.2 TE Thin Film Deposition Methods

Recent inorganic TE thin films processing techniques can be beneficial for producing shaped material in a single step. However, some of these techniques demand more total energy than typical high‐temperature procedures due to their vacuum systems necessitating continuous pumping. Furthermore, controlling the stoichiometry of film is challenging. These include chemical vapor deposition (CVD),^[^ ^125^ ^]^ metal organic chemical vapor deposition (MOCVD),^[^ ^126^ ^]^ and physical vapor deposition (PVD), sputtering,^[^ ^2^ ^]^ pulsed laser deposition (PLD),^[^ ^127^ ^]^ molecular beam epitaxy (MBE),^[^ ^2^, ^128^ ^]^ ion beam sputter deposition (IBSD),^[^ ^129^ ^]^ flash evaporation,^[^ ^130^ ^]^ coevaporation,^[^ ^131^ ^]^ hybrid physical chemical vapor deposition (HPCVD),^[^ ^132^ ^]^ and electrochemical deposition.^[^ ^133^ ^]^ These TE thin film methods can have advantages such as integration possibilities into thin film devices and miniaturization of TE legs as regards to IoT energy harvesting applicative possibilities.^[^ ^134^ ^]^ However, regarding the topic of this review, many of these methods are not suitable for a large‐area film deposition and many of them are not cost‐effective in regards to synthesis of relatively large scales of material.

### 2.3 Green Synthesis: Alternative Sustainable Synthesis Approaches to TE Materials

**Figure 6:**
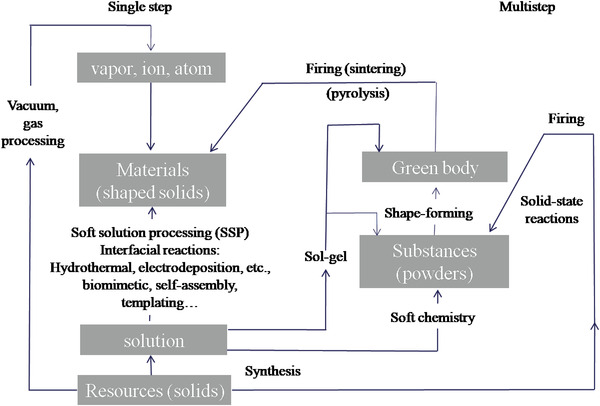
Schematic diagram of advanced‐materials processes illustrating the flow for single step versus multistep processes. The processes on the right side are environmentally and energy stressing and involve multiple steps. Soft solution processing (SSP) aims to fabricate shaped materials in (preferably) a single step using solutions. Reproduced (with modifications) with permission.^[^ ^135^ ^]^ Copyright 2000, Materials Research Society.

Advanced materials are often processed in two phases.^[^ ^135^ ^]^ A) the synthesis of materials (semiconductors, metals, organics), which is characterized by 1) a targeted chemical composition, 2) desired crystal structure, and 3) specific properties; and B) material fabrication (i.e., shape‐formation and shape fixation by firing/sintering, pyrolysis, melting, or casting) as shown in **Figure** **6** (right side). These processes are environmentally and energy stressing. In this regard, it is very difficult to give desired geometry and dimension to inorganic, due to their brittleness. Many high‐demand semiconductors undergo stress during shape formation and cracks may generate. The “classical” two‐step processing method usually demands high temperatures and consumes a lot of energy. However, by soft solution processing (SSP), one can fabricate specifically shaped, sized, reacted, and/or oriented materials, in situ only one step (left side of Figure 6). SSP can be regarded as environmentally friendly processing using solutions (preferably aqueous solutions). Hydrothermal/solvothermal, microwave‐assisted, and solution‐based synthesis belong to this category. It may yield similar results to every other process using fluids (such as vapor, gas, and plasma) or beam/vacuum processing, utilizing less overall energy than other processing routes. At the same temperature, it takes more energy to make melts and vapor than it does to form aqueous solutions. In thermoelectrics, SSP methods are preferable because the nanophase materials from these methods are raw materials for 2D and 3D TE devices printing technologies.

Green chemistry is a strategy to maximize efficiency and minimize harmful effects on the environment and human health. At present, it is very difficult to envisage a perfectly green chemical reaction. The 12 principles of green chemistry are to guide scientists in achieving the goals of preventing environmental contamination, protecting human health, and spending less energy.^[^ ^136^ ^]^ These principles rely on the minimization/non‐use of toxic solvents in chemical processes and analyses, as well as the non‐generation of wastes from these processes. Putting 12 principles of green chemistry into practice wherever feasible, whether in industry or in a research facility, can help to mitigate the environmental danger.

Although green chemistry principles are practiced in chemical and industrial manufacturing, until recently, academia has put less emphasis on ecologically friendly approaches in favor of obtaining specific target compounds.^[^ ^137^ ^]^ In academic research, typically, hundreds of milligrams to several grams of sample materials are synthesized in small laboratory batches, which is suitable for scientific study. In a typical laboratory, of the total chemical waste produced, 80% contains hazardous organic solvents.^[^ ^138^ ^]^ Chemical purification is usually a major source of solvent waste. Thus, achieving a greener chemical synthesis using less hazardous organic solvents is inevitable. Waste prevention at the source is necessary to develop cleaner processes and products.

#### 2.3.1 Hydrothermal (HT)/Solvothermal (ST) Synthesis

Among the low‐temperature synthesis methods, the HT method has a well‐established niche in nanophase materials synthesis. Byrappa and Yoshimura define HT as any heterogeneous chemical reaction in the presence of a solvent (an aqueous or non‐aqueous) above the ambient temperature, and at pressure > 1 atm in a closed system.^[^ ^139^ ^]^ Chemists prefer the term solvothermal, which refers to any chemical reaction that takes place in the presence of a non‐aqueous solvent or solvent in supercritical or near to supercritical conditions. Akin to ST and HT there are several other terms like glycothermal (in presence of glycol), alcothermal (in presence of alcohol), and ammonothermal (in presence of ammonia). However, supercritical fluid technology is considered as an extension of the HT technique. Therefore, the term hydrothermal is being used in many pieces of literature to describe all the heterogeneous chemical reactions taking place in a closed system in the presence of a solvent, be it aqueous or nonaqueous solutions.^[^ ^140^ ^]^ Compared to solid‐state reactions, which are usually carried out at elevated temperatures (above 700 °C) for several hours to several days, ST material processing requires low energy, shorter reaction times and most importantly scalable. The starting materials or precursors used in most ST syntheses of inorganic materials are metal salts and suitable solvents. Additionally, by changing the synthesis parameters: temperature, pressure, or reactant concentration, the properties of the particles can easily be controlled.

**Figure 7:**
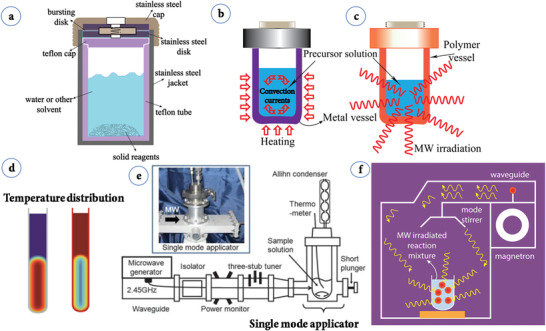
a) A Teflon‐lined, stainless autoclave typically used in the laboratory to perform subcritical ST synthesis; b) a general purpose HT/ST autoclave showing heat penetration; c) sample holder in microwave‐assisted HT/ST synthesis showing heat penetration; d) temperature distribution in general purpose and microwave assisted HT/ST synthesis. Reproduced with permission.^[^ ^144^ ^]^ Copyright 2004, Wiley‐VCH.e) Single mode applicator. Reproduced with permission.^[^ ^145^ ^]^ Copyright 2010, RSC. f) Multimode microwave applicator in operation.

Water is the most environmentally friendly and abundant solvent present in nature. Water behaves differently under HT conditions than it does under standard conditions. Water is a polar solvent and its polarity can be controlled by temperature and pressure, which is an advantage over other solvents. In HT synthesis, temperatures are typically in the range of 100 °C to 1000 °C and the pressure involved is large, 1 atmosphere to several thousand atmospheres. Conventional‐HT experiments are carried out using autoclaves with Teflon‐lined container (**Figure** **7**a). The majority of HT experiments are carried out below the supercritical temperature of water (374 °C). The primary advantages of the HT method are a) kinetics of reaction are greatly increased with a small increase in temperature, b) usually single crystals are obtained, c) new metastable products can be formed, d) high purity products can be obtained from impure feedstocks, e) the process is cost‐effective (no precipitants are needed in many cases), f) environmentally safe because of the closed system conditions, g) reagents can be recycled, h) some materials that cannot be prepared by other methods can be prepared (e.g., hydroxylated clays and zeolite molecular sieves).^[^ ^141^ ^]^ Furthermore, the low‐temperature HT reactions also avoid other problems related to stress‐induced defects (e.g., microcracks) encountered with high‐temperature melt processes.^[^ ^142^ ^]^ The first factor to consider when selecting an organic molecule as a solvent in ST chemistry is its role in the synthesis. The classification of organic solvents is typically based on the macroscopic and microscopic molecular parameters and empirical solvent polar parameters of compounds: molecular weight (*M*_r_), density (*d*), melting point (*M*_p_), boiling point (*B*_p_), molecular volume, heat of evaporation, dielectric constant (*ε*), dipole moment (*μ*_d_), and solvent polarity *E* ^T^_N_. Among these, the essential parameter to characterize the solvation property of a solvent is *E* ^T^_N_, which is defined as the total of the interactions between the solvent and the solute, including Coulomb force, dispersion force, induction force, H‐bond, and charge transport force. Table S1 (Supporting Information) lists the parameters of commonly used solvents in ST synthesis.^[^ ^143^ ^]^

HT/ST syntheses and their applications are not confined to thermoelectrics. This synthesis technique has been successful for the preparation of important solids: chemical sensors,^[^ ^146^ ^]^ luminescence phosphors,^[^ ^147^ ^]^ microporous crystals,^[^ ^148^ ^]^ superionic conductors,^[^ ^149^ ^]^ electronically conducting solids,^[^ ^150^ ^]^ complex oxide ceramics and fluorides,^[^ ^151^ ^]^ and magnetic materials.^[^ ^152^ ^]^ Although HT/ST techniques can provide excellent control over the morphology and particle size distribution, tuning stoichiometry and obtaining dense materials can be difficult, which can adversely affect the electrical transport properties of the materials. Furthermore, oxide and residual solvent could be removed with an effective post‐treatment process. However, post‐treatment may require a large amount of solvent. For the purpose of safety, it is necessary to have prior knowledge of the maximum pressure that may be created in a sealed vessel or it should be monitored regularly.

#### 2.3.2 Microwave‐Assisted Hydrothermal (MW‐HT) and Microwave‐Assisted Solvothermal (MW‐ST) Synthesis

To increase the kinetics of crystallization, one can instill microwave (MW) radiation or electric or ultrasonic fields into the HT environment and these combinations are termed microwave hydrothermal,^[^ ^153^ ^]^ (Figure 7c) electrochemical hydrothermal,^[^ ^154^ ^]^ and ultrasonic hydrothermal^[^ ^155^ ^]^ methods respectively. MW heating was initially used in chemical research in 1971, resulting in a slew of papers in the years that followed. The usage of a domestic MW oven in chemical synthesis was demonstrated successfully in 1986.^[^ ^156^ ^]^ In MW heating, MW radiation interacts directly with the reaction components (Figure 7c), so the sample alone is heated with less energy. In recent years, MW applicators built primarily for laboratory operations (Figure 7e) have made MW heating more accessible to novice users and the employment of MW techniques more common. Figure 7e shows the single‐mode MW applicator where a beam of MWs impinges on the sample holder, delivering energy; in a multi‐mode applicator, generated MWs are reverberated inside the cavity of the sample holder, thus transferring the energy to the reactants from all directions Figure 7f. The usage of household MW‐ovens in chemical synthesis is not uncommon. However, these ovens are not well equipped to measure the temperature, power, and exposure time very accurately. Section 2.3.3 will go over the design guidelines for the MW‐cavity (waveguide).

**Figure 8:**
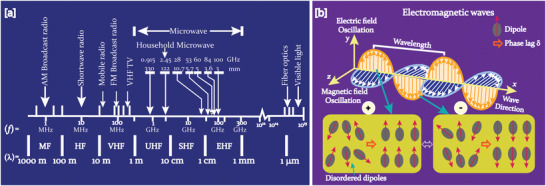
a) Microwave region in electromagnetic spectrum; b) heating mechanism of dielectric insulator materials. The agitation of the molecular dipoles is caused by the fluctuating electric field.

MWs occupy the part of the electromagnetic spectrum from 300 MHz (3 × 10^8^ cycles s^−1^) to 300 GHz (3 × 10^11^ cycles s^−1^) (**Figure** **8**a). MW photons with frequencies of 0.3, 2.45, and 30 GHz have quantum energies of 1.2 × 10^–6^ eV, 1.0 × 10^–5^ eV, and 1.2 × 10^–3^ eV respectively, which are within the range of energies separating the quantum states of molecular rotation and torsion. Since these energies are a million times lower than those of X‐rays, they cannot produce ionization or break chemical bonds where the quantum energy is at least a thousand times bigger.^[^ ^157^ ^]^ But they can only induce molecular rotations^[^ ^144^ ^]^(Figure 8b). Thus, unlike photochemistry methods, MW chemistry is based on the effective heat absorption properties of materials, rather than initiating chemical reactions by direct absorption of electromagnetic energy. The 915 MHz, 2.45 GHz, 5.8 GHz, and 24.124 GHz are common frequencies used for materials processing.^[^ ^158^ ^]^ Among these, 2.45 GHz (wavelength of about 12.24 cm) is commonly used in laboratories and household MW appliances.

MW irradiation generates heating in materials or liquid via two basic mechanisms: dipolar polarization and ionic conduction. While the dipoles in the reaction mixture (polar solvent molecules or reagents) are engaged in the dipolar polarization effect, the charged particles (usually ions) are involved in ionic conduction. In the presence of MW frequencies, the dipoles/molecules reorient themselves in order to be in phase with the oscillating *E*‐field (Figure 8b). Since the *E*‐field component is oscillating, in the absence of an *E*‐field, dipoles try to come back to a random state and this does not happen immediately. The time required for molecules from aligned states in presence of *E*‐field to random states (or equilibrium state) in transient absence of *E*‐field is called relaxation time (*τ*). Inertial, elastic, frictional, and molecular interaction forces hinder these frequent changes in orientations of dipoles/molecules which increase molecular kinetic energy and result in volumetric heating. MW energy loss via this dipole/molecular friction process is called dipolar loss^[^ ^159^ ^]^ and the process of heating using the electric field (*E*‐field) component of the high‐frequency MW radiation is referred to as dielectric heating. The amount of heat generated through this process is directly related to the ability of the dipoles to align themselves with the frequency of the applied field and this depends on the functional groups and volume of the molecule. There is no heat generated if the molecular dipoles do not reorient with the applied field (high‐frequency irradiation) or reorient very quickly (low‐frequency irradiation). If the *E*‐field component is oscillating at 10^12^ times s^−1^, even the smallest molecules are no longer able to rotate significantly. The assigned frequency of 2.45 GHz, which is used in all commercial systems, is halfway between these two extremes, allowing molecular dipoles to align in the field but not perfectly following the alternating field. Similarly, under the influence of the MW field, the dissolved charged particles in a reaction medium (typically ions) oscillate back and forth. In this process, they tumble and knock their neighboring molecules or atoms. These collisions cause motion and generate heat. When compared to the dipolar rotation mechanism, the ion conductivity channels have a substantially higher heat‐generating capability.^[^ ^144^, ^160^ ^]^

When a dielectric material is exposed to MW energy, part of this incident energy is absorbed by the dielectric molecules and their movement increases, generating heat as described above, and this process is called dielectric loss. The capability of a material or solvent to convert MW energy into heat at a particular frequency and temperature is determined by the loss tangent (also called energy dissipation factor in some literature) and given by

(5) $$\tan\delta =\frac{\varepsilon^{\prime \prime }}{\varepsilon^{\prime }}$$

where *δ = *90° – *ϕ*, is the phase lag or loss angle between the applied electric field and materials polarization, *ε*′′ is the dielectric loss factor (indicative of the efficiency with which the electromagnetic radiation is converted into heat) and *ε*′ is the relative permittivity (described by the polarizability of molecules in the electric field).^[^ ^160^ ^]^ In general, at the standard operating frequency of a MW reactor (2.45 GHz), a reaction medium with a high tan*δ* is required for good absorption and, consequently, efficient heating.^[^ ^160^ ^]^ In simple words, the tan*δ* of the solvents is related to the ability of the solvent to absorb MW energy. The tan*δ* depends on the relaxation time (*τ*) of the molecule. The *τ*, in turn, is highly dependent on both the kind of functional groups (particularly those capable of hydrogen bonding) and the molecule volume.^[^ ^159a^ ^]^ Overall, MW solvents can be classified as high‐ (tan*δ *> 0.5), medium‐ (tan*δ* = 0.1–0.5), and low‐absorbing (tan*δ *< 0.1).^[^ ^144^ ^]^ Materials with a low value of tan*δ* are nearly transparent to MWs (at that frequency) and will not undergo appreciable heating. In the case of mixtures of materials, selective heating happens based on the relative dielectric properties of the individual constituents.^[^ ^161^ ^]^

The parameter, tan*δ* is a helpful parameter when it comes to comparing the heating rates of a series of solvents/compounds with similar physical and chemical properties. However, the tan*δ* does not provide a complete picture of the methodology of choosing a solvent for a particular nanomaterial synthesis with good rate enhancement, high product yield, high product purity, and high reproducibility. The MW heating rate of p‐nitrophenetole dissolved in liquid naphthalene (*η *≈ 0.75 cP) is found to be greater than that of p‐nitrophenetole dissolved in liquid mesitylene (nonabsorbing solvent with *η *≈ 0.52 cP), implying that the dielectric loss in p‐nitrophenetole/naphthalene is greater.^[^ ^162^ ^]^ Where η is the viscocity. The heating curves (at 50 W applied MW power) for a series of structurally similar aromatic dipolar molecules: nitrobenzene, nitroanisole, and nitrophenetole (all 0.1 m) mixed with a nonabsorbing mesitylene solvent show that the heating rate for nitrophenetole (*μ*_d_ = 5.81 D) is higher than nitrobenzene (*μ*_d_ = 4.39 D), and nitroanisol (*μ*_d_ = 5.61 D) in between.^[^ ^162^ ^]^ The contribution for *τ*s may manifest from the entire molecule or a functional group adhered to a large molecule, and it depends on the intermolecular forces between the molecules as well as the size of the molecule. Table S3 (Supporting Information) lists the *τ*, *η*, and tan*δ* values for different solvents.^[^ ^159^, ^161^ ^]^

##### Microwave Absorption and Penetration Depth in Liquids

The tan*δ * is a function of temperature and MW frequency, and these latter two parameters are strongly connected to the penetration depth *D*_P_, defined as the depth in the solvent where the incident/incident irradiated MW power is attenuated to 37% (i.e., 1/e) of its initial/incident value.^[^ ^163^ ^]^ The microwave *D*_P_ is inversely proportional to the tan*δ*. The solvents and materials with high tan*δ* have shallow *D*_P_. Water, which is routinely used as a solvent, has a *D*_P_ of ≈a few centimeters at RT, which has far‐ranging implications for the scale‐up of MW synthesis. For pure water and majority of organic solvents, the tan*δ* decreases with the temperature. Thus, MW heating of water to a high temperature is somewhat difficult unless it is mixed with another solvent with high tan*δ*.

**Figure 9:**
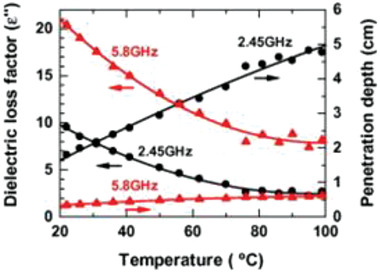
Variation of dielectric loss factor (*ε*′′)and penetration depth with temperature at 2.45 and 5.8 GHz MW in water. Reproduced with permission.^[^ ^164^ ^]^ Copyright 2009, Elsevier.

A study on the variation of dielectric loss factors (*ε*′′) of pure water with temperature shows that when the water temperature increases from 22 °C to 99 °C, the dielectric loss factors are decreased by ≈72% and ≈60% for MW frequency of 2.45 GHz and 5.8 GHz respectively.^[^ ^164^ ^]^ For the same temperature range, the *D*_P_ of the 2.45 GHz MW in water increases from ≈1.8 to 4.8 cm, whereas the *D*_P_ is rather shallow for the 5.8 GHz MWs (from 0.34 to 0.62 cm) as shown in **Figure** **9**. The *D*_P_s in oleylamine are found to be 22.8 and 3.8 cm for 2.45 and 5.8 GHz MW, respectively.^[^ ^165^ ^]^ For low‐loss dielectric material (i.e., $\frac{\delta^{\prime \prime }}{\delta^{\prime }}$ << 1), the depth (*D*_P_, in cm) can be estimated from Equation 6.

(6) $$D_{p}=\left(\frac{\lambda_{o}}{2\pi }\right)\left(\frac{\sqrt{\varepsilon^{\prime }}}{\varepsilon^{\prime \prime }}\right)$$

where *λ*_o_ is the wavelength of the incident MW radiation (*λ*_o_ = 0.122 m at 2.45 GHz; and *λ*_o_ = 0.0517 m at 5.8 GHz). *D*_P_ denotes the depth at which the MW power density is reduced to 1/e of its incident value. 2.45 GHz MW are commonly used in laboratories, but since the *D*_P_ depends on the frequency, variable‐frequency MWs heating can also be performed where a selected band of MWs is used to sweep the sample (around a central frequency) for a specified time.

If no suitable MW absorption reagents are available for the synthesis of a material, another substance known as a susceptor that can function as a heat source can be employed. A susceptor is a material with a high dielectric tan*δ*. This material is capable of absorbing electromagnetic energy and converting it to heat. A susceptor can be in direct contact with the sample. For example, adding a highly MW absorbing solute molecule to a MW nonabsorbing solvent; then the heat acquired by the entire solution comes from the convective heating of the solvent medium by the MW‐heat absorbing solute alone. Or, a sample can be kept separately by enveloping the reaction vessel (or test tube) in a container of the susceptor material. Such cases will be discussed in Section 2.3.4 and Section 2.3.8. Commonly used susceptors are carbon (graphite or amorphous carbon), SiC, and copper (II) oxide.^[^ ^166^ ^]^ Usage of susceptor can cause problems. For example, reactions in which susceptors must be combined with reagents might result in product contamination (or even undesired side reactions), requiring an extra separation step in the synthesis process.

##### Solvents Used in Microwave Liquid‐Phase Syntheses

As we already mentioned, the tan*δ* of the solvents depends on the relaxation times (*τ*) of the molecules (time required from aligned states in presence of *E*‐field to random states in the transient absence of *E*‐field). These *τ* depend essentially on the nature of the functional groups and the volume of the molecule.^[^ ^159a^ ^]^ The use of excellent MW absorbing solvents results in high heating rates. Table S2 (Supporting Information) shows loss tan*δ* (for 2.45 GHz and 20 °C) and BP of some commonly used solvents. The next section discusses the significance of various types of solvents employed in MW‐assisted nanostructure preparation.

*Water*: Water is environmentally friendly, noncorrosive, nonflammable, and has a lower vapor pressure than organic solvents;^[^ ^167^ ^]^ MW‐assisted synthesis of inorganic nanostructures in an aqueous solution has garnered a lot of interest. For water, at 20 °C, the value of the relaxation peak frequency is ≈18 GHz and *ε*′′is quite significant at 2.45 GHz, making water the universal solvent in MW‐assisted synthesis. When ionic salts are dissolved in water, the *τ* of the mixture decreases at low concentrations and then increases. It has been proposed that the presence of ions in water triggers a structure‐breaking process. Water molecules that are coordinated with the ions are rotationally locked, while those that are not do not have as strong intermolecular hydrogen bonding effects and, as a result, have lower *τ*s in this free‐state.^[^ ^159a^ ^]^ Because of a stronger ordering of the water molecules when a large number of ions are present, the effects are reversed at higher concentrations, and the *τ *of water in concentrated salt solutions is higher than that of pure water. The temperature range of water in liquid state is narrow (0–100 °C) and many organic compounds have low solubilities in water. These drawbacks limit the use of water as a solvent in a wide range of material synthesis applications.

*Polyalcohols*: Polyalcohols: ethylene glycol (EG), 1,3‐propanediol, 1,4‐butanediol, and glycerol (C_3_H_8_O_3_), have high tan*δ*s (Table S2, Supporting Information) and therefore, they form a family of strongly MW absorbing solvents and used in MW‐assisted synthesis of inorganic nanostructures. The most commonly used polyalcohol is EG due to its much higher dielectric loss, higher BP, and relaxation time ≈112.87 ps as compared to water and it is reductive in nature. The heating rate and temperature that may be attained in an open reaction system using EG as the solvent are even higher than in certain HT/ST processes.

*Ionic Liquids (IL)*: At both low and high temperatures, ILs are good MW absorbers. They are nonflammable, nonvolatile, and thermally stable organic salts, described as “green solvents” to replace conventional organic solvents in many chemical processes due to their exceptional features: low melting point <100 °C, relatively low viscosity, low vapor pressure, low interfacial tension, high ionic conductivity, and adjustable solvent polarity.^[^ ^168^ ^]^ Moreover, ILs endowed with superior dissolution capability and stabilization of metal cations, which make them good solvents and potential capping agents, or surfactants in inorganic synthesis. ILs are excellent MW absorbers because of their ionic character and high polarizability. There are two mechanisms that contribute to energy absorption and hence dielectric heating in an aqueous solution of ions. These are ionic drift (that give rise to Joule heating) and dipolar relaxation due to the relaxation of the water molecules. As the concentration of ions increases, ionic contribution to dielectric heating predominates over dipolar relaxation. Consequently, the value of *ε*″ increases with decreasing frequency. That means the introduction of ions into a solution markedly increases the dielectric heating rates. The tan*δ* of IL, 1‐butyl‐3‐methylimidazolium hexafluorophosphate ([bmim][PF_6_]) increases rapidly from 0.185 (20 °C) to 1.804 (100 °C) and 3.592 (200 °C).^[^ ^169^ ^]^ Therefore, its ability to absorb MWs increases with increasing temperature due to ionic conduction.^[^ ^161^ ^]^

Phosphonium ILs offer various benefits over other types of ILs, including great thermal stability, low viscosity, and good reaction stability in strongly basic or strongly reducing conditions.^[^ ^170^ ^]^ Phosphonium ILs, tetrabutylphosphonium halides are very suitable to dissolve S, Se, and Te at elevated temperatures.^[^ ^171^ ^]^ The dissolved chalcogens can easily react with various elemental metal powders under MW heating to produce micro/nanostructured metal chalcogenides. Another advantage is the weak binding strength of the phosphonium ILs allows nearly complete removal of organic remnants by simple washing.

*Alcohols*: As shown in Table S3 (Supporting Information), alcohols are ideal solvents for MW heating because their ability to absorb MWs is typically greater than that of water. Alcohols can form H bonds in the same way that water can, albeit to a lesser extent, and thus, their dielectric properties are very similar to those of water. Furthermore, the dipole moments of aliphatic alcohols are comparable to those of water. Also, as the chain length of the alcohols increases the *τ* becomes longer.

Table S3 (Supporting Information) lists relaxation times (at 20 °C), dipole moment, and dielectric properties of aliphatic alcohols. Listed in table are properties of water for comparison. The standard MW frequency for dielectric heating (i.e., 2.45 GHz) corresponds to a *τ* of 65 ps. Therefore, those compounds that have *τ* ≈ 51.5–800 ps, easily couple MW of this frequency. The RT tan*δ *of ethanol is 0.941 (at 20 °C; 2.45 GHz) >> 0.123, for water. Therefore, ethanol is an excellent MW absorbent.^[^ ^159a^ ^]^ As suitable solvents alcohols have been used in MW‐assisted preparation of a variety of nanocomposites. Such case studies are discussed in the synthesis section.

*Mixed Solvents*: Mixed solvents, in addition to single‐solvent MW‐assisted synthesis, are widely utilized in MW‐assisted synthesis to provide additional control over the formation of nanostructures. In mixed solvent reaction systems, different solvents can be chosen and their volume ratios can be altered, allowing for greater control of the chemical composition, structure, size, and morphology of the final product by regulating the experimental parameters.

A nonpolar solvent can be made into a MW absorbing solvent by adding a small amount of MW absorber ionic liquid to it. Leadbeater and Torenius have shown that by adding a small amount of 1,3‐dialkylimidazolium iodide to hexane, the temperature can reach 217 °C after MW heating at 200 W for 10 s.^[^ ^172^ ^]^ In the absence of an ionic liquid, the temperature was only 46 °C after MW heating at 200 W for 10 s. Adding a small amount of ionic liquids (1,3‐dialkylimidazolium‐type) as a cosolvents to MW‐inactive solvents: toluene and cyclohexane significantly reduced the heating time of the parent solvent.^[^ ^173^ ^]^ Similarly, when polar solvent (with high tan*δ*) is mixed with nonpolar solvent, heating rates for the whole mixture increase as in the case of CHCl_3_–CCl_4_ mixture.^[^ ^159a^ ^]^ In another scenario involving polar (water) and nonpolar (chloroform) solvent mixtures, the water phase can reach 100 °C while the chloroform phase can maintain a low temperature (below its BP 61 °C), allowing the reactants to be extracted from one phase to the other.^[^ ^174^ ^]^

If the liquids are chemically similar and molecularly homogeneously mixed, the combination will frequently exhibit a single relaxation process at an average point. However, if the solvents are molecularly immiscible (e.g., alcohols and bromides and alcohols and ethers), two distinct *τ* are observed, which are not significantly different from those of pure solvents.

*Water/Polyols*: Polyols have a number of OH functional groups that are connected to the carbon backbone. Because of their high tan*δ* (compared to water) they are suitable for MW heating. Polyols are extremely miscible with water, and their molecules may establish intermolecular hydrogen bonds with one another and with water molecules in solution. The polyol compounds usually have high viscosities (which is reflected in long *τ*) due to strong hydrogen bonding.^[^ ^159a^ ^]^ The combination of water and polyols will almost surely boost MW absorption when compared to utilizing water as the only solvent. Furthermore, because some reactants have limited solubility in polyols, adding water to polyols significantly increases the solubility of many reactants.

*Water/Alcohols*: Given that ethanol has a substantially greater tan*δ* (0.941 at 2.45 GHz and 20 °C) than water (0.123 at 2.45 GHz and 20 °C), combined solvents of water and alcohols would surely enhance the capacity to absorb MWs when compared to utilizing water as the only solvent. Open reaction systems with mixed solvents of water and alcohols will only allow low‐temperature chemical reactions because alcohols (with low molecular weights) often have low BPs. As a result, closed‐system ST reactions are required for relatively high‐temperature MW‐assisted synthesis.

*Microwave‐Assisted Reactions in Open and Closed Systems*: The MW‐assisted synthesis of inorganic materials in liquid phase is broadly classified into two categories: open reaction systems and closed reaction systems. In open reaction system or methods, under the atmospheric pressure, the BP of the solvents regulates the reaction temperature. This method is safe, cost effective, and easy to implement as the precursors and solvents are heated in an open MW transparent glass flask or Teflon tube. Polyols with high BP: EG (BP ≈198 °C, tan*δ* = 1.350) and glycel (C_6_H_17_N_2_O_5_P, BP ≈ 290 °C) are commonly employed as typical solvents in MW‐assisted preparation in an open reaction system. Similarly, many ionic liquids with high and safe BPs are used in open reaction systems. Using EG as the solvent, the reaction temperature can be set anywhere between RT and ≈198 °C. Glycel is another polyol solvent and its temperature can be varied between RT and ≈290 °C in the open reaction systems. Low BP of methanol limits the reaction temperature to a maximum of 65 °C. Therefore, such solvents are unsuitable for many high‐temperature chemical reactions. In closed MW‐assisted reaction systems (similar to HT/ST), the temperature of solvents can be raised considerably well beyond their BPs by increasing autogenous pressure resulting from heating. For example, EtOH has a BP of 78 °C, and MW heating in a closed vessel may quickly raise its temperature to 164 °C (at a pressure of 12 atm), resulting in a thousand‐fold increase in reaction rate.^[^ ^159a^ ^]^

#### 2.3.3 Microwave Waveguide Design Basic Principle

MWs also have a magnetic field (*H*‐field) component, which can couple with some materials to induce heating. For example, aqueous electrolyte solutions such as NaCl, CaCl_2_, KCl, NaBr, and NaBF_4,_^[^ ^175^ ^]^ and a broad range of materials, including conductors, semiconductors, magnetic materials, and so on.^[^ ^176^ ^]^ Therefore, the MW cavity should be designed in such a way that the user can access *E*‐field or *H*‐field on the same device for both liquid phase and solid phase synthesis or characterization.

**Figure 10:**
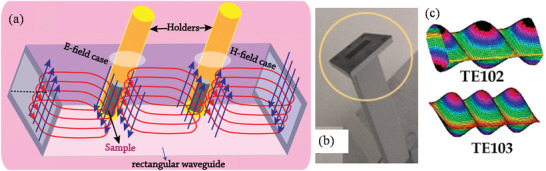
a) Rectangular waveguides made of high‐conductivity metals (copper, silver, gold, and brass) that support various patterns of *E* and *H* modes propagating along the guides; b) view of the open end of a waveguide built with a flange to connect with other waveguides. Adapted with permission.^[^ ^158^ ^]^ Copyright 2018, In Tech. c) TE_102_ (*H*‐field) or TE_103_ (*E*‐field) resonant modes. Adapted with permission.^[^ ^177^ ^]^ Copyright 2015, MDPI.

From electrodynamics, it is well known that very good conductors, particularly metals, reflect electromagnetic waves with minimal losses, and that the skin depth of these conductors is very small (fraction of microns to microns). Therefore, multiple reflections inside hollow metallic pipes with mirror‐polished walls are the preferred choice to deliver MWs from one point to another. Once we know the patterns of electromagnetic waves inside the metallic waveguide (maxima and minima of *E* and *H*‐field), we can place the material of interest at the locations where the *E*‐field or *H*‐field dominates (**Figure** **10**a). In a single‐mode apparatus, by adjusting the length of the cavity, it is possible to work with TE_102_ or TE_103_ resonant modes (Figure 10c). When TE_102_ mode is sets in, the sample is placed at the amplitude maximum of *H*‐field; and in TE_103_ mode, the sample is placed at the amplitude maximum of *E*‐field.^[^ ^178^ ^]^ The sample temperature can be monitored with a pyrometer. To insert the specimen at the intended spot, windows will be carved. Depending on the positions of the windows (*E*‐field or *H*‐field), the material will interact with the *E* or the *H*‐field component.

*Summary*: MW‐ST chemical reactions and processes clearly have advantages as opposed to the conventional ST reaction: rapid and uniform heating, energy savings process, shorter preparation time, higher yield, lower processing cost, narrow particle size distribution, and high purity over other conventional approaches. However, some challenges need to be addressed when it comes to the usage of domestic MW ovens:

1) Some practical experiences on relevant parameters are required. Many publications do not provide essential reaction details: max. power, type of MW irradiation (pulsed or continuous), etc. which results in poor reproducibility. In many cases, the power used is described in terms of full power or the preset power is given. 2) The setting of reaction parameters is only confined to energy input and the irradiation time. Accurately measuring temperature and chemical reaction pressure is exceedingly difficult. 3) Controllable synthesis of complex systems through the MW‐assisted method has proven to be a difficult task.^[^ ^179^ ^]^ 4) In domestic MW ovens, power peaks might force the formation of hotspots triggering the risk of spontaneous noncontrollable exotherms. To remedy these shortcomings, in recent years, MW applicators designed specifically for laboratory operations (Figure 7e). However, some problems still persist. As opposite to conventional convective heating, at present MW based synthesis technique still lacks qualities such as the degree of flexibility, the level of refinement in nanostructure size, composition, shape, and crystal structure. Another significant problem is the variation of dielectric properties with temperature for most solvents. For example, at RT, ethanol is a rather strong MW absorber with tan*δ *= 0.941. At 100 °C and 200 °C, tan*δ* of ethanol drops to 0.270 and 0.080 respectively.^[^ ^161^ ^]^ In contrast, in ionic liquids, where heat is generated by ionic conduction, the ability of a material to absorb MW increases with temperature.^[^ ^159a^ ^]^ Because ionic liquids are particularly effective MW absorbers at higher temperatures, precise temperature monitoring, reaction control, and heating are challenging.^[^ ^180^ ^]^ The conventional HT/ST method and the MW‐assisted hydro/ST synthesis methods differ in that the conventional HT/ST method requires a long period of time (typically one to several days) to complete the reaction. Contrary, in MW‐assisted methods, the preparation time can be drastically reduced to minutes instead of days.

#### 2.3.4 Hydrothermal/Solvothermal and MW Liquid Phase Synthesis of TE Materials

In recent years, HT, ST, and MW‐assisted HT or ST approaches for the fast synthesis of inorganic TE nanostructures have been extensively explored, and multiple studies on such methods have been published. Using various solvents and mixed solvents, a variety of nanostructures of various TE materials were prepared. As a result, a full examination of these methodologies is critical for the future rapid development of the relevant research discipline. In the subsequent section, the recent progress in HT/ST and liquid‐phase MW‐assisted synthesis of TE nanostructures in various types of solvents will be reviewed and discussed on the basis of the classification of different kinds of materials.

##### (Bi,Sb)_2_(Te,Se)_3_ Compounds

**Figure 11:**
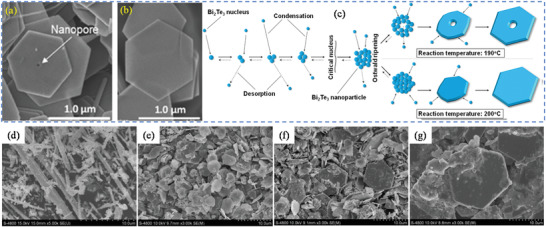
SEM images of the Bi_2_Te_3_ NPts synthesized at a) 190 °C and b) 200 °C; c) crystal growth of NPts with and without single nanopores. Reproduced with permission.^[^ ^183^ ^]^ Copyright 2019, Springer Nature. SEM images of Sb_2_Te_3_ NPts synthesized with different concentrations of NaOH: d) without NaOH; e) 0.1; f) 1.0, and g) 1.4 m. Reproduced with permission.^[^ ^184^ ^]^ Copyright 2019, Elsevier.

Nanosized Bi_2_Te_3_ materials of various shapes and sizes have been reported including nanoplates,^[^ ^181^ ^]^ nanoparticles,^[^ ^182^ ^]^ and nanocrystalline films.^[^ ^130b^ ^]^ Recently, 2D Bi_2_Te_3_ hexagonal nanoplates (NPt) with and without nanopores have been prepared through ST synthesis.^[^ ^183^ ^]^ Bi_2_O_3_ and TeO_2_ were used as Bi_2_Te_3_ sources, and C_2_H_6_O_2_ was used as a ligand. NaOH and polyvinylpyrrolidone were also used in the solution. The reaction was carried at either 190 °C or 200 °C for 4 h. At a reaction temperature of 190 °C for 4 h, the obtained hexagonal NPts had edge lengths of ≈1 µm and a single nanopore (20 nm in diameter) appeared at the center of the NPts as shown in **Figure** **11**a. At a reaction temperature of 200 °C for 4 h, the NPts had a similar edge length of ≈1 µm but no nanopores were present (Figure 11b). Similarly, the NPts obtained at a reaction temperature of 180 °C had nanopores, but not those obtained at a reaction temperature of 230 °C. The composition ratio Te/(Bi + Te) of the NPts with no nanopores (0.54) was less than that of the NPts with nanopores (0.56), thus giving credence to the presumption that a large number of Te atoms had replaced Bi atoms in the NPts without nanopores. At a reaction temperature of 190 °C, Ostwald ripening (dissolution of smaller particles) resulted in the formation of nanopores due to lower crystallinity or less dense particles in the colloidal aggregate, which gradually dissolved, whereas larger, well crystallized or denser particles in the same aggregate continued to grow at the lower reaction temperature. At 200 °C, nanopores were not formed because the particles in the colloidal aggregate did not dissolve owing to the dense structures which were formed at the higher reaction temperature initially.

During synthesis, the structure, morphology, and size of the Sb_2_Te_3_ nanostructures can be affected by many factors: the amount of ligand, reaction pH, and precursor species employed in the synthesis. To decipher the effects of the synthesis conditions, Sb_2_Te_3_ hexagonal plates were synthesized under different NaOH concentrations (0, 0.1, 1.0, and 1.4 m) using a ST process (230 °C for 24 h) under various synthetic conditions, and their morphology, size and uniformity changes were investigated.^[^ ^184^ ^]^ The starting materials: SbCl_3_, K_2_TeO_3_, polyvinylpyrrolidone (PVP), DEG, and NaOH were used for the reaction. The end product was annealed at 623 °C for 6 h and consolidated by SPS. It was found that using Sb_2_O_3_ and TeO_3_ as Sb and Te precursors resulted in relatively small Sb_2_Te_3_ particles but with clear hexagonal shapes (4–5 µm), while Sb_2_Te_3_ plates synthesized using SbCl_3_ and Na_2_TeO_3_ were larger in size (≈8 µm) and thin. When the reaction was conducted without NaOH, the main product was Te nanorods and a small amount of Sb_2_Te_3_. When synthesized with NaOH, all Sb_2_Te_3_ nanoparticles were plate‐like. With a concentration of NaOH as low as 0.1 m, a small (≈2 µm) round plate‐like appearance (Figure 11e) was observed, in which the shape of the hexagonal plate was barely observable. With a further increase in NaOH amount, hexagonal plates were formed and the sizes increased to ≈10 µm (Figure 11f). However, when 1.4 m of NaOH was used, the plates were found to be aggregated, forming an assembly with indistinct shapes. In terms of particle size, higher NaOH concentration yielded larger particles (Figure 11g). The capping agent, PVP presumably played an important role in stabilizing the nucleus formed in the initial stages of the reaction, in addition to influencing the morphology of nanomaterials. Higher PVP concentrations yielded smaller NPts. At 300 K, the maximum *S* of the NPt sample was 350 µV K^−1^, and the *κ * was 0.5 W m^−1^ K^−1^.

An *n*‐type Bi_2_(Se*_x_* Te_1−_ *_x_*)_3_ NPts with enhanced TE properties were prepared via ST synthesis (200 °C for 20 h) by adjusting the composition of Se.^[^ ^185^ ^]^ Bi_2_O_3_, SeO_2_, TeO_2_, EG, PVP, and NaOH were used to prepare the precursor solution. At a Se composition of 0.08, regular‐hexagonal NPts (diameter ≈500 nm; thickness < 50 nm) were formed; when the Se composition was increased from 0.25 to 0.58, the edge length of the NPts increased and their shape gradually shifted away from the regular hexagon; when the Se composition was increased to 0.75, the plate size varied from 0.5 to 3 µm, and the plate shape became random. To measure the TE properties, NPt thin films were prepared via drop casting. With an increase in the Se composition, the *σ* of the NPt thin films increased, but drastically decreased at an extremely high Se composition because of the presence of the impurity phase, Bi_2_Se_2_Te. The highest *σ* of the thin film was 317 Ω cm^−1^ at *x* = 0.75. The highest *S* (−128.6 µV K^−1^) and the lowest *S* (−82.0 µV K^−1^) were observed for *x* = 0.58 and *x* = 0.83 respectively. Thus, a clear relationship between the Se composition and the *S* was not observed. Nanosheet thin film of Bi_2_(Se*_x_* Te_1−_ *_x_*)_3_ with *x* = 0.75, showed the highest *PF* of 4.1 µW cm^−1^ K^−2^. According to the proposed growth mechanism of the Bi_2_(Se*_x_* Te_1−_ *_x_*)_3_ NPts, when the Se composition is low, the Se atoms replaced the Te atoms at Te(2) sites. During this stage, the regular hexagonal shape of the NPts was maintained. As the Se atoms started to fully occupy the Te(2) sites, these atoms began to fill the Te(1) sites via disordered occupation of Te/Se atoms. As a result, the crystal growth of NPts became disordered, and the shape and size of the NPts became random. With further increase in the Se composition, the Bi_2_Se_2_Te phase was formed and the shape was very different from hexagonal.

**Figure 12:**
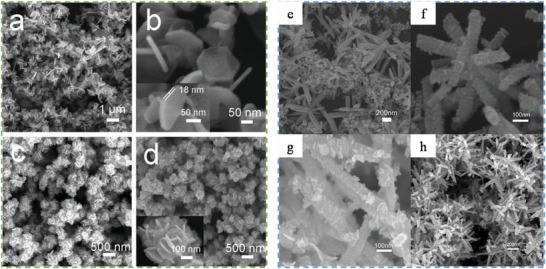
a) Low and b) high magnification FESEM images of Bi_2_Te_3_ plates; c) low d) high magnification FESEM images of Bi_2_Te_3_ flowers. The FESEM images of a single plate and flower were respectively inserted in (b) and (d). Reproduced with permission.^[^ ^186^ ^]^ Copyright 2012, RSC. Bi_2_Te_3_ 1D nanorod bundles. SEM image of samples obtained at different reaction times: e) 0 min; f) 30 min; g) 4 h, and h) 48 h. Reproduced with permission.^[^ ^187^ ^]^ Copyright 2013, Wiley‐VCH.

Bi_2_Te_3_ NPts (thickness of 15–20 nm) and self‐assembled flower‐like nanostructures using Bi_2_Te_3_ NPts as building blocks have been fabricated with a HT approach (180 °C for 48 h) with ethylenediaminetetraacetic acid (EDTA) as an additive.^[^ ^186^ ^]^ The reagents: bismuth nitrate (Bi(NO_3_)_3_), K_2_TeO_4_, hydrazine hydrate (N_2_H_4_.H_2_O), NH_3_∙H_2_O, ethylenediaminetetraacetic acid, tartaric acid (C_4_H_6_O_6_), C_6_H_8_O_7_, disodium ethylene diamine tetra acetic acid, ethylene diamine tetra acetic acid tetra sodium, NaBH_4_, NaOH were used in the synthesis process. With no residual additives present, the NPts and flower‐like nanocrystals (**Figure** **12**a–d) were consolidated by high pressure (673 K and 4 GPa) cubic anvil high‐pressure apparatus). The flower‐like *n*‐type Bi_2_Te_3_ nanostructures exhibited *κ*, *PF*, and *zT* of 0.5 W m^−1^ K^−1^, 8.6 µW cm^−1^ K^−2^, and 0.7 (at 453 K), respectively.

Self‐assembled Bi_2_Te_3_ 1D nanorod bundles (diameter of single bundle ≈ 700 nm) have been fabricated by a facile ST method (180 °C for 48 h) with EDTA as an additive and then sintered (673 K, 4 GPa, 10 min).^[^ ^187^ ^]^ The used reagents were Bi(NO_3_)_3_, K_2_TeO_4_(CP), N_2_H_4_·H_2_O, 1,2‐ethylenediamine, and ethylenediaminetetraacetic acid. The use of the EDTA molecule as a chelating agent played a key role in the formation of Bi_2_Te_3_ nanorod bundles. In this case, the reaction temperature strongly affected the morphology of the product. For example, at a lower temperature (120 °C, 140 °C), the as‐prepared samples consisted of numerous small particles. When the temperature was increased to 180 °C, well‐defined products were obtained without small particles (Figure 12e–h). It was found that a higher growth temperature (e.g., 180 °C) is very conducive for the formation of the Bi_2_Te_3_ structure with good crystallinity. The sintered sample showed a *κ* of 0.55 W m^−1^ K^−1^ at 313 K (with *κ*_l_ of ≈0.25 W m^−1^ K^−1^). The maximum *S* was ≈135 µV K^−1^ and the maximum *zT* was found to be 0.43 at 473 K.

In a HT synthesis (200 °C) the anionic surfactant: sodium bis(2‐ethylhexyl) sulfosuccinate (AOT) was used for the synthesis of single‐crystalline Sb_2_Te_3_ nanobelts with lengths in the range of several tens to several hundred µm, a width of 1–3 µm, and thickness of ≈100 nm.^[^ ^188^ ^]^ In the synthetic process, the AOT functioned as the shape controller. The reaction time and temperature had strong effects on the formation of the nanobelts. The growth of the Sb_2_Te_3_ crystal was along the *a*‐ or *b*‐axis. Shi et al. prepared impurity‐free Sb_2_Te_3_ Hexagonal NPts by HT treatment (180 °C for 5 h) without any organic additives or templates.^[^ ^189^ ^]^ Film of these NPts showed *p*‐type behavior and exhibited a high *S* of ≈125 µV K^−1^ at RT, which is higher than the *S* of Sb_2_Te_3_ bulk crystals. With a tan*δ* of 1.350 at 20 °C (for 2.45 GHz), EG is a very good MW absorbing solvent as well as an effective reducing agent, as the following conventional and MW‐ST studies show. In the polyol process,^[^ ^190^ ^]^ polyol serves as both the solvent and the reducing agent.

Bi_1_ *_−x_* Sb*_x_* (with *x* = 0, 0.12, and 1) alloy nanoparticles (5–50 nm range) have been synthesized by a ST route using *N,N*‐dimethylformamide and EG as solvent/reducing agent; BiCl_3_, SbCl_3,_ and Bi(NO_3_)_3_ as starting materials; and citric acid as a surface modifier/stabilizing agent.^[^ ^191^ ^]^ The size and shape of the nanoparticles were very different in the case of Bi and Sb. Using citric acid as the surface modifier, highly crystalline rhombohedral and spherical Bi nanoparticles could be obtained. Varying the solvent and quantity of citric acid resulted in different particle size distributions. Changing the precursor/citric acid ratio yielded a narrow particle size distribution (20–80 nm). Decreasing the concentration of reducing agent resulted in a much narrower size particle distribution (15 ± 7 nm). It was observed that Sb and Bi_0.88_Sb_0.12_ nanoparticles could also be produced by similar methods and reactants, but the morphology of the particles was more difficult to regulate compared to Bi nanoparticles.

**Figure 13:**
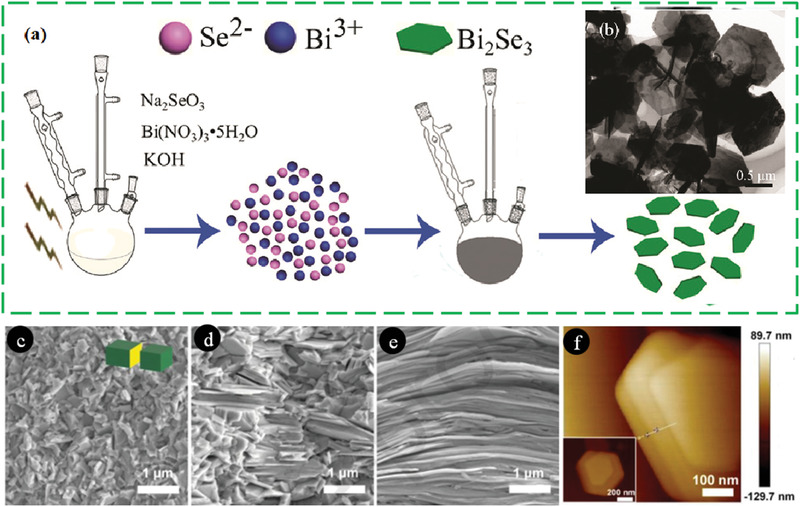
a) The suggested mechanism for the production of Bi_2_Se_3_ nanosheets; (B) low magnification SEM image of ultrathin Bi_2_Se_3_ nanosheets. Reproduced with permission.^[^ ^192^ ^]^ Copyright 2014, RSC. c–e) cross‐sectional SEM images of the textured samples; f) AFM image of step‐like Bi_2_Te_3_ NPts prepared with 10 m concentration of NaOH. Reproduced with permission.^[^ ^194^ ^]^ Copyright 2020, Elsevier.

A MW‐assisted approach has been used to fabricate ultrathin Bi_2_Se_3_ in the presence of EG at a reaction temperature of 180 °C (multimode 2.45 GHz, 1 kW power, 1 min).^[^ ^192^ ^]^ Bismuth nitrate (Bi(NO_3_)_3_·5H_2_O), sodium selenide (Na_2_SeO_3_), and KOH as precursors and EG were used as the solvent. As prepared samples were cold pressed (20 MPa) and sintered for 6 s in the MW oven. The maximum and minimum values of the PFs were 157 µW m^−1^ K^−2^ at 523 K and 120 µW m^−1^ K^−2^ at RT (**Figure** **13**a,b). Single crystalline Sb_2_Te_3_ nanoforks have been prepared via solvothermally (150 °C for 36 h) in the presence of EG, which worked not only as a solvent but also as a reducing agent.^[^ ^193^ ^]^ The Te powder, Sb_2_O_3_, EG, HNO_3_, and polyethylene glycol were used as reactants. The usage of HNO_3_ was found to be crucial in the formation of fork‐like structures. Bi_2_Te_3_ nanostructures with different morphologies: nanoparticles (lateral size of ≈298 nm and a thickness of ≈143 nm), step‐like NPts (size distribution from 200 to 500 nm), and ultrathin nanosheets (aggregated sheet‐like) have been prepared via a ST method and sintered using a fast SPS process.^[^ ^194^ ^]^ EG was used as the solvent. Various morphologies resulted in different nanostructure stacking and, as a result, different degrees of texture after SPS (Figure 13c–f). It was found that high NaOH concentration can accelerate the chemical reaction. Step‐like Bi_2_Te_3_ NPts showed a *zT* of 0.69 at 333 K.

**Figure 14:**
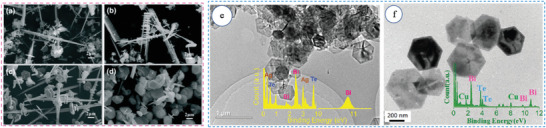
SEM images of the powder samples with different reaction times with the shashlik‐like microstructure: a) 12 h; b) 24 h; c) 36 h; d) 48 h. Reproduced with permission.^[^ ^195^ ^]^ Copyright 2019, RSC; e) TEM image and EDS analysis of Ag‐doped Bi_2_Te_3_; f) TEM image and EDS analysis of Cu‐doped Bi_2_Te_3_. Reproduced with permission.^[^ ^196^ ^]^ Copyright 2019, RSC.

Using one‐pot, template‐free ST method, *n*‐type Te–Bi_2_Te_3_ nanocomposites (Te nanorods and Bi_2_Te_3_ NPts of several μms) were synthesized.^[^ ^195^ ^]^ TeO_2_ and Bi(NO_3_)_3_·5H_2_O were used as the starting materials and NaOH as the regulator and EG as the solvent. In the synthesis steps, Te nanorods appeared first in the solvent and then Bi_2_Te_3_ NPts formed on the surface of the Te nanorods. With increasing reaction time, Te content decreased in the powder sample. When the reaction time approached 48 h, the Te vanished, leaving phase‐pure Bi_2_Te_3_ (**Figure** **14**d). As‐obtained sample was SPSed (673 K, 50 MPa, 5 min). The *PF* was 668 µW m^−1^ K^−2^ at 323 K and decreased to 444 µW m^−1^ K^−2^ at 473 K due to unchanged *S* and decreased *σ*. The *κ*_l_ of 0.437 W m^−1^ K^−1^ at 323 K, 0.416 W/m‐K at 423 K, 0.432 W m^−1^ K^−1^ at 473 K, and a *zT* of 0.54 at 398 K were observed (with a reaction time of 36 h).

M‐doped (*M* = Ag, Cu, In) Bi_2_Te_3_ NPts have been prepared via a ST method and cation exchange reaction (CER).^[^ ^196^ ^]^ In a two‐step process, first Bi_2_Te_3_ NPts (400 nm diameter and 50 nm thickness) were synthesized using a ST method (180 °C for 36 h). As obtained to Bi_2_Te_3_ NPts were doped using CER involving Ag^+^, Cu^2+^, In^3+^. In the preparation and doping the Bi_2_Te_3_ NPts, PVP and EG (as general reagents), BiCl_3_ and K_2_TeO_3_, NaOH and AgNO_3_, Cu(NO_3_)_2_·2.5H_2_O, InC_l3_·3H_2_O were used. The cation doping did not change the lattice, but introduced a few lattice defects and distortions. Figure 14e,f shows Ag and Cu doped Bi_2_Te_3_ NPts.

Bi_2_Te_3_ hollow nanospheres (diameters of 50–100 nm) were prepared using Bi(NO_3_)_3_, TeO_2_, and HNO_3_ in mixed solvents of water and EG by a MW heating method (150 °C for 30 min).^[^ ^197^ ^]^ The shell thicknesses of hollow nanospheres were 20 to 35 nm. Single‐mode MW equipment (commercial 2.45 GHz, maximum 300 W), equipped with a magnetic stirring and a water‐cooled condenser was employed. During the synthesis, a white intermediate product (Bi_2_TeO_5_) appeared after MW heating for 10 min at 150 °C, and finally turned into black Bi_2_Te_3_. This white intermediate product did not change to Bi_2_Te_3_ at temperatures below 130 °C under MW heating. In comparison, the product prepared by a conventional heating method for 30 min was Bi_2_TeO_5_ instead of Bi_2_Te_3_; a mixture of Bi_2_Te_3_, and Te was obtained when the conventional heating time was increased to 11 h; and a single phase of Bi_2_Te_3_ was obtained when the conventional heating time was extended to 24 h.

A MW‐assisted wet chemical method was employed for the synthesis of hexagonal single‐crystalline Sb_2_Te_3_ NPts (edge length approximately hundreds of nm) using Na(SbO)C_4_H_4_O_6_, Te powder, NaOH in EG and 280 W of 2.45 GHz MW radiation for 45 min.^[^ ^198^ ^]^ When ultrasonic irradiation was used for the synthesis, small amount of Sb_2_Te_3_ and unreacted Te were found in the product. When the same reaction mixture was used in ST method using Teflon‐lined autoclave at 180 °C, though Sb_2_Te_3_ phase was observed, the reaction was much slower and not completed even after 48 h. It was observed that, no Sb_2_Te_3_ was formed when water, EtOH, and dimethyl formamide (DMF) were used as solvents.

A mixture of Bi_2_Te_3_ nanorods (width: 20–50 nm; length: 200–400 nm) and hexagon nanoflakes (edge length ≈90 to 150 nm) were prepared using Bi(NO_3_)_3_·5H_2_O, Te powder, KOH, EG, and C_3_H_8_O_3_ via MW‐heating, in which elemental Bi was formed by the reduction of Bi^3+^, and then Bi reacted with Te.^[^ ^199^ ^]^ It was found that if the reaction is conducted for long enough time, the different reaction solvent (i.e., EG, C_3_H_8_O_3_, or mixture of C_3_H_8_O_3_ and water) showed no effect on the XRD results indicating hexagonal Bi_2_Te_3_ phase can be obtained in all these different solvents. When the polyol solvents are overheated, their reducing ability gets enhanced and, therefore, they can easily reduce the metal ion to metal in the zero state. In this experiment, reduced Bi particles reacted with Te to form Bi_2_Te_3_. Suppression of Te vacancies has been realized in *n*‐type nanostructured Bi_2_Te_3_ that is obtained via the ST method (in presence of EG) and SPS.^[^ ^200^ ^]^ Charge carrier concentration was observed to decrease from ≈1 × 10^20^ to ≈6 × 10^19^ cm^−3^ resulting in a decreased *σ* and an increased *S*. As a result a *zT* of ≈1.1 was observed at 420 K.

Bi_2_Te_3_/MWNT nanocomposites were prepared using a MW‐polyol method assisted with titration.^[^ ^201^ ^]^ Bismuth nitrate pentahydrate (Bi(NO_3_)_3_·5H_2_O), sodium tellurite (Na_2_TeO_3_), NaOH, and EG were used in the synthesis. The MW oven (power level from 0 to 1000 W), also fitted with a temperature monitor and magnetic stirring devices was employed. The obtained products were mainly hexagonal Bi_2_Te_3_ NPts attached to the surfaces of MWNTs, while some Bi_2_Te_3_ nanocrystals were encased inside the cores of relatively small MWNTs.

*n*‐type Te/Bi_2_Te_3_ with hierarchical nanostructures have been prepared using Te nanotubes as templates via MW‐ST method.^[^ ^202^ ^]^ First Te nanotubes (length: 2–6 µm; diameters: 100–200 nm) were prepared by MW‐ST treatment. A Teflon vessel containing the mixture of Na_2_TeO_3_, poly(N‐vinyl‐2‐pyrrolidone) (PVP), and EG was heated at 230 °C for 5 min using a commercial lab MW oven. In the second step, Te nanotubes, Bi(NO_3_)_3_·5H_2_O, EG, and NaOH solution were mixed in a Teflon vessel and heated in the same MW oven at 230 °C for 5 min. The powder of Te/Bi_2_Te_3_ hierarchical nanostructures was sintered via SPS (250 °C, 40 MPa, 5 min). The Te/Bi_2_Te_3_ sample exhibited *zT* ≈ 1 (and *κ* ≈ 0.8 W m^−1^ K^−1^ at 440 K) as compared to *zT* of ≈0.75 of pure Bi_2_Te_3_ NPts. The optimized reduced Fermi level, enhanced phonon scattering, and suppressed bipolar conduction contributed to the rise in *zT* of Te/Bi_2_Te_3_ hierarchical nanostructures.

A MW‐ST method has been adapted to fabricate *p*‐type Bi*_x_* Sb_2‐_ *_x_* Te_3_ NPts with a peak *zT* of 1.2 at 320 K (for Bi_0.5_Sb_1.5_Te_3_), which is attributed to a high PF of 28.3 × 10^–4^ W m^−1^ K^−2^ and a low *κ* of 0.7 W m^−1^ K^−1^.^[^ ^203^ ^]^ For the synthesis of Bi*_x_* Sb_2‐_ *_x_* Te_3_, SbCl_3_, Bi(NO_3_)_3_·5H_2_O, Na_2_TeO_3_, NaOH, and EG were used. A commercial lab MW oven was employed to heat the reaction mixture in a Teflon vessel at 230 °C for 10 min and the obtained NPt powders were consolidated via SPS (250 °C, 40 MPa, 5 min). The avg. grain size of sintered Bi_0.5_Sb_1.5_Te_3_ was ≈ 900 nm. Considerable reduction in *κ *(≈0.7 W m^−1^ K^−1^) was due to the presence of dense grain boundaries, dislocations in nanostructures, and Bi–Sb lattice disorders.

Room temperature ionic liquid (RTIL) of 1‐butyl‐3‐methylimidazolium bromide (C_8_H_15_BrN_2_) consists of cation [C_8_H_15_N_2_]^+^ and anion Br^−^. The high ionic conductivity and polarizability of [C_8_H_15_N_2_]^+^ make it an efficient MW‐absorbing agent. Bi_2_Te_3_ plate‐like crystals with hexagonal morphology were prepared via MW‐assisted heating (10 min; 800 W, 2.45 GHz) of a reaction mixture containing Te powder, Bi(NO_3_)_3_·_5_H_2_O, EG, KOH, and ionic liquid C_8_H_15_BrN_2_.^[^ ^204^ ^]^ The Bi_2_Te_3_ plates were found to be single‐crystal in nature with the growth direction of {11$\bar{2}$0}; edge length of ≈0.5–2 µm, and the thickness of <100 nm. The amount of C_8_H_15_BrN_2_ played an important role in the formation of Bi_2_Te_3_ crystals with different morphologies. At 473 K, a maximum *S* of ‐152 µV K^−1^ was observed.

The ionic liquid, *N*‐butylpyridinium tetrafluoroborate (BuPy^+^[BF_4_]^–1^) consists of cation BuPy^+^ and anion [BF_4_]^–1^. Due to high ionic conductivity and polarizability of cation, BuPy^+^, this ionic liquid is an excellent MW absorbing agent. Using BuPy^+^[BF_4_]^–1^ as the solvent, Te nanostructures of different shapes and sizes were prepared (nanorods: diameters ≈ 15–40 nm, lengths ≈ 700 nm; nanowires: diameters ≈ 20–100 nm, lengths: tens of μms, and spherical particles).^[^ ^205^ ^]^ A commercial MW oven (2.45 GHz, 10 W, 40 s) was used to irradiate the samples to 180 °C. Temperature was shown to be an important factor in controlling the morphology of Te nanostructures. Even at RT, NaBH_4_ reacted with TeO_2_ to form Te. Between the RT and 130 °C, no nanorods or NWs were observed. In the range 130 °C to 150 °C, Te nanorods plus spherical nanoparticles were observed; close to 180 °C and beyond, only nanorods were observed. When the temperature was >200 °C, the diameters of nanorods increased significantly. The optimum temperature for the synthesis of Te nanorods or NWs was ≈180 °C. It was speculated that the BuPy^+^ ions acted as a capping reagent and bonded to the {001} facets less strongly than to other facets. The Te atoms adhered to (001) facets facilitated the preferential growth along [001] zone axis (*c*‐axis of the crystal lattice).

Single‐crystalline Bi_2_S_3_ and Sb_2_S_3_ nanorods were synthesized via MW‐assisted method using different combinations of EG, ethanolamine, and the ionic liquid, 1‐butyl‐3‐methylimidazolium tetrafluoroborate ([Bmim][BF_4_]).^[^ ^206^ ^]^ Single‐mode commercial MW equipment was used to irradiate the sample mixture to 190 °C (30 s or 10 min). The ionic liquid was shown to play a significant influence in regulating the morphology of M_2_S_3_ (M = Bi, Sb) nanostructures. In the presence of [Bmim][BF_4_], longer and thinner Bi_2_S_3_ nanorods were observed and in absence, urchinlike Bi_2_S_3_ structures were formed. It was also speculated that [Bmim][BF_4_] may act as a surfactant in the formation of Bi_2_S_3_ nanorods very similar to the Te nanorods.^[^ ^205^ ^]^ Irregularly shaped single‐crystalline Sb_2_S_3_ nanosheets were also prepared in the absence of [Bmim][BF_4_] and single‐crystalline Sb_2_S_3_ nanorods in presence of [Bmim][BF_4_].

As previously stated, chalcogen elements like as S, Se, and Te are soluble in phosphonium ILs at high temperatures. Phosphonium ILs, tetrabutylphosphonium halides ([P_4444_]X, X = Cl, Br) were used to prepare PbTe and PbSe nanostructured metal chalcogenides. The phosphonium halide ILs also simultaneously allowed the halogen atoms to be doped in metal chalcogenides to prepare *n*‐type TE materials. The PbTe material prepared in [P_4444_]Br exhibited a relatively high *PF* as compared to other counterparts due to its high *S*.^[^ ^171^ ^]^ The IL, [C_4_mim]Br (C_4_mim = 1‐butyl‐3‐methylimidazolium) was used to prepare Sb_2_Te_3_ nanomaterial, which exhibited *zT* ≈ 1.5 at 300 °C and *κ* of 0.29 to 0.27 W m^−1^ K^−1^.^[^ ^207^ ^]^ The mixture of a single‐source precursor, (Et_2_Sb)_2_Te and [C_4_mim]Br was heated in a commercial MW equipment for 30 s at 100 °C, 5 s at 150 °C, and finally for 5 min at 170 °C.

Bi_2_Te_3_ nanoparticles with average sizes of 15–20 nm and NWs (diameter < 100 nm; length of ≈10 µm) have been prepared by ST synthesis (150 °C for 24 h) using BiCl_3_ and Te powder as the precursor, NaBH_4_ as the reducing agent and organic solvents: ethylenediamine (EN), DMF, pyridine (C_5_H_5_N), C_3_H_6_O, and CH_3_CH_2_OH or distilled water.^[^ ^208^ ^]^ NaOH was used to control the pH of the solution. The phase distributions, microstructures, and grain sizes of nanostructured Bi_2_Te_3_ were shown to be primarily influenced by the dielectric constant and surface tension of the solvent during the ST synthesis. A substantial fraction of the product contained NWs (diameter of <100 nm; length of 10 µm) when distilled water was used. Organic solvents, on the other hand, produced metallic bismuth, tellurium, and bismoclite.

Uniform Sb nanotubes (middle‐hollow, open‐ended structures, multiwalled structures with the wall thickness of ≈10 nm) were synthesized through a ST method without using any surfactants or templates.^[^ ^209^ ^]^ The Sb nanotubes showed a rhombohedral phase, better crystallinity, and had an average diameter of ≈50 nm and a length of ≈350 nm. During the synthesis, Sb nanotubes are assumed to be formed by the rolling up of regularly arranged molecular layers, with toluene acting as a structure guiding agent and perhaps providing a driving force to facilitate the rolling of lamellar structures into nanotubes.

**Figure 15:**
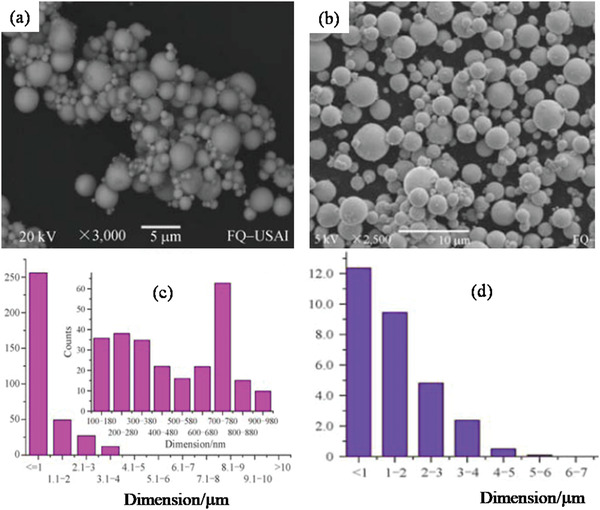
a) SEM images of metallic Bi synthesized at 600 W with the precursor concentrations of 0.05 mol L^−1^; b) SEM images of metallic Bi synthesized at 1200 W with the precursor concentrations of 0.05 mol L^−1^. c,d) Images show the histograms of sphere dimension in µm. Adapted with permission.^[^ ^210^ ^]^ Copyright 2016, Higher Education Press and Springer‐Verlag.

Metallic Bi spheres (45 nm–10 µm) were prepared via MW‐ST method. At 600 W (2.64 GHz), Bi size was dependent on the concentration of precursors solution.^[^ ^210^ ^]^ When the concentration was 0.01 mol L^−1^, the average size of Bi spheres was ≈500 nm; for the same power level and the concentration of 0.05 mol L^−1^, the size of sphere increased to 1–5 µm (**Figure** **15**). At a power level of 1200 W, the avg. size of spheres was 500 nm–1 µm. A monoclinic phase of Bi_2_O_3_ (flake‐like morphology) was obtained when the power level was reduced to 120 W.

Bi_2_Se_3_(bulk bandgap of ≈0.3 eV) is known as a topological insulator.^[^ ^211^ ^]^ Bi_2_Se_3_ nanosheets with average thickness of 1, 4, 7, and 13 nm have been fabricated using MW‐stimulated ST method.^[^ ^212^ ^]^ Bi(NO_3_)_3_·5H_2_O, SeO_2_, EG, NaOH, and the surfactant, PVP were used for the synthesis. A commercial MW‐oven was used to heat the reaction mixture at 230 °C for 5 min and SPS (250 °C, 40 MPa, 5 min) was employed for the consolidation. TE property measurements showed a significantly reduced *κ* (0.41 W m^−1^ K^−1^), enhanced PF (4.71 × 10^–4^ W m^−1^ K^−2^ with *S* = −155.32 µV K^−1^ and *σ* = 1.96 × 10^4^ Ω^−1^ m^−1^) culminating in a *zT* ≈ 0.48 at 427 K in the pellet composed of single‐layered Bi_2_Se_3_ nanosheets. Similarly, Bi_2_Se_3_ hexagonal nanoflakes (cross section of ≈6 µm), nanoflower and octahedral agglomerated crystals of nearly ≈60 nm size have been synthesized via MW‐assisted (commercial, 300 W) ST process using EG as a solvent.^[^ ^213^ ^]^

A series of *n*‐type Bi‐doped PbTe such as Pb_1_ *_−x_* Bi*_x_* Te (*x* = 0.00, 0.01, 0.02, 0.03 and 0.04) alloys were synthesized by MW‐HT method followed by hot‐pressing (773 K, 20 MPa, 1 h).^[^ ^214^ ^]^ The synthesized sample had a single cubic crystal structure, compact microstructures, and a uniform element distribution. The carrier concentration improved with an increase in Bi content. A higher PF of 8.5 µW cm^−1^ K^−2^ was observed at 623 K for *x* = 0.02. Besides, for the same sample, a lower *κ*_l_ of ≈0.68 W m^−1^ K^−1^ was obtained at 673 K due to alloy scattering, grain boundary scattering, dislocations, and defects culminating in a *zT* of 0.62 at 673 K (for Pb_0.98_Bi_0.02_Te), which is akin to *zT* of PbTe alloys obtained via a conventional melting method.

##### PbTe, Cu_2_Te, SnTe, SnSe Based and Other Materials

PbTe hierarchical nanostructures have been synthesized by a ST method (180 °C for 18 h) in mixtures of water and ethanol at 180 °C with the help of glucose.^[^ ^215^ ^]^ The nucleation, growth, and self‐assembly of the PbTe nanosheets may be controlled, resulting in diverse morphologies via altering the experimental settings: the amount of NaOH, volume ratio of ethanol/water, reaction duration, and concentration of glucose. Lead acetate (Pb(C_2_H_3_O_2_)_2_), sodium tellurite (Na_2_TeO_3_), glucose, NaOH, C_2_H_5_OH, and hydrazine (N_2_H_4_) were used in the synthesis. Cold‐pressed, PbTe micropeony‐like structures exhibited a *σ* of 120.4 Ω^−1^ cm^−1^ at 700 K, while hollow sphere‐like PbTe showed a 82.8 Ω^−1^ cm^−1^ at the same temperature. The suggested micropeony structure formation was a combination of the Ostwald ripening and anisotropic growth processes. Nanostructured binary metal telluride, PbTe was prepared via MW‐assisted polyol method using EG was used as a reducing agent.^[^ ^216^ ^]^ No product was observed when the reaction was performed using a conventional heating method.

**Figure 16:**
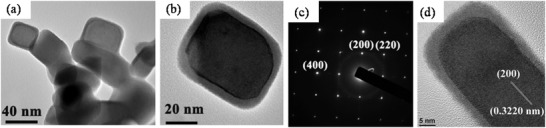
Sb‐doped PbTe/Ag_2_Te core–shell structure. a,b) TEM micrographs. c) SAED pattern taken from the core of a single nanocube. d) HRTEM image of a single nanocube. Reproduced with permission.^[^ ^217^ ^]^ Copyright 2012, Elsevier.

The fabrication of nanocomposites comprising nanostructured metal telluride is substantially more complicated and difficult than that of nanocomposites incorporating metal or metal oxide nanoparticles.^[^ ^174^ ^]^ Dong et al. reported the MW‐ST synthesis of Sb‐doped PbTe/Ag_2_Te core–shell composite nanocubes, wherein the core is made of crystallized cubic Sb‐doped PbTe and the shell of amorphous Ag_2_Te.^[^ ^217^ ^]^ For the synthesis, SbCl_3_, Na_2_TeO_3_, AgNO_3_, Pb(CH_3_COO)_2_·3H_2_O, NaOH, and NaBH_4_ were used as the reactants, and EG was used as the solvent. These reactants were dissolved in a preheated EG (80 °C) and heated to 250 °C in 20 min in a MW (2.45 GHz, maximum power 1000 W). The obtained product consisted of nanocubes (edge length ≈ 25–55 nm) filled with crystallized nanocube as a core (edge lengths of 20–50 nm) and an amorphous outer shell with thicknesses of 3–6 nm (**Figure** **16**).

**Figure 17:**
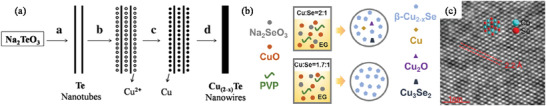
a) The proposed formation process of Cu_2−_ *_x_* Te NWs. Reproduced with permission.^[^ ^218^ ^]^ Copyright 2012, Elsevier. b) The schematic representation of the ST synthesis technique, as well as the impact of lowering the Cu/Se precursor ratio on the impurities of as‐synthesized Cu_2−_ *_x_* Se powders. c) HRTEM image of the Cu_1.7_Se particle. Reproduced with permission.^[^ ^219^ ^]^ Copyright 2019, Elsevier.

**Figure 18:**
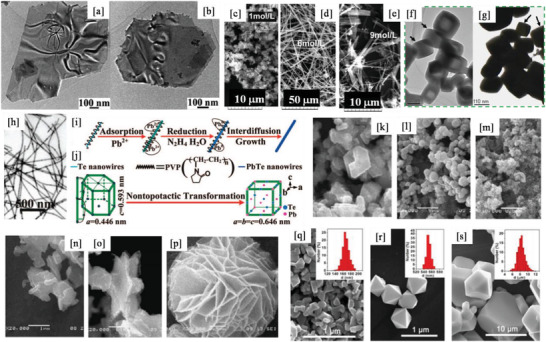
a,b) Bright‐field TEM images of Cu_1.75_Te nanosheets. Adapted with permission.^[^ ^59b^ ^]^ Copyright 2014, RSC. SEM images of Cu_2‐_ *_x_* Te samples synthesized under different KOH concentrations in 10 mL H_2_O c) 1 mol L^−1^; d) 6 mol L^−1^; e) 9 mol L^−1^. Reproduced with permission.^[^ ^221^ ^]^ Copyright 2011, Elsevier Masson SAS; f) TEM images of hollow PbTe nanocubes prepared by using EtOH as solvent in the presence of nonionic surfactant PEG at 160 °C for 20 h. g) TEM images of the as‐prepared solid PbTe nanocubes by using EG as solvent. Adapted with permission.^[^ ^222^ ^]^ Copyright 2012, Elsevier. h) TEM image of PbTe NWs synthesized at 373 K for 12 h. Illustration of the i) growth mechanism and j) structural change involved in the transformation from *t*‐Te NWs to PbTe NWs. Adapted with permission.^[^ ^223^ ^]^ Copyright 2008, ACS. SEM images of as‐prepared CuS products with 0.25 mol L^−1^ Cu(ac)_2_ and *C*_R_ = 3 (Tu:Cu(ac)_2_ ratio) at 160 °C for 24 h in different solvents: k) water, l) ethanol–water (1:1), m) EtOH, n) ethylenediamine, o) ethylene glycol‐ether, p) benzene. Adapted with permission.^[^ ^224^ ^]^ Copyright 2007, Elsevier. q–s) SEM images of SnTe NPs synthesized with different dosage of NaOH. Adapted with permission.^[^ ^225^ ^]^ Copyright 2016, Elsevier.

Using Te nanotubes as self‐sacrificial templates, single‐crystalline Cu_2‐_ *_x_* Te NWs (diameters of 100–200 nm; lengths of several μms) have been prepared via MW‐ST method.^[^ ^218^ ^]^ Sodium tellurate (Na_2_TeO_3_), PVP, and EG were used for ST synthesis of Te nanotubes; and Te nanotubes, copper nitrate trihydrate Cu(NO_3_)_2._3H_2_O), EG, and NaBH_4_ were used for MW‐ST synthesis of Cu_(2‐_ *_x_*_)_Te NWs. The *σ* values were found to be (5.99–7.19) × 10^3^ Ω^−1^ m^−1^ in 300–450 K range for a cold‐pressed Cu_2‐_ *_x_* Te NW powder. As per the proposed Cu_2‐_ *_x_* Te NW formation, a) Na_2_TeO_3_ is reduced by EG (acts as both the reducing reagent/solvent to form elemental Te nanotubes; b) Cu^2+^ cations adsorb on the Te nanotubes template; c) Cu^2+^ cations are reduced to Cu by NaBH_4_; d) reaction occurs between elemental Cu and Te to form Cu_2−_ *_x_* Te NWs (**Figure** **17**a). In a ST synthesis, a high purity *β*‐Cu_2‐_ *_x_* Se phase (microscale nanoparticles) was obtained by modifying the reaction kinetic condition through tuning the Cu/Se precursor ratio.^[^ ^219^ ^]^ As shown in Figure 17b, by decreasing the Cu/Se precursor ratio from 2:1 to 1.7:1 (viz Cu_2_Se, Cu_1.9_Se, Cu_1.8_Se and Cu_1.7_Se), impurities such as Cu, Cu_2_O, and Cu_3_Se_2_ were eliminated. After SPS, the porous *p*‐type Cu_1.7_Se pellet exhibited a low *κ* of ≈0.24 W m^−1^ K^−1^ at 773 K. Single crystalline 2D nanosheets of *p*‐type Cu_1.75_Te (**Figure** **18**a,b) were prepared using a ST reaction (200 °C for 8 h) using a colloidal dispersion of dodecylsulfate intercalated copper hydroxide layers in EG and an alkaline solution of TeO_2_.^[^ ^59b^ ^]^

In the reaction, EG reduces TeO_2_ to Te^2–^ and Cu^2+^ to Cu^+^ leading to the formation of Cu_1.75_Te and the solvated copper hydroxide layers functioned as templates to facilitate the formation of nanosheets. For the cold‐pressed Cu_1.75_Te nanosheet powder, the maximum value of PF was 6 × 10^–5^ W m^−1^ K^−2^ at 650 K in comparison to PF of bulk Cu_2_Te which was 1.4 × 10^–4^ W m^−1^ K^−2^ at 650 K.^[^ ^220^ ^]^ There was a large reduction in the *κ* for the nanosheet material, i.e., ≈1 W m^−1^ K^−1^, in comparison to 5–3 W m^−1^ K^−1^ for the bulk Cu_2_Te.^[^ ^220^ ^]^

Without using any template or capping agent, Cu_2‐_ *_x_* Te NWs (lengths approximately hundreds of µm, several hundred nm in diameter) were obtained by HT route (200 °C, 24 h), where the HT environment was controlled by adjusting the KOH concentration in the reaction solution.^[^ ^221^ ^]^ The Te powder, KOH, CuCl_2_.2H_2_O, and hydrazine hydrate (as a reducing agent) were used in the synthesis. It was found that the KOH concentration in solution can effectively adjust the morphology of the end product; for below 1 mol L^−1^, NPts and above 6 mol L^−1^, NWs were formed (Figure 18c–e). Also, for these single‐crystal NWs, optical energy gap was 1.1 eV (direct band) and 0.7 eV (indirect band) and these NWs showed metal‐like conductivity.

HT synthesis of PbTe using EtOH as a solvent in presence of nonionic polymer polyethylene glycol (PEG) yielded hollow PbTe cubes (edge length ≈ 60–250 nm, Figure 18f)), whereas using EG as a solvent in presence of PEG with the same experimental conditions (120 °C, 20 h) produced solid PbTe cubes (edge lengths of 80–160 nm, Figure 18f,g).^[^ ^222^ ^]^ The results indicate that the solvents apparently played a key role in determining the interior structure of PbTe. It was concluded that nonionic polymer PEG played an important role in the controllable growth of cubic PbTe nanocrystals in both cases. Since EtOH and EG have different viscosity, it was proposed that viscosity has a considerable effect on shaping the interior features of PbTe nanocrystals.

A two‐step HT process has been employed in the synthesis of PbTe NWs.^[^ ^223^ ^]^ In this process, first, Te NWs were prepared by the HT treatment (453 K, 24 h) of Na_2_TeO_3_ and PVP, with hydrazine hydrate and aqueous ammonia added. In a second step, Pb(NO_3_)_2_ is added to the above solution, followed by a second HT treatment at 373 K for 12 h. The pure chalcogen nanostructures synthesized in the first step acted as a sacrificial template to grow PbTe NWs. The process yielded PbTe NWs 20–40 nm in diameter and with lengths of ≈100 nm (Figure 18h). The proposed in‐situ diffusion and growth mechanism involves the interaction of the PVP encapsulated Te NW template with Pb^2+^. Since PVP can strongly bind metal cations,^[^ ^226^ ^]^ it traps the Pb^2+^ at the surface of the Te NWs and the residual hydrazine reduces the Pb^2+^ to Pb(0) in situ. The Pb atoms diffuse into the Te NWs leading to the PbTe NWs formation in a nontopotactic reaction (Figure 18i,j). The *S* of the NW film was 628 µV K^−1^.

The effect of temperature, molar ratio, concentration of the precursor and solvent on the morphology and composition of CuS nanocrystals during HT treatment without surfactant assistance is reported.^[^ ^224^ ^]^ In this study, a series of reactions, Cu(ac)_2_ complexed by citric acid with thiourea were conducted for 24 h at a 433 K temperature. Depending on the solvent, various morphologies of CuS were obtained (Figure 18k–p). The results showed that the morphology of CuS particles depends on the nucleation process and the homogenous nucleation is in favor of the formation of different shapes. Solvents with more coordination with Cu^2+^: ethylenediamine and EG are easily anchored onto the surface of the crystal structure to form complexes that obstruct Te from getting attached to Cu^2+^ and help in the formation of smaller CuS particles.

In a MW‐HT method (550 W), SnTe nanoparticles of different shapes and sizes 165 nm to 8.2 µm (Figure 18q–s) were produced by adjusting the dosage of NaOH and densified via SPS (723 K, 80 MPa, 8 min).^[^ ^225^ ^]^ Due to a low *κ* of 0.60 W m^−1^ K^−1^, a *zT* ≈ 0.49 at 803 K was observed in the 165 nm nanoparticles. Many methods have been employed in synthesizing copper selenide, such as heating Cu and Se powder mixtures to 400–470 °C under Ar flow; using toxic H_2_Se as the source,^[^ ^227^ ^]^ or mechanical alloying of Se and Cu with high‐energy ball milling.^[^ ^228^ ^]^ Parkin et al. reported a RT route to selenides by the reaction of selenium with elemental metals in liquid ammonia in a thick‐walled glass vessel.^[^ ^229^ ^]^ In this method, many operations were carried out at −77 °C, and since reactions in liquid ammonia have been known to explode,^[^ ^229^ ^]^ all activities had to be carried out behind a safety screen. In a one‐step ST method to obtain nanocrystalline Cu_2−_ *_x_* Se at a low temperature, ethylenediamine as a solvent coordinated to Cu^+^ presumably played an important role in the formation of nanocrystalline Cu_2−_ *_x_* Se.^[^ ^227^ ^]^

Cu_3_SbSe_4_, a ternary *p*‐type semiconducting compound with a bandgap of 0.4 eV and a *μ *of 135 cm^2^ V^−1^ s^−1^ at RT,^[^ ^230^ ^]^ is regarded as a significant material among Cu‐based TE materials. A series of (Ag, Sn) codoped Cu_3_SbSe_4_ nanocrystals (Cu_3−_ *_y_* Ag_1−_ *_y_* Sb_1−_ *_x_* Sn*_x_* Se_4_) were prepared via MW‐ST method (493 K, 15 min) and sintered via SPS (623 K, 40 MPa, 4 min).^[^ ^231^ ^]^ Sn‐doping on Sb sites increased the *σ* due to increased carrier concentration. Alloying with Ag on Cu sites reduced the *κ*_l_ appreciably (*κ* = 0.44 W m^−1^ K^−1^ and *κ*_l_ = 0.27 W m^−1^ K^−1^). In addition to point defect scattering, the formation of Cu_2−_ *_x_* Se nanoinclusions also served as additional scattering centers for phonons. The sample, with a composition of Cu_2.8_Ag_0.2_Sb_0.95_Sn_0.05_Se_4_ yielded a *zT* of 1.18 at 623 K.

HT synthesis (120 °C for 12 h) followed by MW sintering has been used for the fabrication of Bi_2_S_3_ doped PbS.^[^ ^232^ ^]^ MW sintering was performed at 773 K in N_2_ atmosphere for 1 hour using 2.450 GHz MWs. Due to enhanced *σ* by Bi_2_S_3_ doping and the reduced *κ* induced by the microstructures, the *zT* of MW‐sintered Pb_0.995_Bi_0.005_S and Pb_0.99_Bi_0.01_S attained 0.90 and 0.86 at 800 K, respectively. When sintered via plasma‐activated sintering, the *zT* value of Pb_0.99_Bi_0.01_S reached only 0.3 at 800 K. PbS had a star shape with arms composed of 30 nm nanoparticles. The proposed mechanism for the formation of star‐shaped PbS is the crystal growth along the six <111> directions of cubic structure.

**Figure 19:**
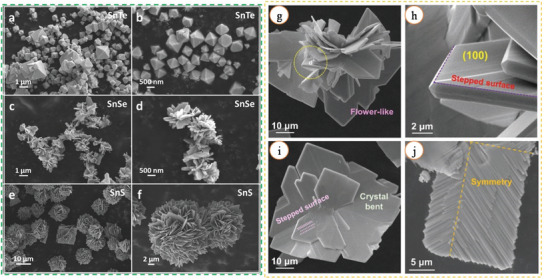
Left side frame. a,b) SEM images of SnTe powders. c,d) SnSe powders. e,f) SnS powders. Reproduced with permission.^[^ ^233^ ^]^ Copyright 2017, IOP Publishing. g) Magnified SEM image of flower‐like SnSb_0.03_Se_0.94_ microplates. h) Magnified SEM image of SnSb_0.03_Se_0.94_ microplates circled in panel g) to show stepped (100) surface; i) Magnified SEM image of one SnSb_0.03_Se_0.94_ microplate with circled imperfections and crystal bent; j) magnified SEM image of one cracked SnSb_0.03_Se_0.94_ microplate with labeled symmetry. Reproduced with permission.^[^ ^234^ ^]^ Copyright 2018, Wiley‐VCH.

Single‐phase and lead‐free IV–VI group semiconductor members: SnTe, SnSe, and SnS nanopowders were synthesized by a HT method (100 ℃ for 12 h) with SnCl_2_ as Sn source and elemental Te/Se/S as chalcogen source.^[^ ^233^ ^]^ Hydrazine hydrate acted as a reducing agent; deionized water and EG were used as the solvents and NaOH was used to adjust the pH. The SnTe particles with an octahedron structure and SnSe/SnS particles with a plate‐like shape (**Figure** **19**a–f) were obtained and then compacted by SPS (50 MPa, 5 min for all) and at sintering temperatures of 500 °C, 450 °C, and 650 °C, for SnTe, SnSe, and SnS samples respectively. The layered crystal structure of SnSe and SnS resulted in considerable anisotropy in both electrical and thermal characteristics. At 873 K, *zT* values for SnTe, SnSe, and SnS bulk samples were 0.79, 0.21, and 0.13, respectively.

The *n*‐type Sb‐doped polycrystalline SnSe microplates were synthesized using a ST method (230 °C for 36 h) and SPSed (573 °C, 60 MPa, 5 min).^[^ ^234^ ^]^ The sintered SnSb_0.03_Se_0.94_ microplate samples (tens of µm in size) showed a PF of ≈2.4 µW cm^−1^ K^−2^ and a very low *κ* of ≈0.17 W m^−1^ K^−1^ at 773 K, leading to a high *zT* of ≈1.1 at 773 K. The higher PF was attributed to Sb‐enabled electron doping, which resulted in a high electron concentration of 3.94 × 10^19^ cm^−3^. The low *κ *was attributable to enhanced phonon scattering at crystal defects, which included severe lattice distortions, dislocations, and lattice bents (Figure 19g–j).

**Figure 20:**
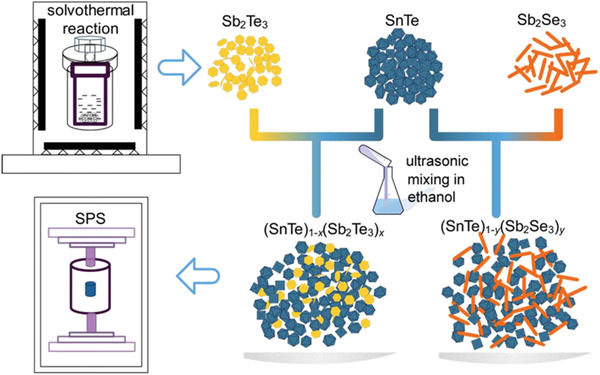
Schematic illustration of (SnTe)_1‐_ *_x_* (Sb_2_Te_3_)*_x_* (*x* = 0.03, 0.06, 0.10) and (SnTe)_1‐_ *_y_* (Sb_2_Se_3_)*_y_* (*y* = 0.03, 0.06) fabrication processes. Adapted with permission.^[^ ^235^ ^]^ Copyright 2020, ACS.

The vacancy and substitutional point defect generation in SnTe‐based materials through sintering SnTe and Sb_2_Te_3_ (or Sb_2_Se_3_) nano/microstructures that were synthesized via ST method using EG and mixture of EG and EtOH as solvents respectively has been demonstrated (**Figure** **20**).^[^ ^235^ ^]^ In this method, (SnTe)_1‐_ *_x_* (Sb_2_Te_3_)*_x_* (*x* = 0.03, 0.06, 0.10) and (SnTe)_1‐_ *_y_* (Sb_2_Se_3_)*_y_* (*y* = 0.03, 0.06) were fabricated by mixing and sintering the nano/microstructured SnTe octahedral particles, Sb_2_Te_3_ NPts, Sb_2_Se_3_ nanorods. The samples were consolidated via SPS (480 °C, ≈40 MPa, 5 min). The defects significantly enhanced the phonon scattering, sharply reducing the *κ*_l_ (1.40 W m^−1^ K^−1^ and 1.26 W m^−1^ K^−1^ at RT) of *x* = 0.06 and *y* = 0.06 samples respectively as compared to 3.73 W W m^−1^ K^−1^ for pristine SnTe. The *zTs* of 0.6 and 0.7 at 813 K were observed in (SnTe)_0.90_(Sb_2_Te_3_)_0.10_ and (SnTe)_0.94_(Sb_2_Se_3_)_0.06_, respectively.

In/Sr codoped SnTe nanomaterial (obtained via ST method in presence of EG) has shown a *zT* of ≈1.31 at 823 K (364% higher than the *zT* of undoped SnTe at the same temperature).^[^ ^236^ ^]^ The codoping significantly improved the electrical transport properties due to the interaction of band‐structure modifications and reduced the *κ*_l_ via strong phonon scattering by point defects, nanoprecipitates, and grain boundaries.

**Figure 21:**
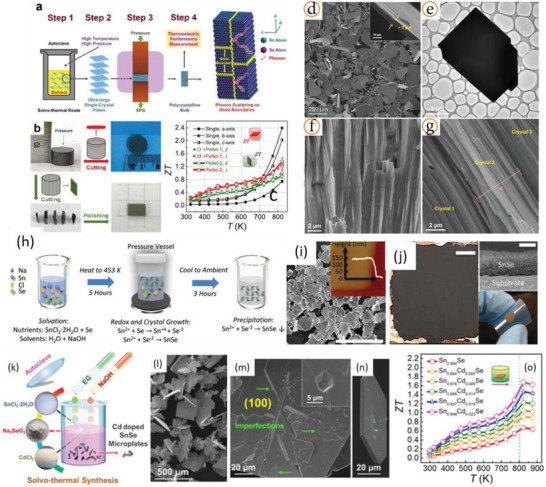
a) Fabrication process of polycrystalline Sn_0.98_Se plates; b) illustration of preparation for Sn_0.98_Se samples with different directions (red for ⊥ and green for // to the sintering pressure) from the pellet; c) measured *zT* values from pellets from current study (Pellet‐1 and Pellet‐2), compared with SnSe single crystals measured along different axes (black).^[^ ^46^ ^]^ SEM image of synthesized products. d) Product‐2; e) TEM image of a typical Sn_0.98_Se plate; Sintered Sn_0.98_Se pellets. f) Pellet‐2 surfaces fractured from the ⊥ directions, g) Pellet‐2 surfaces fractured from the // directions. Reproduced with permission.^[^ ^237^ ^]^ Copyright 2018, Elsevier. h) HT synthesis of SnSe nanosheets; i) SEM image of SnSe nanomaterials (10 µm scale bar); j) optical image of SnSe thin film (5 mm scale bar.(j top right): cross‐section of SnSe thin film and j) bottom‐right: SnSe thin film on flexible substrate. Adapted with permission.^[^ ^238^ ^]^ Copyright 2019, Wiley. k) ST synthesis of Sn_0.948_Cd_0.023_Se; l) SEM image of synthesized flower‐like Sn_0.948_Cd_0.023_Se microplates; m) magnified SEM image of a Sn_0.948_Cd_0.023_Se microplate showing (100) surface with crystal imperfections (inset); n) Sn_0.948_Cd_0.023_Se microplate; o) *zT* versus temperature for Cd‐doped SnSe pellets. Adapted with permission.^[^ ^239^ ^]^ Copyright 2019, Wiley‐VCH.

A peak *zT* of 1.36 was observed in polycrystalline *p*‐type Sn_0.98_Se macrosized plates synthesized via ST route (230 °C for 3 h) and sintered via SPS (573 °C, 60 MPa, 5 min).^[^ ^237^ ^]^ Na_2_SeO_3_, SnCl_2_·2H_2_O, EG, and NaOH were used as precursors. EG served as both a solvent and a reducing agent during the process. A high charge carrier concentration (1.5 × 10^19^ cm^−3^) was achieved by self‐doping via adjusting the synthesis parameters. A *σ* ≈ 72.4 Ω cm^−1^, peak PF of 6.95 µW cm^−1^ K^−2^ and a low *κ* of 0.42 W m^−1^ K^−1^ resulted in a *zT* of 1.36 at 823 K (**Figure** **21**c).

The 2D nanosheets of SnSe have been prepared using a surfactant‐free HT method.^[^ ^238^ ^]^ The schematic of the synthesis procedure is shown in Figure 21h. The SEM image of such SnSe nanosheets is shown in Figure 21i along with AFM image inset showing the thickness ≈150 nm. A thin film of SnSe nanosheet was prepared by drop‐casting onto a copper tape substrate (area of 1 cm^2^). A large *S* of >1200 µV K^−1^ and a very low *κ* of < 0.1 W m^−1^ K^−1^ were observed in the temperature 300–380 K range. However, *σ* for these samples was low (<2.5 Ω^−1^ cm^−1^). The plausible explanation for the increase in *S* was the filtering effect,^[^ ^7^ ^]^ which arose due to the existence of high‐density interfaces. It has been observed that introduction of Cd atoms in SnSe lattice leads to Sn vacancies (*p*‐type doping). In one such study,^[^ ^239^ ^]^ ST synthesis and fast SPS boosted the Sn vacancy to a level of ≈2.9% resulting in an optimal hole concentration of ≈2.6 × 10^19^ cm^−3^ and an improved *PF* of ≈6.9 µW cm^−1^ K^−2^. In Cd‐doped SnSe, Sn vacancy formation energy reduced from 1.63 to 1.50 eV. In addition, doping resulted in large nanoscale crystal imperfections: dislocations, local lattice distortions, and point defects, all contributed to a low *κ* of ≈0.33 W m^−1^ K^−1^ and a high *zT* of ≈1.7 at 823 K in Sn_0.948_Cd_0.023_Se (Figure 21o).

In a MW‐HT synthesis of SnSe microrods, the NaOH concentration in solution precursors is shown to be a key factor in influencing the diameter and morphology, phase composition, and TE properties of the end products.^[^ ^240^ ^]^ In this study, different amounts of NaOH: 0, 100, 200, and 300 mmol, yielding NaOH:SnCl_2_ molar ratios of 0, 10, 20, and 30, respectively were used. The particles obtained without NaOH contained SnO_2_ and SnSe_2_ as impurity phases in addition to SnSe particles (with *zT* ≈ 0.11 at 773 K along ⊥ to the pressing direction). On the other hand, when NaOH:SnCl_2_ molar ratio of 30 was used, only a small amount of SnO_2_ was observed. For this sample, *zT* of ≈ 0.78 and 1.08 at 773 K were observed along ⊥ and || to the pressing direction. The improved *zT* was ascribed to reduced *κ* and enhanced PF. The TE property of single‐crystalline *p*‐type SnSe nanobelts (width ≈ 150–350 nm) with growth direction along the *b* axis synthesized using a ST technique (200 °C for 24 h) has been reported.^[^ ^241^ ^]^ Elemental Se powder and tin chloride dihydrate (SnCl_2_·2H_2_O) were selected as Se and Sn sources and ethanolamine was used as the solvent. The hot‐pressed nanobelt material (923 K, 50 MPa, 10 min) showed a lower *κ* and *σ* compared to its polycrystalline SnSe counterpart. A high *zT* value of ≈0.83 along the in‐plane direction at 803 K was observed. This was about a 60% improvement over the value of 0.5 for pure‐phase SnSe polycrystalline samples.^[^ ^242^ ^]^

In the case of Cu doping in SnSe, Cu‐doped SnSe microbelts (width ≈ 150 – 350 nm) were synthesized via ST method.^[^ ^243^ ^]^ Cu solubility limit of 11.8 was observed. In this study, Na_2_SeO_3_, SnCl_2_ ·2H_2_O, and CuCl_2_ were used as Se, Sn, and Cu doping sources respectively. The powders of SnSe microbelts were sintered by SPS (900 K, 60 MPa, 5 min). A PF of 5.57 µW cm^−1^ K^−2^ and a low *κ* of 0.32 W m^−1^ K^−1^ at 823 K culminated in a peak *zT* of ≈1.41. The morphology of synthesized Sn_1‐_ *_x_* Cu*_x_* Se (*x* = 0 to 0.118) changed from rectangular microplate to microbelts by increasing the Cu doping level. When excess Cu was doped, secondary phase (Cu_2_Se) in the synthesized products was observed but were eliminated through sonic separation and centrifuge techniques. Crystal imperfections such as lattice distortions, dislocations, and strains reduced the *κ* through phonon scattering. For the more analysis on the HT/ST synthesis of SnSe nanomaterials, readers are urged to refer to an article by Shi et al.^[^ ^244^ ^]^

#### 2.3.5 Hydrothermal/Solvothermal Nanoplating Technique

**Figure 22:**
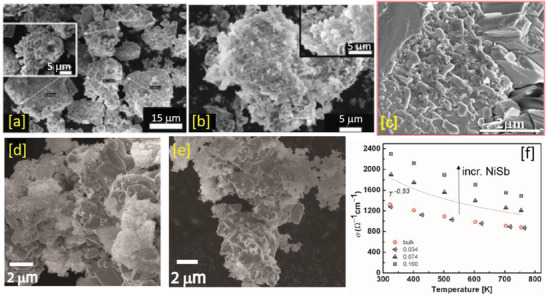
a,b) Bulk CoSb_3_ grains covered with a fluffy layer of CoSb_3_ nanoparticles. Reproduced with permission.^[^ ^245^ ^]^ Copyright 2007, Wiley‐VCH. c) SEM image of hot‐pressed La_0.9_CoFe_3_Sb_12_/10 wt% CoSb_3_ nanoparticle sample. Reproduced with permission.^[^ ^246^ ^]^ Copyright 2008, AIP Publishing; lower frames: SEM images of NiSb nanoparticles coated on pebble like bulk Ni_0.05_Mo_3_Sb_5.4_Te_1.6_ particles before hot‐press; d) 0.074 and e) 0.16 sample; f) variation of *σ* with temperature for different samples. Reproduced with permission.^[^ ^247^ ^]^ Copyright 2018, AIP Publishing LLC.

HT/ST methods have also been used in nanocomposite fabrication through a technique called nanoplating. This method is an energy‐saving route to fabricate nanocomposites with good homogeneity. The basic idea is to place pulverized (micron size) particles into the ST/HT environment and allow the nanoparticles to grow on the surface of larger bulk particles. After consolidation (SPS or hot‐pressing) the nanoparticles would remain on the boundaries of bulk particle, achieving more homogeneous nanocomposites. During the coating process, bulk particles remain unaffected. Different amounts of nanoparticles can be coated by changing the quantity of precursors of nanoparticles. In a study, bulk CoSb3 particles were coated with nano‐CoSb3.^[^ ^245^ ^]^ The enhanced interface aided in scattering phonons thus lowering the *κ* (**Figure** **22**a,b). A similar technique was employed to grow a layer of CoSb_3_ nanoparticles on the surface of La_0.9_CoFe_3_Sb_12_ bulk matrix grains and hot‐pressed.^[^ ^246^ ^]^ Because of the reduced *κ*_l_, a *zT* value of 0.5 was attained for a sample (with 5 wt% of CoSb_3_ nanoparticles) at 725 K, which is 15% improvement over the *zT* of a nanoparticle‐free sample (Figure 22c).

Likewise, bulk Ni_0.05_Mo_3_Sb_5.4_Te_1.6_ particles were coated with different volume fractions of NiSb nanoparticles (60–80 nm) via ST coating.^[^ ^247^ ^]^ It was observed that even for a small fraction of NiSb nanoparticles (0.16), the *σ* increased considerably from 1322/Ω‐cm to 2300 Ω‐cm at 300 K due to the formation of a percolation network (Figure 22f).

##### Summary: Hydro/Solvothermal and MW‐Assisted Liquid Phase Synthesis

From the above review, it is clear that, using ST/HT processing, many inorganic materials may be produced at temperatures much lower than those required by typical solid‐state processes; it is a single‐step process. It provides easy and precise control over the size, composition, shape distribution, defect control (charge carrier concentration), core–shell structures, and crystal imperfections: dislocations, local lattice distortions, good crystallinity of the final product via adjusting the temperature, reaction time, solvent types, surfactant, and precursors etc. and the end‐products do not require postannealing treatment.

In many publications which mention the use of domestic MW ovens, information on maximum power and the type of MW irradiation (pulsed/continuous) is missing. The sample temperature is also not reported, probably due to practical difficulty in assessing it, which does not portend well for reproducibility. Therefore, well‐formulated protocols and strict adherence to them are vital; otherwise, a poor connection from theoretical chemical synthesis to technical engineering sciences may ensue.

It is yet to be explored how MW chemistry can be used in the fabrication of nanocomposites having two or more constituents, as each constituent has its own synthesis profile – instead of MW synthesis of individual constituents and mixing them later. In such studies, solvents covering the whole spectrum of MW absorptivity can be used, such as ionic liquids or EG (strongly absorbing), water, benzyl alcohol, etc. (moderately absorbing), and nonpolar alkanes or alkenes (nearly MW transparent). As mentioned earlier, the dielectric properties of many solvents vary with temperature, thus affecting the expected reaction mechanisms in negative aspects. More research in the direction of the design of mixed solvents applicable to different temperature regimes is needed. Simulated studies^[^ ^161^ ^]^ (using a single‐mode applicator) suggest that the nature of MW heating depends on many variables: sample volume, vessel material, the effect of stirring, and energy balance. Even in commercial MW applicators, the amount of power reflected varies depending on the type of vessel. The synthesis parameters from such studies are bound to lose merit unless sufficient data is provided in the reports.

#### 2.3.6 Microwave Heating Mechanisms in Solids

Recently, there is a considerable interest in the MW‐assisted solid state TE material synthesis. Accordingly, there are many reports on the TE material synthesis especially using dielectric heating (*E*‐field component). The MW absorption characteristics of the starting material in MW‐assisted synthesis determine the material properties ultimately. Consequently, understanding the mechanism of the MW interaction phenomena in relation to the physical properties of the material is very important when it comes MW‐assisted material processing. The mechanism of metals/semiconductors (with free charge carriers and ions) interaction with MWs is different from water and polar solvents and it is complex. During MW irradiation, the *E* and *H*‐field components of MW disrupt the orientation, position, and movement of dipoles, free electrons, domain walls, and electron spin. During the interaction, one or more of these events may occur. In the subsequent sections, we will discuss how different materials interact with E‐field and *H*‐field components.

##### Electric Field (*E*‐Field) Related Losses in Conductors and Semiconductors

**Figure 23:**
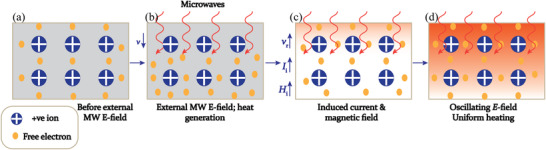
Conduction loss mechanism in metallic‐based materials and semiconductors.

*Conduction Losses*: In conductors and semiconductors, conduction loss dominates. When exposed to an external MW *E*‐field, the free charge carriers, which have freedom to move in a constrained region of the material, begin moving in the direction of the *E*‐field at a certain velocity (*v*). Since the *σ* of materials is quite high, *E*‐field gets attenuated inside the material inducing a current (*I*_i_) (**Figure** **23**c).

An induced magnetic field (*H*_i_) is developed inside the material, generating a force on moving electrons. This force pushes the conducting electrons in the reverse direction with velocity (*v*_r_) imparting them some kinetic energy. The elastic, inertial, frictional, and molecular interaction forces govern electron movement. As the electrons cannot synchronize with fast‐changing phase of the electric field, energy is dissipated in the form of heat that contributes to the volumetric and uniform heating of the material. Highly conducting materials are also heated significantly by eddy current losses due to the alternating *H*‐field. In this case, the power dissipated per unit volume, *P* (W m^−3^) into the material due to dielectric heating is given by^[^ ^248^ ^]^

(7) $$P_{E}=\omega \varepsilon_{o}{\varepsilon^{\prime \prime }}_{\mathrm{eff}}E_{\mathrm{rms}}^{2}$$

where *ω* = 2*πf*; *f*, the frequency of the incident MWs; *ε*_o_ is the permittivity of free space (*ε*_o_ = 8.854 × 10^–12^ F m^−1^); $\varepsilon_{\mathrm{eff}}^{{}^{\prime \prime }}$ is the effective dielectric loss factor ($\varepsilon_{\mathrm{eff}}^{{}^{\prime \prime }}$ = $\varepsilon_{\mathrm{polarization}}^{{}^{\prime \prime }}$+$\varepsilon_{\mathrm{conduction}}^{{}^{\prime \prime }}$). *E*_rms_is the local (at the position *x*, *y*, *z*) value of the electric field strength (V m^−1^).

##### Magnetic Field (*H*‐Field) Related Losses in Conductor, Semiconductor, and Other Materials

MWs also possess a magnetic field (*H*‐field) component, which can couple with some materials to cause heating. *H*‐field component of MW contributes greatly to MW heating of magnetic dielectric materials (e.g., ferrite), some aqueous electrolyte solutions, and certain conductive powder materials.^[^ ^178^, ^249^ ^]^ In fact, the magnetic loss contributes significantly to the MW heating of a broad range of materials: conductors, semiconductors, magnetic materials, and so on.^[^ ^176^ ^]^ Therefore, it is apparent that the *H*‐field also contributes greatly to MW heating, which can no longer be overlooked. The main mechanisms underlying MW *H*‐field heating are eddy current losses, hysteresis losses, magnetic resonance losses (including electron spin resonance), and residual losses.

**Figure 24:**
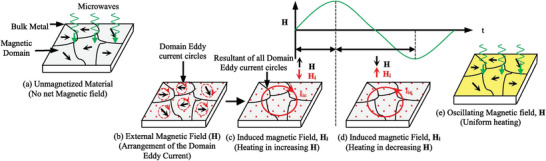
Heating mechanism via eddy current loss. Reproduced with permission.^[^ ^250^ ^]^ Copyright 2015, Elsevier Ltd.

*Eddy Current Loss*: Eddy current loss is the Joule loss due to the eddy current induced by the alternating *H*‐field and it can occur in any type of conductor. In the presence of an external *H*‐field, eddy currents are generated in the form of close loops (**Figure** **24**b) on all magnetic domains residing on the surface layer of the material. These eddy currents oppose any change in external *H*‐field. A resulting induced eddy current (*I*_ie_) for the bulk material can be viewed as the net effect of all induced eddy currents. If an external *H*‐field on the loop is increasing, the induced resultant eddy current induces a *H*‐field (*H*_i_) in the opposite direction (Figure 24c) that reduces the net *H*‐field. The *I*_ie_ then induces a *H*‐field (*H*_i_) to increase the net *H*‐field when the external *H*‐field on a loop is decreasing (Figure 24d). As a result of these changes in the direction of induced current, energy is lost in form of heat (Figure 24e). The oscillating *H*‐field repeats this mechanism rapidly and uniformly heating of the material.^[^ ^250^ ^]^

The eddy current density is expressed as *J* = *σE*, where *σ* is electric conductivity, and *E* is the *E*‐field induced by the alternating *H*‐field. Thus, from the aspect of Ohmic heating induced by an alternating *H*‐field, the eddy current loss depends on the *σ* of the material.

**Figure 25:**
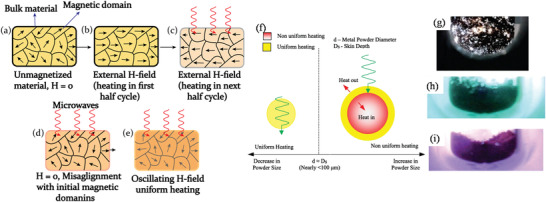
a–e) Heating mechanism via hysteresis loss; f) MW energy absorption in metallic powders. Reproduced with permission.^[^ ^250^ ^]^ Copyright 2015, Elsevier Ltd. g) Electric discharges due to MW irradiation on 618 mg, 125–180 µm Cu particles in benzene for 30 s using 300 W constant power. h,i) Discharges of Cu powder (618 mg, 180–250 mm particles, 50 W constant power) in different atmospheres: h) Ar, i) N_2_. Reproduced with permission.^[^ ^251^ ^]^ Copyright 2012, Wiley‐VCH.

*Hysteresis Loss*: Hysteresis loss occurs only in magnetic materials: ferrous materials, Fe, Ni, and a few other metals. The hysteresis loss is caused by the disruption in magnetic domain orientation caused by the external *H*‐field. This phenomenon is illustrated in **Figure** **25**. A magnetic moment is always bound to the magnetic domain due to the enormous number of spinning electrons residing in the magnetic domains. These domains are oriented within bulk magnetic materials in such a way that the material's net magnetic effect is zero (Figure 25a). When an external magnetic field (*H*) is applied to the magnetic materials, these domains try to align themselves in the direction of the applied *H*‐field (Figure 25b). If the external *H*‐field changes its direction again, the domains realign themselves with the field direction (Figure 25c).

After switching off the *H*‐field, there is no perfect alignment of the domains with their initial/original direction of magnetic domains in unmagnetized condition as shown in Figure 25d. Due to this misalignment, a part of the *H*‐field energy is converted into heat. As a result, the oscillatory magnetic field enforces a hysteresis effect which dissipates heat energy uniformly (Figure 25e) in magnetic materials as it traverses around a *B–H* hysteresis loop.^[^ ^250^ ^]^ The uniform heating during this mechanism is influenced by porosity, grain size, and impurities, as well as by the intrinsic properties of the materials.^[^ ^252^ ^]^ The energy loss per circle is the area within the hysteresis loop (*B* vs *H* curve) of the material.^[^ ^253^ ^]^

Thus, in addition to dielectric heating, other types of MW heating: magnetic loss heating, Joule heating caused by conductive losses, etc. are also present. Since the *E*‐field and *H*‐field of MWs interact with matter very differently, the heating behavior of a given sample may vary considerably. Therefore, to increase the MW power absorption within the materials, the electromagnetic field strength must be increased with a suitable MW cavity design. Apart from the above types of losses, magnetic resonance loss/residual losses contribute to the induction heating of ferrite and other magnetic materials in an alternating *H*‐field. These losses will not be discussed in this article however.

To summarize succinctly, MW *H*‐field heating, eddy current loss is the major contributing factor for MW heating of a broad range of conductors and semiconductor materials.^[^ ^178^, ^254^ ^]^ Inside ferrous magnetic materials, hysteresis loss occurs. In an alternating *H*‐field, magnetic resonance loss/residual loss contributes to the induction heating of ferrite and other magnetic materials.

Analogous to the dielectric losses in the *E*‐field, the power dissipated/unit volume into a material in the *H*‐field (expressed in W m^−3^) can be expressed as^[^ ^248^ ^]^

(8) $$P_{H}=\omega {\mu^{\prime \prime }}_{\mathrm{eff}}H_{\mathrm{rms}}^{2}$$

where, *μ*_o_ is the vacuum magnetic permeability (*μ*_o_ = 4*π* × 10^–7^ H m^−1^); $\mu_{\mathrm{eff}}^{{}^{\prime \prime }}$ depicts the imaginary part of the effective magnetic permeability ( *μ*′′_eff_ = *μ*′′_hysteresis_ + *μ*′′_eddy current_ + *μ*′′_residual_). *H*_rms_ represents the local value of the magnetic field strength (A m^−1^).

The power dissipated per unit volume, *P* (W m^−3^) into the material due to *E*‐fields (first term, Equation 7) and *H*‐field (second term, Equation 8) and can be expressed as:^[^ ^248^, ^253^ ^]^

(9) $$P=\omega \left(\varepsilon_{o}{\varepsilon^{\prime \prime }}_{\mathrm{eff}}E_{\mathrm{rms}}^{2}+\mu_{o}{\mu^{\prime \prime }}_{\mathrm{eff}}H_{\mathrm{rms}}^{2}\right)$$

In the case of non‐magnetic materials, $\mu_{\mathrm{eff}}^{{}^{\prime \prime }}$is negligible, hence power absorption contribution from *H*‐field (second term in Equation 9) can be neglected. Further, the factors $\omega E_{\mathrm{rms}}^{2}$ and $\omega H_{\mathrm{rms}}^{2}$are independent of the material properties for nonmagnetic and magnetic materials, respectively. But they are related to the specific MW cavity used at a specific power.^[^ ^250^ ^]^ As the temperature of the material rises over time, all of its characteristics are updated, impacting power absorption. Akin to dielectric tan*δ*, the magnetic loss tangent is given by

(10) $$\tan\delta_{\mu }=\frac{{\mu_{r}}^{\prime \prime }}{{\mu_{r}}^{\prime }}$$

where *μ*′_r_ is the relative magnetic constant, which is a measure of the ability of the material to store magnetic energy; *μ*′′_r_ is the relative magnetic loss factor which represents the loss of magnetic field energy.

#### 2.3.7 Microwave Penetration and Power Absorption in Solids

When the solid material is exposed to MWs, the temperature profile within that material is determined by several geometrical factors related to the design of the MW applicator. When MWs are incident perpendicularly on the surface of a material, its intensity decreases progressively inside the material in the incident direction (as in light). MWs do not permeate in all materials in a similar manner. MW penetration inside a nonmetallic material is defined in terms of penetration depth (*D*_p_), (Equation 6). Bulk metals and alloys generally reflect the MWs and MW penetration is rather shallow. In such cases, the MW penetration is commonly described by a quantity known as skin depth and is defined as^[^ ^253^, ^255^ ^]^

(11) $$Ds=\frac{1}{\sqrt{\pi \mu_{f}\nu \sigma }}=0.029{\left(\rho \lambda_{o}\right)}^{0.5}$$

where *σ* (1Ω^−1^ m^−1^) is the electrical conductivity, *ρ *(Ω m) is electrical resistivity, and *λ*_o_ (m) is the incident wavelength, *ν* is the MW frequency, and *μ*_f_ is the permeability of free space (1.257 × 10^–6^ H m^−1^). As a result, it is obvious that the amount of energy absorbed during MW irradiation is dependent on the thickness (volume) of the target material and its electromagnetic properties. The above relation implies that using low‐frequency MWs is preferable when large samples are used, but it is associated with a reward in terms of power absorbed per unit volume. When the metallic particle dimension is comparable to skin depth, the portion of metal particle surface area that couples with MW energy is enough to contribute uniform MW heating (Figure 25f). This will be discussed in Section 2.3.7.1.

Many inorganic materials are known to couple strongly to MWs. When these materials are used as one of the constituents, they act as a susceptor taking up the energy from the MW field and gets heated up very rapidly facilitating the reactions (MW‐solid state reaction). Table S4 (Supporting Information) lists of some minerals and inorganic compounds that can couple with MWs at ordinary temperatures.^[^ ^256^ ^]^ The temperatures attained by these materials along with the associated exposure times when irradiated by MWs in ordinary domestic MW ovens are also shown in the table. Most powder forms of carbon interact with MWs. Notably, the amorphous carbon powder absorbs MWs (2.45 GHz frequency) quickly, reaching 1550 K under 1 min in a household MW oven operating at 1 kW.

The MW uniform heating and energy conversion ratio of good MW absorbers can be enhanced further when they are mixed with some MW transparent materials.^[^ ^257^ ^]^ For example, the overall heating effect of 10 g pure activated carbon (AC) was lower than the 5 g AC, which was mixed with 25 g of high‐quality quartz powder (MW transparent) when they were exposed to MWs in the same spherical shape (diameter of 20 mm) and similar MW conditions. Since AC is a strong MW absorber due to its high dielectric loss,^[^ ^258^ ^]^ its power dissipation capacity is quite high, while the penetration depth is very low, only the outer portion of the AC is effectively heated by the MW and the inner portion via heat transfer. In the AC/quartz powder sample, the effective dielectric loss must have decreased. As a consequence, the heat generation was more uniform (with improved energy conversion efficiency) as compared with pure AC. Similarly, it was shown that the temperature of rGO (reduced graphene oxide with defects) can be raised to 1600 K in 100 ms using a household MW oven.^[^ ^259^ ^]^ By controlling the number of defects in rGO, MW absorption of 70% was observed. Therefore, rGO can be a good MW susceptor for many MW solid‐state reactions. As a result, effective dielectric loss and penetration depth are two critical factors in determining overall heat generation under MW irradiation.

##### Microwave Absorption Characteristics in Metallic Powders

The skin depth of bulk metallic materials is relatively shallow. However, when the metallic powder dimensions are comparable to skin depth (which is true in most cases), the portion of metal powder (surface area) that interacts with MW energy is enough to contribute to uniform MW heating (Figure 25f). As a result, the MW heating of submicron and nanoscale powders of some metals have shown good MW absorption and volumetric heating.^[^ ^260^ ^]^ For volumetric heating of metallic powders (at 2.45 GHz MW), the theoretical limit of metallic fine powder size is <100 µm. For coarser metallic particles (>100 µm), the MW heating is nonuniform and the MW absorption and heat generation is predominant up to skin depth. Heating of the rest of the metallic particles happens because of heat transfer via conduction from skin depth to the inner portion of the particle.

Using finite element analysis of the absorption of MW energy in conductive nonmagnetic spherical particles, Suzuki et al. have shown that at MW frequency of 2.45 GHz MW, energy absorption due to *H*‐field maximizes when the particle radius satisfies the conditions shown in Equation 12.^[^ ^261^ ^]^ A somewhat similar expression (Equation 13) derived using the analytical method is proposed by Porch et al.^[^ ^262^ ^]^

(12) $$R_{m}=2.5D_{S}$$

(13) $$a=2.41\;D_{S}$$

The skin depth (*D*_S_) of Cu (*σ *= 5.8 × 10^7^ Ω^−1^ m^−1^; MP ≈ 1085 °C) at 2.45 GHz frequency is 1.33 µm. Therefore, maximum MW absorption occurs when the Cu particle radius is 3.33 µm.^[^ ^261^ ^]^ Based on the analytic solution for the *E* and *H*‐field absorption of small conducting spheres (radius < wavelength of the MW wavelength), Porch and co‐workers have proposed simple principles for the optimization of MW energy absorption in conducting particles.^[^ ^262^ ^]^ According to these principles, *H*‐field absorption dominates *E*‐field absorption over a wide range of particle radii (<10 nm to >1 mm); optimum *H*‐field absorption is set by the ratio of mean particle radius (*a*) to the skin depth (*D*_S_) by the condition expressed in Equation 13.

That means for a metal (nonmagnetic) of any given *σ *and at a particular frequency, maximum *H*‐field absorption can be realized by a simple selection of the mean particle radius. For weakly conducting samples, *E*‐field absorption dominates, and peaks at

(14) $$\sigma \approx 3\;\omega \varepsilon_{o}$$

This *σ *value is fairly low (0.4 Ω^−1^ m^−1^) if the frequency is confined to 2.45 GHz and is independent of particle radius. If *H*‐field absorption were to be used instead, there is much more flexibility in satisfying the condition for maximum absorption as both *a* and *σ* can be adjusted while maintaining the condition in Equation 13. If particles of lower *σ* are chosen, then for a fixed frequency the maximum *H*‐field absorption can be obtained by an appropriate increase of *a*.

Studies have shown that certain conducting materials heat well in the *E*‐field, others in the *H*‐field, and some, like Cu, heat in both fields.^[^ ^178a^ ^]^ Ma et al. showed that the MW heating behavior of porous Cu bodies depends considerably on whether the sample is initially heated in the *E* or *H*‐field, the particle size, and on the relative density (to some extent) of the compacts. They also observed that the MW heating causes rapid changes in the absorption variables: *σ*, *ε’*, and *μ’’*.^[^ ^263^ ^]^ MW heating effect on some metallic materials is also reported by Buchelnikov et al. In this case Fe powder was effectively heated because of eddy current loss (in oscillating *H*‐field) and magnetic reversal loss (in oscillating *E*‐field). In comparison to paramagnetic Ti, diamagnetic metals Sn and Cu were heated more efficiently, whereas Au was only marginally heated. Cu‐ and Ni‐based metallic glassy powders were also moderately heated.^[^ ^264^ ^]^ Study on MW‐induced electric discharge phenomena in metal–solvent mixtures reported that a strong and brilliant white discharge was seen when 618 mg of Cu powder (125–180 µm particle size) was submerged in benzene and bombarded with 300 W of steady power for 30 s (Figure 25g). Similarly, Cu particles irradiated at 50 W MW power produced brilliant white and aquamarine blue discharges in an Ar atmosphere (Figure 25h). Cu particles of the same dimension showed bright pink discharges across the entire cross‐section of the vial when irradiated at 50 W in a N_2_ atmosphere (Figure 25i). However, discharges were less frequent than they were in Ar atmosphere.^[^ ^251^ ^]^

#### 2.3.8 Solid‐State MW Syntheses of TE Materials

As in liquid state MW synthesis, MWs as energy sources can be used in solid‐state synthesis of TE materials either by direct MW heating or via MW heating using susceptors. Due to the low skin depth of MWs in bulk metals (several μms because of MW reflection), only the surface undergoes heating.^[^ ^253^, ^265^ ^]^ However, with a reduction in grain size of these metals, the absorption of MWs dramatically improves, inducing a very high heating rate. In the following section, we touch upon a few cases where the starting materials were heated with and without susceptors and a couple of MW‐induced plasma (MIP) cases.

In the MW‐solid‐state synthesis of Sb_2_Te_3_, Sb_2_Se_3_, Bi_2_Se_3_, and Bi_2_Te_3_ via MW irradiation of elemental powders, the effects of a number of factors on the reaction outcomes have been reported.^[^ ^266^ ^]^ The influence of sample amount on phase purity was discovered to be dependent on the type of chalcogenide produced. For example, in the case of Sb_2_Te_3_, there was an optimum sample size at which very high phase purity could be achieved, while in the case of Sb_2_Se_3_ reactions, there was a minimum sample size requirement for a reaction to ensue. The MW penetration depth is mentioned as a possible factor for the link between sample amount and phase purity, and it is suggested that bigger samples are more likely to have a larger penetration depth. Furthermore, it was observed that the heat dissipation in larger samples is slower due to a smaller surface area/volume ratio. In some cases, increasing MW exposure resulted in more purity, but in other cases, the opposite trend was noticed. The position of the sample in the container was found to have a considerable effect on the outcome. For example, whether the powder/compact sat at the bottom of the tube in a vertical position or was spread along the length of the tube when the tube was positioned horizontally. This variance was explained by taking into consideration penetration depth, heat dissipation, or field nonuniformity in the MW applicator. Such findings prove that the interaction of MWs and matter is quite intricate.

MW as an energy source has been employed to synthesize high‐temperature Mg_2_Si TE material.^[^ ^267^ ^]^ The chunks of Mg and Si were dry ball‐milled, cold‐pressed, and transferred to a quartz tube which is equipped with N_2_ inlet and outlet to avoid oxidation of Mg. The MW device, consisting of a MW generator working at 2.45 GHz that is capable of delivering MW radiation with a tunable power of up to 2 kW, was used. It was found that Si can absorb MWs in the *H*‐field (TE102) while it is MW transparent to the *E*‐field (TE103). Therefore, the sample was irradiated in the TE102 mode. Additionally, ball‐milling parameters were found to affect the MW heating. Using this technique, a homogeneous nanocrystalline Mg_2_Si (size < 100 nm) was obtained utilizing 175 W MW power with a 2 min duration on a 2 h ball‐milled powder.

**Figure 26:**
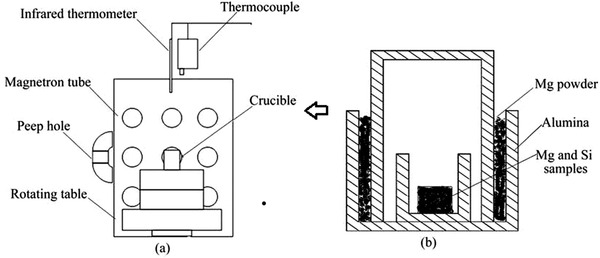
a) Schematic diagrams of MW system setup and b) crucible. Adapted with permission.^[^ ^269^ ^]^ Copyright 2011, The Nonferrous Metals Society of China, published by Elsevier).

Studies have shown that MW radiation can be used to dope Mg_2_Si with Ag, Sn, Sb, Co, and Bi to prepare *n*‐Type and *p*‐type materials.^[^ ^268^ ^]^ Similar to the aforementioned study, starting materials were first dry ball milled together (2 × 2 min, 450 rpm), and transferred to a glassy carbon crucible before the MW irradiation. An MW device operating at 2.45 GHz in TE102 mode was used and the material was densified via SPS. The maximum *zT* value for Mg_1.99_SiAg_0.01_ (*p*‐type) and Mg_2_Si_0.5875_Sn_0.4_Sb_0.0125_ (*n*‐type) was reported to be 0.35 and 0.7 at 775 K respectively. Similarly, high purity Mg_2_Si powder was obtained with excess Mg content (8%) from the stoichiometric Mg_2_Si at 853 K and 2.5 kW for 30 min.^[^ ^269^ ^]^ The powder form of the starting materials was mixed, compressed into a pellet, and placed inside the quartz tube (located at the center of the MW cavity) and irradiated with MWs. Excess Mg (8%) was placed separately as shown in **Figure** **26**b. A maximum *zT* of ≈0.13 was obtained for Mg_2_Si at 600 K. A 2.45 GHz MW applicator with an adjustable power output of 0–15 kW (with Ar supply) was employed in the synthesis.

**Figure 27:**
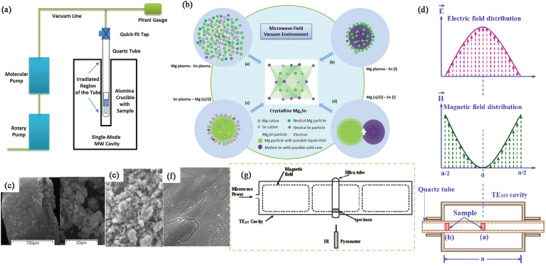
a–c) MIMP synthesis of Mg_2_Sn. a) Experimental setup for the solid‐state MW synthesis of Mg_2_Sn. b) Proposed reaction routes in the MW synthesis of Mg_2_Sn; c) outcome of 200 W for 60 s. SEM image of one condensed metallic piece (top); SEM image of the ground powders (bottom). Adapted with permission.^[^ ^270^ ^]^ Copyright 2019, ACS; d) MW field distribution within a TE10n single‐mode cavity. The sample can be positioned in (a) the maximum *E*‐field where the *H*‐field is minimum; and/or (b) the maximum *H*‐field field where the *E*‐field is minimum. The SEM images of Si_0.7_Ge_0.3_ alloy sample. e) The starting powder mixture. f) the sample heated in MW *E*‐field for 180 s. Adapted with permission.^[^ ^271^ ^]^ Copyright 2010, Elsevier. g) MW cavity in TE103 mode at 2.45 GHz: specimen is located at *H*_max_ field position. Adapted with permission.^[^ ^272^ ^]^ Copyright 2013, Taylor & Francis.

In MW synthesis, if the reagents do not couple with MWs, the use of MW‐induced plasma (MIP) (MW induced metal plasma (MIMP) in some literature) technique is very useful. The MIP is simply a substitute for a susceptor. In such reactions, plasma acts as a conduit to transfer MW energy to reagents; it also ward‐off unwanted contamination and side reactions. The MW induced metal plasma (MIMP) synthesis technique was used to prepare magnesium stannide (Mg_2_Sn, an intermetallic semiconductor) using Mg and Sn elements in powder form.^[^ ^270^ ^]^ Mg and Sn (10 µm) powders were mixed, transferred to an alumina crucible (MW transparent), placed at the bottom of a quartz tube, and subjected to vacuuming. A modified single‐mode cavity commercial MW reactor (2.45 GHz) with a tunable input power from 0 to 300 W was used for solid‐state synthesis (**Figure** **27**a). Single‐phase Mg_2_Sn was produced in 1 min duration with <200 W of incident MW radiation power. The MW heating was direct rather than using a susceptor. It was concluded that the fine Mg and Sn powders can both couple efficiently with the MW fields, heating up rapidly and generating plasma. The starting metallic materials themselves provided a source of reactive plasma. Metallic plasma production was revealed to be particularly critical for reaction completion and assisted in the improved kinetics of the process via one or more potential reaction routes to sintered Mg_2_Sn (Figure 27b).

In MW heating, a pure *E*‐field (2.45 GHz) has been used in the synthesis of bulk amorphous Si_0.7_Ge_0.3_ alloy.^[^ ^271^ ^]^ Using a pure *H*‐field yielded crystalline single‐phase alloys. High purity Si powder (particle size: ≈1 µm) and Ge powder were used as the starting materials. A quartz tube (with crucible containing sample) was inserted through the MW cavity. The MW heating experiments were carried out using a lab‐built TE103 single‐mode cavity powered by a 2.45 GHz, 2 kW MW generator (as shown in Figure 27d bottom image). The MW power used in the experiments was kept at 1 kW. The sample temperatures were monitored using an infrared pyrometer. By changing the position of the sample inside the MW single‐mode (TE10n) chamber, the sample could be exposed to either pure *H*‐field or pure *E*‐field thus providing freedom to characterize the MW‐materials interaction with different MW field excitation (Figure 27d bottom image). It was revealed that in the MW *H*‐field, the sample's temperature stayed constant (≈900 °C) even when the MW power increased (>1 kW) indicating the material is no longer absorbing the MW energy at this temperature (i.e., MW reflection is occurring) and the material is behaving like bulk metal. Further increase in MW power resulted in more MW reflection culminating in more MW cavity loss. That means the cavity becomes much warmer at high power irradiation. However, when the sample was moved to *E*‐field and applied the same MW power ≈1 kW, the temperature of the sample increased (1450–1500 °C) and the original cylindrical sample became ball‐like, revealing some melt of the sample had occurred (Figure 27f). At this stage, the sample became amorphous Si_0.7_Ge_0.3_. This sample exhibited *p*‐type conduction and the measured *S* was reported to be ≈270 µV K^−1^ at 170 °C.

**Figure 28:**
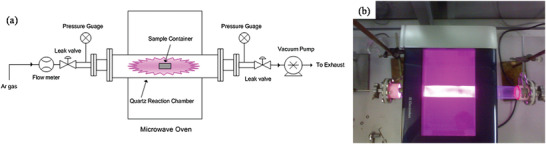
Apparatus used for MW plasma synthesis. Adapted with permission.^[^ ^273^ ^]^ Copyright 2013, Elsevier.

In another report,^[^ ^272^ ^]^ starting materials Si and Ge (75 wt% Si + 25 wt% Ge) were ball‐milled (plastic balls in a Wig‐bug mixer) for 10 min. The mixed powders were then packed in alumina cylinder with open ends. This cylindrical crucible/pellet was transferred to the fused silica tube and placed at the maximum *H*‐field region. Figure (Figure 27g) shows the *H*‐field distribution in a TE103 MW cavity. The input power of 500–800 W (corresponding to 900 °C) was maintained for 5 min to get SiGe alloy. Sitthichai et al., prepared Zn_4_Sb_3_ nanocrystals by heating Zn and Sb powders (in an quartz ampoule) for 5 min using custom built 900 W MW apparatus.^[^ ^273^ ^]^ No susceptor was used. Zn_4_Sb_3_/ZnSb composites were also synthesized using MW plasma in 10 min. During MW heating purple plasma was observed (**Figure** **28**). The energy gap of Zn_4_Sb_3_ was measured to be 1.22 eV.

A series of Se and Te doped skutterudite Co_4_Sb_11.9‐_ *_x_* Te*_x_* Se_0.1_ (*x* = 0.2, 0.3, 0.4, 0.5, 0.6) was prepared by MW synthesis (5 min, 700 W) and densified by SPS (5 min, 903 K).^[^ ^274^ ^]^ Mixed powders of Co (≈48 µm), Sb (≈20 µm), Te (≈75 µm), and Se (≈75 µm) were pelletized, transferred to a quartz tube, vacuumed, sealed, and exposed to MWs. The highest *zT* of 0.81 was observed from Co_4_Sb_11.5_Te_0.4_Se_0.1_ at 773 K, in addition to *κ* of 2.8 W/m‐K. Te‐doped and Ti‐filled skutterudite phases Ti*_x_* Co_4_Sb_11.5_Te_0.5_ were prepared by a combination of MW heating and SPS.^[^ ^275^ ^]^ The mixed starting metal powders of Ti, Co, Sb, and Te were compacted into buttons, transferred to quartz tubes and the sealed tubes were heated in a household MW oven for 5 min. The resulting ingots were pulverized and SPSed at 903 K for 5 min. The Ti_0.3_Co_4_Sb_11.5_Te_0.5_ bulk had the smallest and uniform grain size (≈2 µm), showed the highest *zT* of 0.60 at 773 K, and a *κ* of 3.72 W m^−1^ K^−1^.

Similarly, a series of Co_1‐_ *_x_* Fe*_x_* Sb_3_ was prepared using MW‐assisted method and consolidated via SPS.^[^ ^276^ ^]^ The starting materials: Co, Fe, and Sb were homogeneously mixed using a ball milling. The obtained powders were pelletized, sealed in quartz tubes, buried in a powdered CuO bed in a MW oven, and heated for up to 14 min at MW power levels of 350–1700 W. The obtained product was pulverized and consolidated via SPS (600 °C for 15 min, 50 MPa). The material with a nominal composition of Co_0.8_Fe_0.2_Sb_3_ showed a *zT* of 0.33 at 700 K. Reference CoSb_3_ sample and 0.1 vol% nano‐SiC composited CoSb_3_ sample were fabricated using mechanical alloying and subsequent fast MW sintering.^[^ ^277^ ^]^ At 650 K, the highest *zT* values for CoSb_3_ and CoSb_3_/SiC composites were 0.08 and 0.15, respectively. The enhanced *zT* in CoSb_3_/SiC was ascribed to improved electrical properties at high temperatures.

In a report on MgAgSb/Ag_3_Sb composite fabrication via the MW‐assisted process and subsequent SPS,^[^ ^278^ ^]^ elemental Mg strip, Ag, and Sb powder were mixed, cold‐pressed into a pellet, and then transferred to a graphite crucible, which was embedded in SiC powder (as a susceptor). The sample was heated at 1273 K for half one hour in the MW furnace with Ar flow. The obtained product was ground into powder, ball‐milled under Ar and sintered by SPS (723 K, 60 MPa, 10 min). The palette was then sealed in a quartz tube (<5 × 10^–3^ bar) and annealed at 543 K for two weeks. This MW process did not yield a single‐phase *α*‐MgAgSb compound as small amount of Ag_3_Sb still present as the second phase. Due to a maximum PF of ≈2000 µW m^−1^ K^–2^, *κ*_l_ reduction ≈0.70 W m^−1^ K^−1^ (due to many stacking faults and edge dislocations), *zT* culminated in ≈0.76 at 548 K.

MnO_2_ is a low bandgap (1.33 eV for *α*‐MnO_2_) semiconductor; it is green material, and inexpensive. Because of its high polymorphism and structural flexibility, its powders are widely employed in catalysis, batteries, and capacitors.^[^ ^279^ ^]^ Due to the presence of numerous crystal defects during the synthesis of the sample, MnO_2_ usually shows *n*‐type conduction. Bulk and thin‐film MnO_2_ shows *S* of ≈300 µV K^−1^, and *σ* of ≈10 Ω^−1^ cm^−1^.^[^ ^279^ ^]^ Very high *S* of ≈20 000 µV K^−1^ was observed in a ball‐milled MnO_2_ nanopowder.^[^ ^279^ ^]^ MnO_2_ NWs (50 nm in diameter and 500 nm long) anchored on the surface of graphite were prepared using MW solid‐state approach (800 W, 60 s).^[^ ^280^ ^]^ The graphite or PPy granules (200 to 300 nm) was used as the MW absorbing material^[^ ^281^ ^]^ to facilitate the decomposition of Mn(NO_3_)_2_·4H_2_O.

##### Summary: MW‐Assisted Solid‐State Synthesis

As compared to HT/ST and solution‐based synthesis, the MW‐assisted solid‐state synthesis method is not well advanced mainly due to a lack of better instrumentation. As we discussed, *E*‐field and *H*‐field coupling vary from material to material and the complexity of their interaction with different materials is not quantifiable. Although the usage of MWs as energy source in material processing goes back to 1950, its progress is very slow in thermoelectrics. Due to the constraints of skin depth in bulk metals, practically all TE material synthesis uses the powder form of the starting material to couple with MWs. This adds more steps in material processing. Moreover, the TE performance of MW‐assisted solid materials is not as good as solution‐based, hydrothermally synthesized, and MW‐assisted solution phase synthesis methods. MW heating can induce changes in the MW absorption variables of materials: *σ*, *ε’’*, *μ*’’ thus adding more complexity to the heating mechanism. A majority of the reports on MW‐solid synthesis we discussed mentioned the usage of custom‐built MW apparatus or household MW ovens. The expertise that is needed to build custom set‐ups does not come from chemists alone, as MW waveguides are mainly taught in engineering discipline. Although the particle size of TE material synthesized via MW processing is frequently reported, the nanosize must have been attributed to the size of the starting material itself rather than a typical nanocrystal growth during solution‐based synthesis. The exact temperature of the compact during MW irradiation is not easy to measure. It is yet not clear if we can have control over the amount of defect formation and stoichiometry of the desired material.

#### 2.3.9 Solution Chemical Synthesis

##### Size and Shape, Composition and Phase Control

Another very important pathway to synthesize nanophase materials is the solution‐based method wherein a stoichiometric ratio of precursors is dissolved or dispersed in a solvent, heated to a required temperature and the product is extracted either by tuning the pH or by the addition of a reductant.^[^ ^282^ ^]^ The solvent is a medium in which all chemical components, atomic or molecule, diffuse and react. The type and concentration of chemicals used (solvents, starting materials, surfactants, and redox agents), the heating profile, and the temperature at which each component is introduced into the reaction mixture are all important factors in obtaining a high purity nanophase with a high yield.

To avoid undesirable chemical reactions, aspects such as chemical selection in terms of solubility/polarity and reactivity (temperature range) of the various components must be considered. Compound purity is important to ensure reliability/repeatable synthesis of the end product. The amount and type of impurities have an impact on the major TE properties of the end product: charge carrier concentration and electronic band structure. Finally, the components used should reduce the cost of post‐synthesis purification. Since most liquid solvents evaporate/decompose above 400 °C, the use of liquid solvents limits the reaction temperature above this temperature. As solvents, ionic liquids/molten salts with a relatively wide temperature range and the ability to dissolve most ionic precursors have been investigated.^[^ ^283^ ^]^ Among the several solution‐based synthesis methods, colloidal synthesis techniques allow the produced nanoparticles to be suspended in the solution without aggregation. This feature allows growing large numbers of nanoparticles in the exact same conditions with extremely narrow size, shape, and composition distributions.^[^ ^282^, ^284^ ^]^ Colloidal methods provide the most precise control over nanoparticle size, making nanoparticles ideal for use as building blocks in bottom‐up TE nanomaterial design. Early studies on the synthesis of monodisperse nanocrystals by La Mer & Dinegar show that the creation of uniform sized (monodisperse) colloidal particles requires a transient and discontinuous nucleation phase followed by slower controlled growth on the already formed nuclei.^[^ ^285^ ^]^ Faster addition of reagents to the reaction media raises the precursor concentration above the nucleation threshold. An abrupt nucleation event inhibits supersaturation. No new nuclei will be formed as long as the utilization of feedstock by the growing colloidal nuclei does not exceed the rate of addition of precursors. The initial particle size is decided by the time over which nuclei are formed and started to grow. Nanoparticles become more uniform in size if the percentage of nanoparticles growth during nucleation period is smaller compared to the growth period. This phenomenon is called focusing of the size distribution. In many systems, a second distinct growth phase occurs and is called Ostwald ripening.^[^ ^286^ ^]^ In this phase, high surface energy of smaller nanoparticles facilitates particle disintegration and the material starts to deposit on larger particles. Ostwald ripening can be exploited to simplify the preparation of a size series.^[^ ^287^ ^]^ In this method, a portion of the reaction mixture can be removed in increment at a specific time.

**Figure 29:**
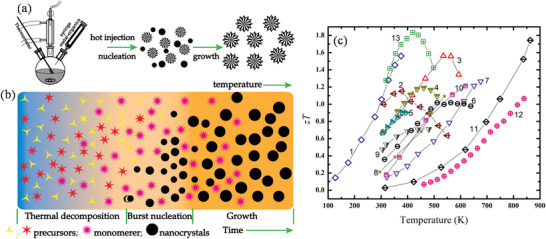
a) Cartoon depicting the stages of nucleation and growth for the preparation of monodisperse nanocrystals (NC) in hot‐injection method. b) Representation of the heating method employed in the preparation of NCs. c) *zT* of some of the solution processed TE materials of main families. **1**. *p*‐type Bi_0.5_Sb_1.5_Te_3_;^[^ ^290^ ^]^ **2**. *n*‐type K_0.06_Bi_2_Te_3.18_;^[^ ^291^ ^]^ **3**. (Ag_2_Te)_5_(Sb_2_Te_3_)_5_;^[^ ^292^ ^]^ **4**.*n*‐type Bi_2_Te_2.5_Se_0.5_;^[^ ^293^ ^]^ **5**. *p*‐type (Bi,Sb)_2_Te_3_;^[^ ^294^ ^]^ **6**. *p*‐type PbTe‐BiSbTe;^[^ ^295^ ^]^ **7**. *p*‐type Cu_3_Sb_0.88_Sn_0.10_Bi_0.02_Se_4_;^[^ ^296^ ^]^ **8**. AgBi_0.5_Sb_0.5_Se_2_;^[^ ^297^ ^]^ **9**. *n*‐type (Bi_2_Te_3_)_8.54_(Bi_2_Se_3_)_1.46_;^[^ ^298^ ^]^ **10**. n‐PbTe‐Bi_2_Te_3_;^[^ ^299^ ^]^ **11**. *n*‐type PbS‐Ag 4.4%;^[^ ^300^ ^]^ **12**. *n*‐type PbS;^[^ ^301^ ^]^ **13**. *p*‐type Bi_0.5_Sb_1.5_Te_3_.^[^ ^302^ ^]^

A very suitable condition for obtaining homogeneous size nanoparticles is nucleation and slow growth thereafter. As shown in **Figure** **29** heating or hot‐injection methods are typical methods. Figure 29a illustrates the hot injection method. In the first stage of this method, only one of the precursors (either anion or cation) is mixed with solvents and ligands in a reaction vessel; heated to a high temperature. After that, a solution containing the other precursor is rapidly introduced into the heated (hot) solution, resulting in nucleation via supersaturation. The ensuing stage of development is diffusion‐controlled growth, in which the larger particles in the solution grow at a slower rate than the smaller ones.^[^ ^288^ ^]^ As a result, the average particle size rises with time, resulting in a narrow particle size distribution. The nucleation and growth stage can be controlled by the reaction temperature, precursor concentration, and type of ligands. The higher the monomer concentration infused at the reaction temperature (i.e., hot injection), the higher the number of formed nuclei leading to smaller nanoparticles. On the other hand, a slow increase in reaction temperature generally results in larger nanoparticles. In a heating method (Figure 29b), all materials: anion and cation precursors, ligands, and solvent are placed in a reaction flask at once and heated to a target temperature. The thermal treatment decomposes the anion and cation precursors, allowing nanoparticle nucleation and growth to begin.

Colloidal stability may be achieved by using appropriate surface ligands.^[^ ^289^ ^]^ Particle aggregation and sedimentation disturb the colloidal stability. Colloidal stability in non‐polar solvents is usually enforced by steric repulsion of organic molecules containing both solvophobic (e.g., amines, thiols, silanes, or phosphines) and solvophilic coordinating groups (an alkyl chain). Colloids in polar liquids are electrostatically stabilized by charged surface ligands or surfactant micelles.

In solution‐based synthesis, nanoparticle size can be controlled by changing the quantity of surface ligand and binding strength. Higher surfactant concentrations and tightly bound surfactants result in smaller nanoparticles. To alter the speed of nucleation and the formation of the products, the kinetic and thermodynamic parameters can also be easily tuned.^[^ ^303^ ^]^ The nanoparticle shape is determined by the crystal structure of the material and the free energy of each facet.

This free energy can be controlled by employing a suitable surfactant and its amount. Surfactants control the shape of nanoparticles by limiting reagent transport to specific surfaces or modifying the free energy of specific facets. In addition, shape‐directing templates (organic, such as micelles) are employed to control nanoparticle dimensions.

The nanoparticle phase may be adjusted by the use of suitable precursor concentrations, surface ligands, and growth conditions. Even metastable phases which are not achieved through any other methods have been synthesized using solution‐based techniques in nanometer scale.^[^ ^284^ ^]^ This method has been used to synthesize a variety of TE particles: Bi_2_Te_3_,^[^ ^304^ ^]^ PbTe,^[^ ^305^ ^]^ CoSb_3_,^[^ ^306^ ^]^ and Si.^[^ ^307^ ^]^ On the negative side, the additives used in the reaction can induce oxides, which are difficult to remove after the reaction. Additionally, the residual surface oxides and organics can make the nanostructured materials difficult to sinter, thus adversely affecting both electrical and mechanical properties. Figure 29c presents the *zT* of some of the solution‐processed TE materials in recent years.

##### Solution‐Based Synthesis of TE Materials

Similar to HT/ST synthesis methods, solution‐based methods have an established niche in the preparation of a variety of nanostructures from various classes of TE materials, and numerous articles have been published on this method. The recent advances in solution‐based synthesis, including MW‐assisted synthesis of TE nanostructures, will be reviewed and addressed in the next part based on the classification of different types of materials.

*Bismuth‐Telluride Based Materials*: Bismuth telluride NPtlets (900 × 500 nm^2^ and thickness of 15.4 nm) have been synthesized by a wet‐chemical aniline‐assisted approach at a low temperature (100 °C).^[^ ^308^ ^]^ Bismuth nitrate pentahydrate (Bi(NO3)_3_·5H_2_O)), TeO_2_, aniline, and hydrazine (N_2_H_4_·H_2_O), EG, and HNO_3_ were used in the synthesis. During the reaction, aniline played a dual role involving the capping regent and the reductant at the same time. The growth of bismuth telluride NPtlets is reported to be related to the Bi/Te ratio of precursors. At RT, the bismuth telluride NPtlets showed a larger *S* of ‐135 µV K^−1^ than that of nanoparticles. A smaller *ρ* of 6.5 × 10^–3^ Ω m and a lower *κ* of 0.27 W m^−1^ K^−1^ were achieved for the pressed bismuth telluride nanoplatelet pellets. Using the above process Bi_2_Te_2.75_, Bi_2_Te_2.74_, Bi_2_Te_2.39_, Bi_2_Te_2.12_, and Bi_2_Te_1.35_ were prepared.

Under mild conditions, the liquid exfoliation process is a highly scalable solution‐based approach for producing highly crystalline 2D nanomaterials with high yields.^[^ ^309^ ^]^ The liquid exfoliation method has been employed in the synthesis of various 2D materials: graphene, transition metal dichalcogenides, transition metal oxides, boron nitride, phosphorene etc.^[^ ^309^ ^]^ Ding et al. have reported a low‐temperature method for creating colloidal suspensions of bismuth telluride and bismuth selenide.^[^ ^310^ ^]^ Both bismuth telluride and bismuth selenide are layered structures with van der Waals gaps, which can be chemically intercalated with small lithium ions using liquid ammonia. The lithiated materials exfoliated in water to form colloidal suspensions (with narrow particle size distributions centered around 0.01 mm) that were stable for at least 24 h before aggregated and flocculated out of solution. Chemical analysis showed that 1.38 Li ions are intercalated per Bi_2_Te_3_ unit. These materials are then deposited on substrates and annealed at temperatures as low as 100 °C for 24 h (or 300 °C for 1.5 h) to form highly *c*‐axis oriented materials. A pressed pellet of fully lithiated Bi_2_Te_3_ showed a RT *σ* of 125 Ω^−1^ cm^−1^, which is higher than the conductivity of 55 Ω^−1^ cm^−1^ measured for pristine Bi_2_Te_3_.

A surfactant‐free, high‐yielding, bottom‐up synthesis strategy was followed to prepare nanoflakes of Bi_2_Te_3_(BT) and Bi_2_Se_3_(BS).^[^ ^298^ ^]^ In the absence of surfactant, secondary NPts were observed growing vertically from larger NPts, which is attributed to the lack of surfactant covering atomic layer of Te or Se. The maximum *zT* of the nanocomposites was ≈0.7 at 400 K at a 10–15% BS composition due to elevated PF by a carrier filtering effect. The *κ*_l_ was within 0.43–0.52 W m^−1^ K^−1^. In SPSed (250 °C, 2 min) sample, the thin BT and BS nanoflakes (thickness distribution of 4–20 nm) generated a nanometer‐scale heterostructure interface and the band bending at the heterostructured interfaces gives rise to a Schottky barrier as the conduction band minimum of Bi_2_Se_3_ is 0.11 eV lower than that of Bi_2_Te_3_. Similarly, enhancement in TE performance is observed in Au nanodot (Au‐ND)/Bi_2_Te_3_ nanotube (BT‐NT) nanocomposites fabricated using solution‐based method and SPS (360 °C, 30 MPa, 2 min). Au‐NDs were synthesized following microemulsion method, and cocrystallized during bottom‐up growth of BT‐NTs. At 480 K, a *zT* value of 0.95 was observed in 2.0 mol% Au nanodot embedded Bi_2_Te_3_.^[^ ^311^ ^]^Bi_2_Te_3_ NPts have been synthesized using bismuth thiolate and tri‐*n*‐octylphosphine telluride in oleylamine.^[^ ^312^ ^]^ The thickness (≈1 nm thick) of the NPts corresponds to a single layer in Bi_2_Te_3_ crystals. Bi_2_Te_3_ nanostructured bulk materials were prepared by SPS of surfactant‐removed Bi_2_Te_3_ NPts. The sintering temperature has a considerable influence on grain size and density. A maximum *zT* of 0.62 was achieved at 400 K from a sample sintered at 523 K. The 1D/3D structured Bi_2_Te_3_/Ag‐NWs (1.5 vol% Ag‐NWs) composite was prepared by wet chemical methods.^[^ ^313^ ^]^ The Bi_2_Te_3_ nanopowder (flake‐like and rod‐like structure of ≈50 nm) was synthesized via a surfactant‐mediated HT method and AgNWs (mean diameter ≈ 50 nm; length ≈ 50 µm) were prepared through polyol reduction of silver nitrate. Both were mixed and consolidated via SPS (350 °C, 60 MPa, 6 min). At 475 K, *zT* of the Bi_2_Te_3_/1.5 vol% Ag‐NWs sample was ≈0.71.

RT colloidal synthesis of rhombohedral Bi_2_Te_3_ nanosheets (4 nm thick and 200 nm lateral size) with tunable thickness, morphology, and crystallinity has been reported.^[^ ^314^ ^]^ With increase in temperature to 170 °C, the thickness and lateral dimensions increased to 16 and 600 nm, respectively. In addition, the Bi_2_Te_3_ nanosheets obtained at 170 °C reaction temperature were of single‐crystalline contrary to polycrystalline Bi_2_Te_3_ obtained at RT. Bismuth triacetate (Bi(CH_3_COO)_3_), Te, trioctylphosphine (TOP), oleic acid (OlAc), 1‐octadecene (ODE), *n*‐hexane, (CH₃)₂CO, and EtOH were used in the synthesis. The OlAc was chosen as a capping ligand because it easily reacts with Bi salts to produce bismuth oleate and does not reduce Bi ions to elemental Bi. Bi_2_Te_3_ nanosheets were formed after the reaction between in situ formed bismuth oleate and trioctylphosphine telluride (TOP:Te) complex at temperatures as low as 20 °C in a noncoordinating organic solvent. The material grown at high temperature had 2D morphology with {0001} basal planes and lateral planes being predominantly {11$\bar{2}$0}. A dual‐source approach method (hot injection) using the reaction of Bi(NMe_2_)_3_ and Te(SiEt_3_)_2_ was followed for the synthesis of different Bi–Te phases such as Bi_2_Te, Bi_4_Te_3_ and Bi_2_Te_3_. The edge length of Bi_2_Te_3_ phase was roughly 40 nm.^[^ ^315^ ^]^

Bi_2_Te_3_ nanoparticles have been prepared using a low‐temperature wet‐chemical approach using bismuth (III) oleate and tri‐*n*‐octylphosphine telluride (TOP‐Te).^[^ ^316^ ^]^ The size and shape of the nanoparticles were tuned by adjusting the reaction time, temperature, nature of the surfactants, and solvents. Aromatic hydrocarbons: toluene and xylenes; and ethers: phenyl‐ and benzyl‐ether favored the formation of stoichiometric Bi_2_Te_3_ nanoparticles of plate‐like morphology, whereas the presence of oleylamine and 1‐dodecanethiol yielded Bi‐rich Bi_2_Te_3_ spherical nanoparticles. As‐ obtained Bi_2_Te_3_ nanoparticles exhibited *zT* ≈ 0.38 at RT. Bismuth acetate and TOPTe were thermolyzed in octadecene and oleic acid resulting in the formation of Bi_2_Te_3_ hexagonal and rod‐shaped particles.^[^ ^317^ ^]^ In this method, a mixture of bismuthacetate, oleic acid, and octadecene was heated to different temperatures for 10 min and the Te precursor was quickly injected (< 0.5 s) into this solution. The reaction temperature played a significant role on the morphology of the nanocrystals. At low reaction temperatures, 0D/1D morphologies predominated, whereas 2D morphologies predominated above 75 °C. Ju and co‐workers prepared S‐doped SnSe (SnSe_1−_ *_x_* S*_x_*) nanosheets (NSs) from bulk ingots by the intercalation of Li and subsequent exfoliation from bulk ingots.^[^ ^318^ ^]^

First, the Li ions were intercalated into individual SnSe_1−_ *_x_* S*_x_* layers during the HT process and allowed to react with water molecules leading to a rapid expansion of the individual layers, resulting in exfoliation of the SnSe_1−_ *_x_* S*_x_* nanosheets. TE property enhancement was achieved by the substitution of 20 at% of S atoms into SnSe. The porous structure in SnSe_1−_ *_x_* S*_x_* NSs was obtained through a solution‐phase chemical transformation using tartaric acid (t‐acid) and O_2_ at a reaction temperature of 503 K. A maximum *zT* of ≈0.12 (at 310 K) was achieved for the porous SnSe_0.8_S_0.2_ NSs. The enhancement in *zT* was attributed to a reduction in the *κ* (0.4 W m^−1^ K^−1^) despite the reduced *σ* and *S*.

*PbTe, PbSe, Cu_2_Se, SnSe, Cu_2_Te‐Based Materials*: Uniform PbTe_0.5_Se_0.5_ ultrathin NWs have been synthesized via solution method. The effect of the different KOH concentrations on size, phase, and morphology of the end products was investigated.^[^ ^319^ ^]^ Tellurium dioxide (TeO_2_), lead acetate trihydrate (Pb(CH_3_COOH)_2_.3H_2_O), selenous acid (H_2_SeO_3_), KOH, hydrazine hydrate (N_2_H_4_.H_2_O), PVP, EG, CH_3_COOH, ascorbic acid (C_6_H_8_O_6_) and absolute ethyl alcohol were used in the synthesis. When KOH was not added into the solution, the Te^4+^ ions were reduced to Te atoms, and grew into Te nanotubes. When the dosage of KOH reached 0.9 g and 1.8 g, the phases become Te and PbSe. The generated PbSe on the surface of the Te NWs acted as the diffusion barriers and prevented the outward diffusion of Te. With a further increase in KOH, the reaction rate increased rapidly, yielding PbTe/PbSe nanostructures. As the dosage of KOH reached even higher (4.5 g), the PbTe_0.5_Se_0.5_ uniform NWs (avg. diameter ≈ 19 nm) formed. The annealed powders were sintered by SPS (773 K, 5 min, 50 MPa). The *κ*_l_ is found to be 0.84 W m^−1^ K^−1^ and 0.66 W m^−1^ K^−1^ at 300 K and 550 K respectively.

**Figure 30:**
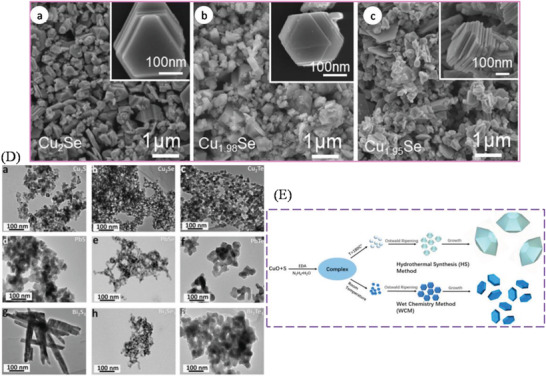
Top frames. SEM images show the morphologies of a) Cu_2_Se; b) Cu_1.98_Se; and c) Cu_1.95_Se with the insets showing high magnification SEM images. Reproduced with permission.^[^ ^63^ ^]^ Copyright 2016, Acta Materialia Inc. Published by Elsevier Ltd; Lower left frame. TEM images of Cu‐, Pb‐, and Bi‐based chalcogenide nanostructures. a) Cu_2_S; b) Cu_2_Se; c) Cu_2_Te; d) PbS; e) PbSe; f) PbTe; g) Bi_2_S_3_; h) Bi_2_Se_3_; i) Bi_2_Te_3_. Reproduced with permission.^[^ ^320^ ^]^ Copyright 2015, Elsevier. Lower right: Schematic illustration of the growth mechanism of the Cu_2_S powders synthesized by the WCM and HT method. Reproduced with permission.^[^ ^321^ ^]^ Copyright 2017, MDPI.

Highly crystalline *p*‐type *β*‐phase Cu_2_Se stacked nanosheets (lateral sizes of approximately tens of nm ‐1 µm) were prepared through heating the mixture of CuO, SeO_2_, NaOH, and PVP in EG at 230 °C for 24 h^[^ ^63^ ^]^ (**Figure** **30**a–c). After SPS (800 K, 50 MPa, 5 min), the obtained Cu_2_Se pellets exhibited an ultralow *κ* (0.2 W m^−1^ K^−1^) due to strong phonon scattering by high‐density small‐angle grain boundaries in the nanostructured material, leading to a *zT* of 1.82 at 850 K. With the increase in Cu deficiency, the carrier concentration of as‐prepared samples has increased resulting in increased *σ* and reduced *S* in both Cu_1.98_Se and Cu_1.95_Se samples, but sample Cu_1.98_Se still achieved relatively a high *zT* of 1.4 at ≈850 K.

A scalable technique was employed to synthesize a diverse spectrum of metal chalcogenide nanostructures (M*_a_* X*_b_*, M = Cu, Ag, Sn, Pb, Bi, Sb; X = S, Se, Te; *a* = 1 or 2; and *b* = 1 or 3) without the usage of surfactants.^[^ ^320^ ^]^ The main chemicals include Se powder, Te powder, NaBH_4_, Na_2_S·9H_2_O, CuCl_2_.2H_2_O, AgNO_3_, Bi(NO_3_)_3_·5H_2_O, Pb(NO_3_)_2_, SnCl_2_·2H_2_O, and SbCl_3_. In a typical experiment, NaBH_4_ was used to reduce Se powder in an aqueous solution in an inert atmosphere with constant stirring. Separately, CuCl_2_.2H_2_O was dissolved in distilled water and added to the Se precursor solution to form a black precipitate (Figure 30 frame D). This synthesis process was fast (1 h) and the yield was 90%. The synthesis of cuprous telluride and sulfide nanoparticles was similar to those of their selenide analogs, except that Se powder was replaced by Te powder and Na_2_S·9H_2_O. The nanoparticles were sintering into pellets by SPS technique. Improvement in the *zT* value was observed for some metal chalcogenide nanostructures (*zT*_max_ = 0.38 at 300 °C for SnSe; *zT*_max_ = 0.27 at 230 °C for Bi_2_Se_3_) due to the reduction in the *κ*.

Despite the fact that the precursors are the same, the morphologies and phase structures of Cu_2_S have been found to be highly dependent on reaction temperature and time. Tan et al. employed HT and wet chemical method (WCM) to prepare Cu_2_S tetradecahedron microcrystals (with monoclinic symmetry) and sheet‐like Cu_2_S nanocrystals (with hexagonal *β*‐Cu_2_S symmetry) respectively and densified via SPS (773 K, 40 MPa, 5 min).^[^ ^321^ ^]^ The *zT* of solvothermally synthesized Cu_2_S exhibited 0.38 at 573 K. Sample synthesized via WCM showed *zT* ≈ 0.62 at 773 K. As shown in Figure 30 frame E, raw CuO and S were added to N_2_H_4_·H_2_O and EDA solution to initially produce precursor Cu_2_O and S_2_^–^ and then made to react to produce Cu_2_S under different reaction conditions. For HT method when the reaction temperature was 453 K, the end product was monoclinic*α*‐Cu_2_S. After 6 h of HT treatment, monoclinic *α*‐Cu_2_S formed a tetradecahedron (several µm in size). On the other hand, in WCM method, at RT, hexagonal *β*‐Cu_2_S formed. After 12 h reaction, *β*‐Cu_2_S turned into nanosheets (edge length, 10–200 nm, thickness, 5–20 nm).

**Figure 31:**
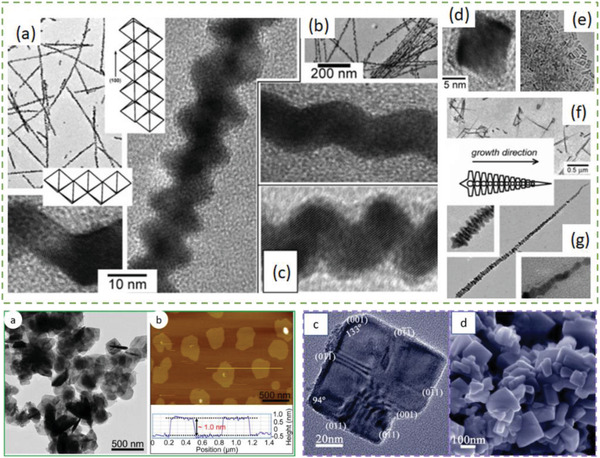
a) TEM and high‐resolution TEM images of PbSe zigzag NWs grown in the presence of HDA. The cartoons show packing of octahedral building blocks to form the NWs; b) helical NWs formed upon annealing of straight PbSe NWs in the presence of trioctylamine; c) HRTEM image of single‐crystal helical PbSe NW grown in oleic acid/HDA/trioctylamine mixture; d) octahedral PbSe nanocrystals grown in the presence of HDA and oleic acid; e) TEM images of individual nanorings; f,g) branched NWs where the length of the side arms varies along the NW as shown in cartoon. Adapted with permission.^[^ ^322^ ^]^ Copyright 2005, ACS. Lower left. SnSe nanosheets. a) TEM and b) AFM images of SnSe NSs, with their height data. Adapted with permission.^[^ ^323^ ^]^ Copyright 2013, ACS. Lower right. c) TEM images of SnSe nanostructures synthesized after 1 min. d) SEM image of SnSe NPts synthesized after 2 h. Adapted with permission.^[^ ^324^ ^]^ Copyright 2016, Wiley‐VCH.

Cho and co‐workers reported solution‐based synthesis of nearly defect‐free and highly uniform PbSe NWs (≈4 to ≈20 nm in diameter; ≈30 µm in lengths) via the oriented attachment of nanocrystal building blocks.^[^ ^322^ ^]^ During synthesis, PbSe nanocrystals are bound to each other on either {100}, {110}, or {111} facets, depending on the surfactant molecules present in the reaction mixture. In oleic acid presence only, or the cosurfactants of oleic acid and n‐tetradecylphosphonic acid (TDPA), faster growth of the {111} facet than the {100} facet occurred. Replacing TDPA with aliphatic primary amines (dodecylamine, hexadecylamine (HDA), oleylamine, etc.) resulted in the formation of octahedral PbSe nanocrystals containing eight {111} facets. This structure is due to selective blocking of the {111} facets by the binding of amines. Dipole moment aided in aligning the nanocrystals into NWs. Depending on the attachment mechanism of the octahedron nanocrystals, two types of zigzag NWs were produced (**Figure** **31**a–g, top frame). Helical NWs formed when hexadecylamine and oleic acid were used as cosurfactants in the reaction medium of trioctylamine. In addition to straight NWs, zigzag, helical, branching, and tapered NWs, single‐crystal nanorings were made in one‐pot processes by fine‐tuning the reaction conditions.

Li and co‐workers demonstrated a one‐pot synthesis method for single‐layer SnSe nanosheets in the oil phase. The 1,10‐phenanthroline was used as the morphology control agent. The obtained SnSe nanosheets had a lateral size of ≈300 nm and thickness of ≈1.0 nm (Figure 31a,b, lower left).^[^ ^323^ ^]^ Similarly, a surfactant‐free aqueous solution approach was demonstrated in the preparation of orthorhombic SnSe NPts in large quantities (>10 g).^[^ ^324^ ^]^ The reaction was carried out by injecting NaHSe solution (50 mL) into the Na_2_SnO_2_, which resulted in immediate precipitation of SnSe NPts. The reaction mechanism is: NaHSe + Na_2_SnO_2_ + H_2_O → SnSe + 3NaOH. The suspension was boiled for 2 h and the sample was dried to obtain SnSe nanoparticles. The PF of the hot‐press sintered SnSe pallet was 80% higher than the surfactant‐assisted SnSe sample. SnSe pellets (≈95% theoretical density), retaining the orthorhombic SnSe structure were obtained (Figure 31c,d, lower right).

Li and co‐workers prepared nanostructured Cu_2_Se containing spherical nanoparticles (≈100 nm) and irregular hexagonal NPts (thickness = 30–100 nm; side length = hundreds of nm to several μms), using a low‐temperature coprecipitation and a reduction method.^[^ ^325^ ^]^ As compared to the reduction synthesis technique and subsequent hot‐pressing, it was observed that the coprecipitation synthesis method and subsequent hot‐pressing is an efficient strategy to improve Cu_2_Se TE performance. The *zT* for Cu_2_Se fabricated by the coprecipitation method was 1.35 (compared to 0.97 of Cu_2_Se, fabricated by the reduction method) due to its improved PF (1.26 × 10^3^ W m^−1^ K^−2^ at 860 K). The *κ_l_* values for the two samples were ≈ 0.4–0.6 W m^−1^ K^−1^ at high temperatures.

**Figure 32:**
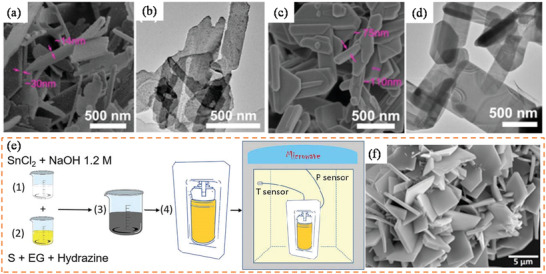
SEM and TEM images of a,b) samples Sn_1.1_ and c,d) Sn_0.85_. Adapted with permission.^[^ ^326^ ^]^ Copyright 2019, RSC. e) Steps to synthesize Sn_1‐_ *_*δ*_* S powder using the MW‐assisted method. f) SEM image of Sn_1‐_ *_*δ*_* S synthesized by MW‐HT process. Adapted with permission.^[^ ^329^ ^]^ Copyright 2020, RSC.

Studies suggest that the TE properties of polycrystalline SnS can be improved by tuning the charge carrier concentration by generating Sn vacancies. In a such study, *p*‐type SnS nanoflakes (thickness ≈ 10–30 nm; lateral length approximately several μms, **Figure** **32**a–d) have been prepared using a chemical synthesis.^[^ ^326^ ^]^ The SnS nanoflakes were synthesized via a glycol method.^[^ ^327^ ^]^ To tune the concentration of Sn vacancies (associated with hole concentration) in SnS, the molar ratio of SnCl_2_ to Na_2_S·9H_2_O was changed from 1.10 to 0.80 (in steps of 0.05). The powdered samples were then sintered using SPS. As a result of hole optimization (excess 10^19^ cm^−3^), the *σ* and PF were significantly enhanced, leading to a high *zT* ≈ 0.8 at 873 K in the Sn vacancy modulated samples Sn_0.85_ and Sn_0.8_. This *zT* value was higher than the *zT* values observed in Ag/Na‐doped sample.^[^ ^55^, ^328^ ^]^ In another study, MW‐assisted, one‐pot solution synthesis technique has been used to generate Sn vacancies in tin sulfite (Sn_0.87_S). SnCl_2_ powder and S precipitate were used as Sn and S sources, respectively.^[^ ^329^ ^]^ The synthesized Sn_1‐_ *_*δ*_* S powder had plate‐like crystals: thickness 100 nm to 500 nm; length of ≈3–4.5 µm. A commercial MW‐assisted HT system equipped with a temperature sensor and pressure controller was used to conduct the synthesis (Figure 32e,f). As‐obtained powder sample was consolidated by SPS (450 °C, 50 MPa, 5 min). Due to a high *S* (682 µV K^−1^), low *κ* (0.57 W m^−1^ K^−1^), and a relatively low *ρ* (5.6 × 10^–4^ Ω m), a *zT* ≈ 0.76 was attained at 523 K. In addition, a high *n* (2.5 × 10^18^ cm^−3^) and *μ* (13 cm^2^ V^−1^ s^−1^) at RT were measured.

**Figure 33:**
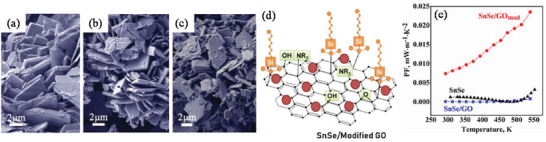
a–c) high‐magnification SEM images for SnS_0.1_Se_0.9‐_ *_x_* Te*_x_* samples with *x* = 0.02, 0.05, 0.08 respectively. Adapted with permission.^[^ ^334^ ^]^ Copyright 2019, RSC. d) Synthesis mechanism for SnSe/GO_mod_ nanocomposites. e) PF of neat SnSe, ice‐templated SnSe/GO_mod_, and SnSe/GO nanocomposites. Adapted with permission.^[^ ^341^ ^]^ Copyright 2019, ACS.

The quaternary TE chalcogenide systems: Bi_2_Te_2.7‐_ *_x_* Se_0.3_S*_x_*,^[^ ^330^ ^]^ Cu_2‐_ *_y_* S_1/3_Se_1/3_Te_1/3_,^[^ ^331^ ^]^ and PbTe–PbSe–PbS,^[^ ^332^, ^333^ ^]^ have commonly been prepared by energy‐intensive methods. Diverging from high‐temperature methods, nanostructured *p*‐type SnS_0.1_Se_0.9‐_ *_x_* Te*_x_* (*x* = 0.02, 0.05, 0.08) quaternary chalcogenides series has been synthesized via a solution‐based anion‐exchange method in an aqueous solution using SnS nano/microplates as templates (**Figure** **33**a–c).^[^ ^334^ ^]^ NaOH, SnCl_2_.2H_2_O, Na_2_S_9_H_2_O, Se powder, Te powder, and NaBH_4_ were used. For *x* = 0.02, plates with thicknesses of ≈100 nm–2 mm and lateral dimensions of 1.5–7 µm were observed. As the Te concentration increased (*x* = 0.08), the plates became more fragmented and measured ≈0.4–6 µm across (Figure 33a–c). The SnS_0.1_Se_0.9‐_ *_x_* Te*_x_* powders were SPSed (500 °C, 60 MPa, 5 min) and their TE properties were characterized (⊥ to the pressing direction). The *σ* of 5760 Ω^−1^ m^−1^ at 373 K and a PF of ≈ 0.54 mW m^−1^ K^−2^ at 423 K were achieved for the *x* = 0.02 sample. A maximum *zT* of 0.43 at 773 K was achieved by introducing 2% of Te into SnS_0.1_Se_0.9_ (SnS_0.1_Se_0.88_Te_0.02_).

When used as fillers in bulk TE materials, graphene and its closely related reduced graphene oxide (rGO) have a direct influence on the characteristics of bulk TE materials: CoSb_3_/rGO,^[^ ^335^ ^]^ Ce_0.85_Fe_3_CoSb_12_/rGO,^[^ ^336^ ^]^ PbTe/rGO,^[^ ^337^ ^]^ Cu_2_Se/graphene,^[^ ^338^ ^]^ and Cu_2‐_ *_x_* S/graphene,^[^ ^339^ ^]^ and conducting polymers.^[^ ^29^, ^340^ ^]^ Graphene oxide (GO) and modified graphene oxide (GO_mod_) have been employed in SnSe nanocomposite synthesis.^[^ ^341^ ^]^ SnSe and GO were combined using a simple aqueous solution process followed by ice templating (freeze casting). In this study, GO nanosheets were prepared by the improved Hummers’ method^[^ ^342^ ^]^ and SnSe nanoparticles (nanospheres with diameters 7–35 nm in range) were synthesized using a citric acid‐assisted solution synthesis.^[^ ^324^ ^]^ A modified ice‐templating method was followed to fabricate SnSe/GO and SnSe/GOmod nanocomposites.^[^ ^343^, ^344^ ^]^ The obtained samples were consolidated via hot‐press (500 °C, 60 MPa, 20 min). The incorporation of SnSe within the GO matrix resulted in modifications of electrical transport properties. Compared to unmodified SnSe/GO nanocomposites and pristine SnSe, the TE transport characteristics were further improved by functionalizing the GO surface to create modified nanocomposites (SnSe/GOmod) with increased PF. Functionalization of GO by reaction with octadecyltrimethoxysilane (C_21_H_46_O_3_Si) and triethylamine ((CH_3_CH_2_)_3_N) switched the conduction in SnSe from *p*‐type to *n*‐type with an appreciable *S* and a high *σ* (1257 Ω^−1^ m^−1^ at 539 K), yielding a 20‐fold increase in the PF compared to pristine SnSe prepared by the same pathway. The *κ* of the unmodified SnSe/GO composite was lower than that of the modified material (0.4 W m^−1^ K^−1^ vs 0.7 W m^−1^ K^−1^ at 550 K) possibly due to more electrically and thermally insulating nature of GO compared to the SnSe/GOmod nanocomposite (Figure 33d,e).

The hydrophilic functional groups on the surface of graphene oxide allow for good dispersion in water and aid in the creation of homogeneous SnSe and rGO composites (when reduced from GO).^[^ ^345^ ^]^ The rGO nanosheets have been used as fillers in SnSe matrix.^[^ ^345^ ^]^ In this study, a series of SnSe/(rGO)‐*x* (*x* = 0.1, 0.3, 0.5, 0.7 wt%) nanocomposites were synthesized in situ where rGO nanosheets were dispersed intimately into the SnSe matrix. Chemicals: SnCl_2_.2H_2_O, Se powder, NaOH, NaBH_4_, and GO were used for the synthesis and the obtained SnSe/rGO‐*x* (*x* = 0, 0.1, 0.3, 0.5, 0.7 wt%) powders were SPSed (550 °C, 40 MPa, 5 min). The sample SnSe/rGO‐0.3 wt% exhibited *κ*_l_ = 0.36 W m^−1^ K^−1^ at 773 K (reducing the *κ*_l_ of SnSe by 33%) to yield a maximum *zT* of 0.91 at 823 K which is ≈150% of the value obtained for unmodified SnSe.

CuAgSe is a ternary selenide, with a superionic property due to the mobility of both Cu^+^ and Ag^+^ ions at high temperatures; exceptionally high *μ*_e_ at low temperatures; *zT* of 0.15 at RT and a *zT* of 0.95 at 623 K have been reported in bulk CuAgSe.^[^ ^346^, ^347^ ^]^ Surfactant‐free CuAgSe nanoparticles were synthesized on a large scale within 30 min with a good yield (> 90%) using Cu(NO_3_)_2_, AgNO_3_, and Se precursors in an ambient temperature in an aqueous system^[^ ^348^ ^]^ and densified by SPS (430 °C, 65 MPa, 10 min). The CuAgSe nanoparticle pellet exhibited metallic characteristics below 60 K and changed to *n*‐type semiconducting nature with increasing temperature (due to changes in the crystal structure, i.e., from the pure tetragonal phase into a mixture of tetragonal and orthorhombic phases), and *p*‐type semiconductor behavior above 480 K. The CuAgSe pellet displayed a *zT* of 0.42 at 323 K and 0.9 at 623 K and exhibited excellent cycling stability.

Ecofriendly MgAgSb (MAS) is a promising candidate for low‐mid temperature applications.^[^ ^349^ ^]^ MgAgSb is a half‐Heusler alloy and manifests in three different forms: *γ*‐MgAgSb of high‐temperature cubic phase (> ≈633 K), the Cu_2_Sb‐type *β*‐MgAgSb intermediate tetragonal phase (573–633 K), and tetragonal *α*‐MgAgSb (*α*‐MAS) RT phase.^[^ ^349^, ^350^ ^]^ With respect to TE performance, *α*‐MAS is superior to the other two phases. However, fabrication of single‐phase *α*‐MAS compound is a strenuous task. At present, high‐temperature melting^[^ ^351^ ^]^ and two‐step mechanical alloying,^[^ ^352^, ^353^ ^]^(MA) methods are in use to fabricate *α*‐MgAgSb. To improve the TE properties of *α*‐MAS, *α*‐MgAgSb/SnTe (nanocrystal) composite has been prepared.^[^ ^350^ ^]^ A ST technique was used to synthesize SnTe nanoparticles, which were then integrated into a MW‐produced *α*‐MAS matrix. To prepare MgAg_0.97_Sb, elements in nominal composition were mixed, made into disks by cold pressing and irradiated with MWs (with SiC as susceptor). The obtained ingot was crushed and pulverized to obtain MgAg_0.97_Sb powders. Separately, SnTe nanocrystals (25 nm) were obtained using Te and SnCl_2_ as Te source and Sn sources in a ST process (150 °C for 12 h). SnTe nanocrystal was then mixed with MgAg_0.97_Sb in a proper ratio, ball‐milled, and sintered to obtain *α*‐MAS + *x* at% SnTe (*x* = 1–4) series. Due to significantly enhanced PF in *α*‐MAS/SnTe (3 at% SnTe) composite, the *zT* ≈ 1.0 (increase by ≈53% compared with pristine *α*‐MAS) was observed at 548 K.

The PbTe–PtTe_2_ multiphased nanoparticles, where the individual particle is composed of both PbTe and PtTe_2_ phases were prepared using chemical synthesis.^[^ ^354^ ^]^ The adjustable ratio of PbTe: PtTe_2_ allowed the optimization of charge carrier concentration to improve the *σ* to above 10^5^Ω^−1^ m^−1^ and the PF was improved by more than two orders of magnitude in sample X_PbTe_ = 0.67 as compared to that of pure PbTe nanocrystals. The molar ratio between these two phases: PbTe:PtTe_2_ was tuned by varying the precursor ratio. Each nanoparticle was found to be a cluster composed of fine grains of PbTe and PtTe_2_ (size of 4–12 nm).

PbTe cubic nanoparticles (20–70 nm) have been synthesized by using Pb(NO_3_)_2_ and Na_2_TeO_3_ as the precursors, and NaBH_4_ as the reducing agent.^[^ ^355^ ^]^ In different crystallization stages, cubic nanoparticles (20–70 nm) appeared due to competition between crystal face energy and surface energy. In the initial stage, globular nanoparticles formed due to the minimum surface energy needed for spheres. As crystallization continued, high‐energy atoms diffuse into the lower energy location for forming the polyhedron crystal. With a further increase in reaction time, the crystal faces with higher energy stopped growing, and crystal faces with lower energy start to grow resulting in cubic morphology. Lu et al. demonstrated the synthesis and self‐assembly of both spherical (diameter of ≈8 ± 1.6 nm) and cubic PbTe nanocrystals (avg. size of ≈14 ± 1.2 nm) using an high‐temperature solution‐phase synthesis (HTSPS) approach.^[^ ^356^ ^]^

PbTe hollow nanotubes (few millimeters long) were synthesized through Ag_2_Te–PbTe nanocomposites by tuning the Ag‐to‐Pb atomic ratio.^[^ ^357^ ^]^ Through a combinational technique of electrospinning, electrodeposition, and cation exchange reactions, where Ag‐to‐Pb ratio was controlled in the composites during the topotactic transformation from Ag_2_Te nanotubes to PbTe nanotubes. The electro‐spun Ag nanofibers (60 ± 14 nm in diameter) with an ultralong aspect ratio of 10 000 were transformed into pure crystalline PbTe nanotubes via synthesis of Ag_2_Te nanotubes by Te electrodeposition and by cation exchange reaction from Ag^+^ cations to Pb^2+^ cations. This exchange took place on the backbone‐like template of the Te matrix, which controls the morphology of resultant PbTe nanotubes. The crystallinity, structural morphology, and compositions of 1D nanomaterials were achieved by topotactic reactions and by tuning the Ag atoms in Ag_2_Te‐to‐PbTe transformation during cationic exchange reaction. TE properties showed that the highest *S* of 433 µV K^−1^ of 1D nanostructure of PbTe‐based Ag*_x_* Te*_y_*‐PbTe composites with 30% Ag in PbTe and the highest PF of 0.567 µW m^−1^ K^−2^ at RT with pure PbTe nanotubes.

It is worth noting that the toxic and hazardous reducing agents: N_2_H_4_, NaBH_4_, etc. are extensively used in HT and solution methods. To overcome this problem, nanostructured Bi_2_Te_3_ has been synthesized with 100% yield by a chemical reduction method in an aqueous TeO_3_^2–^ solution using mild and safe ascorbic acid as a reducing agent instead of toxic reducing agent commonly used.^[^ ^358^ ^]^ Ascorbic acid functions as a capping agent as well as a reducing agent. It was revealed that TeO_3_^2–^ converts to metal Te via easily reducible Te species with ascorbic acid [TeO_2_(C_6_H_6_O_6_)]^2–^, and subsequently metal Te triggers the reduction of Bi species, culminating in Bi_2_Te_3_.

*Solution‐Based Synthesis: Summary*: Solution‐based methods have widespread acceptance and are going to retain their niche in the low‐temperature synthesis of nanophases. A plethora of literature has proved nanostructures decide the TE performance. In that direction, the solution‐based method can be very beneficial in tuning the TE performance of materials via particle size and morphology control. As we discussed the results of many studies, solution‐based synthesis is very advantageous for nanocrystal, nanocomposite synthesis with good TE properties. Solution‐based techniques provide better control over the property manipulation and functionalization of carbon allotropies (CNT, graphene, rGO, C_60_) aiding TE nanocomposites with desired properties. Also, it paves the way for doping, defect engineering, phase control, and grain boundary manipulation. Solution‐based techniques are safe and much easier to handle. Reagents can be added to solvents very easily at the right moment as the reaction progress (e.g., hot injection) to obtain p‐ or *n*‐type materials. When it comes to the scalability, solution‐based techniques fare better compared to ST methods.

On the negative side, solution‐processed products need purification that requires large amount of reagents, many of them are harmful to the environment. Therefore, a combination of greener reagents and physical/chemical techniques are needed for postsynthesis purification. Further, in many cases, high‐temperature treatment (>400 °C) is necessary to remove organic capping agents or surfactants anchored on the surface of nanostructures. The insulating remnants of organic residue left behind by incomplete removal cause the material density to change and could affect the PF of the material. For example, the surfactants PVP and oleyamine used in the synthesis usually contain a long chain of carbon and are detrimental to *σ*. To remove such surfactants, ligand exchange with short‐chain counterpart (N_2_H_4_, SHCH_2_CH_2_SH, and NH_2_CH_2_CH_2_NH_2_) or calcination is commonly used. ILs not only serve as heat‐absorbing solvents but also as nanoparticle stabilizer and shape controller.^[^ ^205^ ^]^ They provide a shield to prevent nanoparticle agglomeration and particle growth which may arise due to electrostatic charge.^[^ ^359^ ^]^ Further, ILs can easily be removed from the final product using a simple treatment thus proving clean impurity‐free particle surfaces as some studies claim.^[^ ^205^, ^207^ ^]^ Table S5 (Supporting Information) shows the material types, synthesis/consolidation methods, morphologies, dimensions, and figure‐of‐merit/temperature of recently reported TE nanomaterials and composites from different classes.

##### Powder‐Based Processing

**Figure 34:**
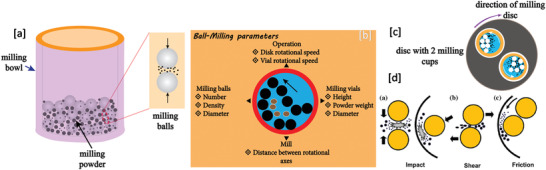
a) BM vial with balls and powder sample. Coarse powder or chunks are pulverized to form nanopowder; b) variables in BM; c) Planetary BM machine with 4 milling vial; d) main forces acting on powder particles during milling. Adapted with permission.^[^ ^364^ ^]^ Copyright 2012, Springer Science Business Media.

*Ball Milling*: Powder processing by ball‐milling (BM) is considered as green as it is energy efficient and no toxic process control agent (PCA) is needed in many cases.^[^ ^360^ ^]^ There are several types of BM machines, among them planetary, ultrasound, drum, and cryogenic mills are commonly used. However, the operating principles of these techniques are almost the same. There are three main applications of BM in thermoelectrics: producing fine‐grained or nanostructured powders,^[^ ^30^, ^105^, ^107^, ^360^, ^361^ ^]^ synthesizing compounds and alloys^[^ ^360^, ^362^ ^]^ and intimate mixing of composites.^[^ ^363^ ^]^ Due to the complex motion of the grinding media, the number of variables involved in determining the energetics of a high‐energy BM is very large^[^ ^364^ ^]^ Among them, size and number of the milling balls, medium of the jar (ZrO_2_, WO_2,_ or steel), ball‐to‐powder ratio, milling speed (rpm), milling atmosphere, number of pause intervals and their duration and types of PCAs (wet milling) (**Figure** **34**b). The mechanism of nanopowder formation is illustrated in Figure 34c,d.

BM is suitable for materials of all degrees of hardness since a broad selection of grinding pots and balls is available. However, the synthesized material may get contaminated as a result of PCA decomposition. Therefore, caution must be exercised while selecting PCA in wet grinding.

Rowe and Shukla demonstrated that the TE properties of Si_80_Ge_20_ can be improved by grain size reduction. They were able to reduce the *κ*_l_ by 28% by milling the bulk powder to less than 5 µm.^[^ ^105a^ ^]^ This grain size approach has been utilized to enhance the TE characteristics of materials of various types.

**Figure 35:**
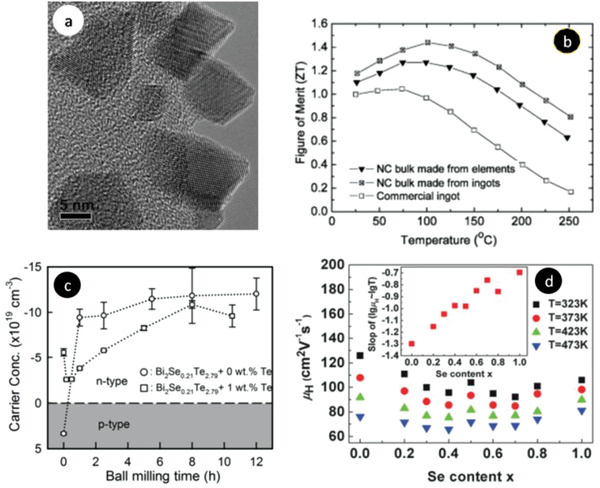
*
p *‐type BST alloy. a) HRTEM image of the mechanically alloyed nanopowders from elements; b) variation of *zT* in different samples. Adapted with permission.^[^ ^361a^ ^]^ Copyright 2008, ACS. c) Plot of *n* versus BM time for the cold‐pressed Bi_2_(Se,Te)_3_ prepared from the Bi_2_Se_0.21_Te_2.79_ and Bi_2_Se_0.21_Te_2.79_ + 1 wt% Te ground powders. Reproduced with permission.^[^ ^365^ ^]^ Copyright 2011, AIP Publishing. d) Changes in carrier *μ* with Se content at different temperatures. Inset shows scattering parameters presented by the slope of lg *μ*_H_ ∼ lg *T* curves for different Se contents. Reproduced with permission.^[^ ^366^ ^]^ Copyright 2015, RSC.

In nanostructured *p*‐type BST Alloys made from elemental chunks, a peak *zT* of ≈1.3 (in 75–100 °C range) has been achieved.^[^ ^361a^ ^]^ In this synthesis method, elemental chunks of Bi, Sb, and Te were made into alloy nanopowders using BM first and then hot‐pressed them into dense bulk samples eliminating the ingot formation step. **Figure** **35**a shows the HRTEM image of the mechanically alloyed BST sample. Figure 35b shows the transport properties of three types of samples. Similarly, Bi_2_Te_3_ samples have been prepared by high‐energy BM and SPS.^[^ ^367^ ^]^ In this study, vertical directional solidification (VDS) was used to grow *n*‐type Bi_2_Te_3_ bulk and then ball milled. The duration of BM and SPS played a major role in controlling the grain boundary density of Bi_2_Te_3_ nanoparticles. A peak value of *zT* ≈ 1.22 at the temperature of 473 K has been achieved for 8 h BM sample. The elevated *zT* value was attributed to decrease in *κ*.

In SPS process, under high pressure and temperature, mass transfer facilitates fusion of particles. It is shown that in SPSed *p*‐type bulk Bi*_x_* Sb_2‐_ *_x_* Te_3_ compounds, the electric current can interact with crystal defects as well.^[^ ^368^ ^]^ In one such study on *n*‐type Bi_2_(Se,Te)_3_, the effect of ball milling, thermal annealing, and electrical stressing on the growth of defect population and the changes of electrical and thermal transport properties of Bi_2_(Se,Te)_3_ are investigated.^[^ ^365^ ^]^ It was observed that the crystal defects generated during BM were mainly Te vacancies in the pressed Bi_2_(Se,Te)_3_. With increased BM times, the pressed Bi_2_(Se,Te)_3_ showed increased electron concentration and decreased Hall mobility (Figure 35c). Due to the strong interaction between ionized defects and flowing electrons, ionized crystal defects vanished when the Bi_2_(Se, Te)_3_ was subjected to electrical stressing (average current density of 100–200 A cm^−2^). The electrically stressed Bi_2_(Se,Te)_3_ samples showed lower carrier concentration and higher *μ* when compared to the thermally annealed ones. Similar study reveals that, in *n*‐type Bi_2_Te_3−_ *_x_* Se*_x_* materials processed by MA and SPS, the concentration of point defects can be changed by varying Se content from 0 to 1.^[^ ^366^ ^]^ The point defects and their interaction not only affected the *n* and *μ* (Figure 35d), but also enhanced the phonon scattering. A maximum *zT* ≈ 0.82 at 473 K was observed for a sample with optimized composition Bi_2_Te_2.2_Se_0.8_, which is different from a conventionally obtained ingot of composition, Bi_2_Te_2.7_Se_0.3_, demonstrating MA and SPS can shift the optimal content from conventional *x* = 0.3 to *x* = 0.8 due to point defect adjustment via Se content. Ball‐milling method has been employed in Yb doping as well as in generating a high density of boundaries and defects in *α*‐MgAgSb.^[^ ^369^ ^]^ The Yb doping decreased the *κ*_l_ because of the strong point‐defect phonon scattering. Tuning the carrier concentration via Yb doping also leads to enhanced PF. As a result, a peak *zT* of ≈ 1.4 at 550 K and avg. *zT* of ≈1.2 from 300 K to 550 K has been achieved.

Fan et al. fabricated *n*‐type Bi_2_Te_3_ nanocomposites by blending Bi_2_Te_3_ NPts of hexagonal morphology and mechanically alloyed Bi_2_Te_3_ powder.^[^ ^181a^ ^]^ First, Bi_2_Te_3_ plate‐like crystals were rapidly synthesized using a MW‐assisted wet chemical (MAWC) method. To prepare Bi_2_Te_3_ powder, elemental Bi and Te powders in an atomic ratio of Bi_2_Te_3_ were subjected to BM in a planetary ball‐mill (400 rpm for 6 h in Ar atmosphere). A stainless steel jar and balls were used and the weight ratio of ball/powder was 13:1. Both powders were homogeneously blended and consolidated by plasma‐activated sintering. When the content of the doped NPts was 15 wt%, the *ρ* and the *S* of the Bi_2_Te_3_ nanocomposites increased and the *κ_l_* of the nanocomposites decreased by 18% compared with that of undoped alloys. The maximum *zT* was 0.39 at 300 K.

It has been observed that Bi_2_Te_3_ ingot prepared by zone melting is always *p*‐type due to the formation of negatively charged antisite defects;^[^ ^370^ ^]^ however, the same material changes to *n*‐type when subjected to BM because of the large amounts of anion vacancies^[^ ^370^ ^]^ and donor‐like effect.^[^ ^371^ ^]^ BM and SPS differently affect the electrical properties of p‐ and *n*‐type Bi_2_Te_3_‐based materials; the *zT* increased in *p*‐type Bi_0.5_Sb_1.5_Te_3_ but decreased somewhat in *n*‐type Bi_2_Te_2.7_Se_0.3_ after BM and SPS.^[^ ^361b^ ^]^ Hall measurement indicated that BM and SPS reduced the hole concentration of *p*‐type alloys while increased the electron concentration of *n*‐type alloys. The reason was attributed to the formation of positively charged anion vacancies as well as donor‐like effects, which are induced by strong mechanical deformation. Consequently, changes in charge carrier concentration will affect the *σ *as well as the *S*. Hu et al. applied the atomic scale point defect engineering to enhance the TE properties of both *p*‐type BiSbTe and *n*‐type BiTeSe alloys.^[^ ^372^ ^]^ As a result, *zT* of ≈1.2 at 445 K and 1.3 at 380 K were obtained for *n*‐type polycrystalline Bi_2_Te_2.3_Se_0.7_ alloys and *p*‐type polycrystalline Bi_0.3_Sb_1.7_Te_3_ alloys, respectively.

Yang et al. observed that by BM the starting materials: Co and Sb (1:3 ratio) for longer than 20 h, CoSb_3_ (*δ*‐phase) can be obtained.^[^ ^373^ ^]^ They also observed that by ball milling (10 h), the annealing time could be reduced to as low as 1 h (at 700 °C). In another study, a BM technique has been used to prepare the single and double‐filled *p*‐type skutterudites (Ce_0.8_Fe_3_CoSb_12_ and Ce_0.5_Yb_0.5_Fe_3.2_5Co_0.75_Sb_12_).^[^ ^374^ ^]^ Maximum *zT* of 0.68 and 0.93 were observed for Ce_0.8_Fe_3_CoSb_12_ at 773 K and Ce_0.5_Yb_0.5_Fe_3.2_5Co_0.75_Sb_12_ at 823 K, respectively. The improvement in TE values at RT was due to a reduction in *κ* (reduced grain size during ball milling). The densified samples prepared by BM were shown to be resistant to oxidation. Double‐filled *p*‐type Ce_0.45_Nd_0.45_Fe_3.5_Co_0.5_Sb_12_ skutterudite was prepared by directly ball milling the quenched ingot and hot‐pressing (650 °C, 5 min) bypassing annealing.^[^ ^375^ ^]^ A pure skutterudite phase was observed when a BM time is >2 h. High energy BM did not immediately transform the quenched ingot into a filled‐skutterudite phase, but rather considerably aided phase formation during hot‐pressing by pulverizing the ingot into nanosized grains, reducing the distance the filler atoms had to travel. Ball‐milling longer than 3 h did not change the electronic transport considerably, except for the *σ*. Samples ball‐milled for 3 h showed appreciable PFs and peak *zTs* > 1 beyond 750 K.

Investigation on the TE properties of *n*‐type PbTe (doped with Na_2_Te (0.5–1.5 mol%)) powder that was prepared via planetary ball‐milling and hot‐pressing (773 K, 50 MPa)^[^ ^376^ ^]^ shows that Na_2_Te doping not only increased the lattice parameter but also changed the conduction type from *p*‐type to *n*‐type. Furthermore, it was observed that Na^+^ was located at the interstitial sites of PbTe and generated excess electrons, increasing the *σ*. The enhancement in the PF of PbTe doped with Na_2_Te samples was attributed to the higher *S* and *σ*. A maximum *zT* value of 0.81 at 700 K (compared to 0.03 for a PbTe sample at 700 K) was observed for *n*‐type PbTe (doped with 1 mol% Na_2_Te) due primarily to a significant increase in the PF and a reduction in *κ*. Similarly, *zT* ≈ 1.3 was obtained for *x* = 0.01 at 850 K among the *n*‐type PbSe:Al*_x_* (*x* = 0, 0.005, 0.01, 0.02, and 0.03) PbSe samples fabricated via BM and HP.^[^ ^47b^ ^]^ The increase in *zT* was ascribed to the enhancement of the *S* due to the increase of the local DOS near Fermi level by possibly Al generated resonant states in the conduction band and reduction of the *κ*_l_ (0.6–0.7 W m^−1^ K^−1^) by numerous microstructures: Pb depleted disks, small grains, nanosized pores and 10 nm subgrains generated by BM and hot‐pressing.

Likewise, the Al*_x_* PbTe (*x* = 0, 0.01, 0.02, 0.03, and 0.04) series prepared by BM and DC hot pressing^[^ ^50c^ ^]^ exhibited *S* between ‐100 and ‐200 µV K^−1^ and *σ* of (3.6–18) × 10^4^ Ω^−1^ m^−1^ at RT revealing Al is an effective donor in PbTe. Al doping increased the density of states close to the Fermi level in the conduction band increasing the average energy and the effective mass of charge carrier culminating in enhanced *S*. A *zT* of 1.2 was observed at 770 K in the Al_0.03_PbTe sample.

Si_80_Ge_20_ comprising 30–200 nm and 30–200 nm particle size distribution and prepared via BM has shown a *zT* ≈ 1.3 at 1173 K (40% increase over the bulk material) due to a large reduction in *κ*_l_ (46% over the bulk reference) despite the decrease in charge carrier *μ*.^[^ ^96c^ ^]^ A *p*‐type SiGe alloy containing a small amount of Au and B (Si_0.62_Ge_0.31_B_0.03_Au_0.04_) was prepared by BM and pulsed current sintering (PCS).^[^ ^96b^ ^]^ In the sintered sample, the precipitations of Au were observed. A large *S* of 464 µV K^−1^, *ρ* ≈ 10 mΩ cm, and *κ *of 1.5 W m^−1^ K^−1^ led to a value of *zT* ≈ 1.6 at 973 K. Phosphorus‐doped SiGe bulk was prepared via MA using a BM method and La‐Nb‐STO powder was obtained from ball‐milling of a bulk La‐Nb‐STO particles (obtained from hot‐press sintering of hydrothermally synthesized La‐Nb‐STO powder).^[^ ^381^ ^]^ These two materials were mixed and hot‐pressed to obtain La‐Nb‐STO/SiGe bulk composites. By controlling the amount of La‐Nb‐STO nanoparticles in SiGe matrix, the PF enhanced due to optimization of the electron concentration and *μ* in the composite. A *zT* of 0.91 was realized in the *n*‐type La‐Nb‐STO /SiGe bulk composite at 1000 K.

Higher manganese silicide (HMS) was obtained via wet ball milling in combination with SPS.^[^ ^382^ ^]^ A *p*‐type MnTe, with high *S* and low *κ*, was dispersed in HMS matrix to form MnTe/HMS composites. In addition, Te NWs of ≈ 35 nm in diameter were prepared via a solution‐based method and embedded in HMS to form MnTe/HMS nano/bulk structures. The addition of Te NWs led to a 38% reduction in *κ* and a slight increase in *S*. The *zT*_max_ of MnTe/HMS nano/bulk composite was boosted by ≈71% (from 0.41 to 0.70 at 823 K). Liu et al. synthesized Mg_2_Sn_0.75_Ge_0.25_ by BM and HP.^[^ ^383^ ^]^ This composition showed *zT* of 1.4 at 450 °C and peak PF of 55 µW cm^−1^ K^−2^ at 350 °C. Additionally, coherent nano inclusions were observed in the Mg_2_Sn_1‐_ *_x_* Ge*_x_* materials.

Apart from TE property enhancement, BM can be employed to enhance the mechanical properties of materials, particularly for layer structured compounds like Bi_2_Te_3_ with cleavage nature. When it comes to traditional TE device making, better mechanical qualities are advantageous. The *n*‐type Bi_2_Te_3_ embedded with *x* vol% nano‐SiC particles (*x*  =  0, 0.1, 0.5, 1.0) were fabricated by MA and SPS method.^[^ ^384^ ^]^ Compared with Bi_2_Te_3_ matrix, Bi_2_Te_3_/nano‐SiC composite showed increased *S*, decreased *σ*, reduced *κ* in the 323–523 K range and improved *zT*_max_ from 0.99 for Bi_2_Te_3_ sample to 1.04 at 423 K for 0.1 vol% SiC dispersed Bi_2_Te_3_ sample. Improvements in the mechanical properties were also observed including Vickers hardness (0.62 to 0.79 GPa for Bi_2_Te_3_/1.0 vol% SiC), fracture toughness (1.34 MPa m^1/2^ for Bi_2_Te_3_/0.1 vol% SiC), and the highest Young's modulus (42.7 GPa for Bi_2_Te_3_/0.5 vol% SiC). Similarly, Pan et al. compared the mechanical strength of commercially available *p*‐type Bi_0.5_Sb_1.5_Te_3_ and *n*‐type Bi_2_Te_2.7_Se_0.3_ ingots and mechanically alloyed + SPSed polycrystalline pellets and found that both *p*‐type and *n*‐type MA+ SPS materials have 2 to 3 times higher mechanical strength than the ingots due to microstructure refinement effect and grain boundary alignment during SPS.^[^ ^361b^ ^]^ Bi‐doped nanocrystalline Mg_2_Si (Mg_2_Si_0.975_‐B_0.025_) fabricated via BM and spark plasma‐assisted combustion synthesis exhibited increasing Vickers hardness with Bi content, i.e., 484.2 ± 15.44 Hv1 (from 339.1 ± 6.78). Mg_2_Si exhibited a fracture toughness of 2.09 ± 0.03 MPa √*m*.^[^ ^385^ ^]^ High relative density, nanocrystalline features (3.45 µm) and strain hardening due to doping all contributed to a higher hardness.

**Figure 36:**
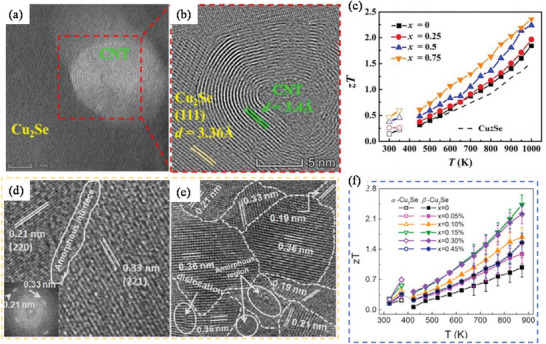
a,b) Cu_2_Se/0.7 wt% CNT; c) *zT* for the Cu_2_Se/*x* wt% CNT (*x* = 0, 0.25, 0.5, and 0.75). Adapted with permission.^[^ ^390^ ^]^ Copyright 2017, RSC. Nanostructured Cu_2_Se d) atomic‐scale image of a set of nanocrystallites showing a amorphous region at the boundary. Inset shows corresponding FFT revealing the crystallographic planes in reciprocal space and a diffused hallow ring elucidating the microstructural defects at the interface (marked with solid lines) e) atomic‐scale images of several randomly oriented nanocrystallites with presence of microstructural defects, marked with solid lines. Reproduced with permission.^[^ ^391^ ^]^ Copyright 2015, Elsevier. f) *zT* of Cu_2_Se/graphene. Reproduced with permission.^[^ ^392^ ^]^ Copyright 2018, Elsevier.

As shown in Table S6 (Supporting Information) BM has been used to process all types of TE materials. Ag‐doped SnS (SnS‐0.5% Ag) TE material fabricated by MA (450 rpm for 15 h in Ar) and SPS (933 K, 50 MPa, 5 min) has shown a *zT* of 0.6 at 923 K.^[^ ^55a^ ^]^ This Ag‐doped material found to have a high *S* of > +400 µV K^−1^; Ag doping increased the carrier concentration (> four orders of magnitude) resulting in significantly improved *σ*. The *κ* was < 0.5 W m^−1^ K^−1^ at 873 K. The CNTs were shortened and uniformly distributed within the Na‐doped SnSe matrix through a cryogenic grinding and SPSed.^[^ ^386^ ^]^ A maximum *zT* of ≈0.96 at 773 K was achieved in Na‐doped SnSe with 0.25 vol*%* CNTs. The Cu‐doped polycrystalline *p*‐type Sn_1−_ *_x_* Cu*_x_* Se material was prepared by BM and HP.^[^ ^387^ ^]^ In this case, polycrystalline Cu‐doped SnSe was prepared by traditional melting, ball‐milling, and hot‐pressing. Due to carrier optimization via Cu doping, a peak *zT* of 0.66 was observed in Sn_0.98_Cu_0.02_Se at 813 K. In LaCl_3_ doped polycrystalline SnSe synthesized by combining MA and SPS,^[^ ^388^ ^]^ the *σ* is enhanced after doping due to increased n(*μ*) and increased *S* over 300 µV K^−1^, resulting in a high PF at 750 K and a lower *κ* (< 1.0 W m^−1^ K^−1^) over the entire temperature range measured. As a result, a *zT* of 0.55 at 750 K was observed for the composition of SnSe‐1.0 wt% LaCl_3_. A *zT* of ≈1.6 at 700 °C was achieved in *β*‐Cu_2_Se made by BM and HP.^[^ ^389^ ^]^ This material showed a natural superlattice‐like structure with ordered Se and disordered Cu layers in its unit cell, resulting in a low *κ*_l_ of 0.4‐0.5 W m^−1^ K^−1^. The polycrystalline Cu_2_Se/0.7 wt% CNT (<10 nm diameter, 0.5–2 µm length, *σ *> 104 Ω^−1^ m^−1^) composite with highly dispersed molecular CNTs was fabricated by high‐energy BM and sintered via SPS. A *zT* ≈ 2.4 was obtained at 1000 K (**Figure** **36**a–c).^[^ ^390^ ^]^

Nanostructured Cu_2_Se prepared by high energy BM followed by optimized SPS has shown a *zT* of ≈ 2.1 at 973 K.^[^ ^391^ ^]^ The major factor for enhanced *zT* primarily originated from the reduction in *κ* (0.34 W m^−1^ K^−1^ at 973 K) due to the presence of different kinds of defects, nanocrystalline grain boundaries, and nano‐to‐meso scale residual porosity (Figure 36d,e). Cu_2_Se matrix/graphene NPt was prepared by intense BM followed by melting‐solidification.^[^ ^392^ ^]^ The carbon‐reinforced Cu_2_Se exhibited a *κ* of 0.4 W m^−1^ K^−1^ and a *zT* value of 2.44 at 873 K (Figure 36f). *β*‐Cu_2_Se, doped with different elements: Fe, Ni, Mn, In, Zn, or Sm were prepared through a conventional melting, BM, quenching route, followed by SPS.^[^ ^25b^ ^]^ Doping did not change the structure of Cu_2_Se. The lattice distortion and point defects aided in phonon scattering that led to a significant decrease in *κ*. Except for In‐doped sample, all the samples exhibited better thermoelectric performance as compared to undoped Cu_2_Se sample. The *zT* of the samples doped with Zn, Mn, Ni, Fe, and Sm were 1.25, 1.28, 1.51, 1.07, and 1.07 at 823 K respectively. In Cu_2−_ *_x_* Ni_x_Se, *zT* value of 1.51 was obtained at *x* = 0.0075 and 0.010.

Ge et al. fabricated a polycrystalline copper sulfide (Cu_1.8_S + Cu_1.96_S) using a combined process of BM and SPS (1173 K, 5 min).^[^ ^393^ ^]^ It was found that, beyond 973 K sintering temperature, Cu_1.8_S could decompose to Cu_1.96_S and S. Due to S volatilization, copious micropores were formed. The second phase Cu_1.96_S, enhanced the *S* and micropores reduced the *κ*. The sample, Cu_1.8_S with a second Cu_1.96_S phase showed a maximum *zT* value 0.5 at 673 K. In Cu_2‐_ *_x_* S/3D graphene heterostructured composite prepared by ball‐milling, graphene restricted the migration Cu ions, thus providing thermal stability to composite.^[^ ^339^ ^]^ Cu, S, and graphene in a required ratio were mixed, ball‐milled, and then consolidated by SPS. The highest *zT* and *PF* for sample 0.75 wt% G/Cu_2‐_ *_x_* S reached 1.56 and 1197 µW m^−1^ K^−2^ at 873 K.

**Figure 37:**
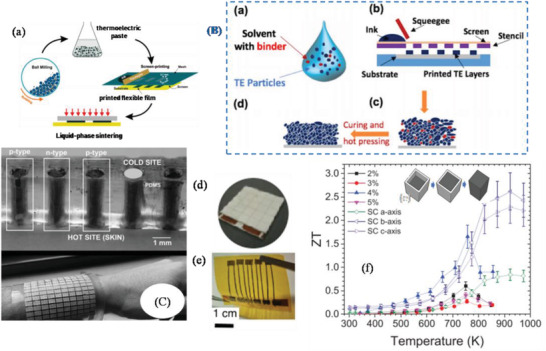
a) The method of transforming nanoparticle ink into a flexible TEG by the use of scalable and low‐cost screen printing and liquid‐phase sintering. Frame (B): Schematic illustrations of a) printable ink, b) screen printing, c) a screen‐printed TE layer, and d) a hot‐pressed layer after printing. Adapted with permission.^[^ ^377^ ^]^ Copyright 2017, Springer Nature; (C) cross section of TEG with vertical pillars filled with p‐ and *n*‐type alloys before metals deposition and wearable TEG on wrist. Reproduced with permission.^[^ ^378^ ^]^ Copyright 2017, Elsevier. d) Photograph of fabricated Zn*_x_* Sb_1‐_ *_x_* film based TE module. e) Module configuration with thermal gradient transverse to the thread direction. Reproduced with permission.^[^ ^379^ ^]^ Copyright 2019, Nature. f) *zT* of pseudo‐3D printed SnSe leg with varying amounts of binder. For comparison single crystal (SC) SnSe^[^ ^46^ ^]^ results are included. Adapted with permission.^[^ ^380^ ^]^ Copyright 2019, Wiley‐VCH.

BM is also proven to be useful in the synthesis of functional TE inks for device fabrication using ink‐jet, screen, spin‐coating, and dispenser printing. The general method to synthesize TE functional ink/paste using BM is shown in **Figure** **37**a. An inkjet‐printed flexible organic thin‐film TE device based on *p*‐ and *n*‐type poly(metal 1,1,2,2‐ethenetetrathiolate)/polymer composite (fabricated via BM) has been reported.^[^ ^394^ ^]^ To prepare ink/paste both n‐ and *p*‐type composites were prepared by BM the insoluble and infusible metal coordination polymers with other polymer solutions. This device, under the temperature difference of 25 °C, could produce TE voltage of 15 mV. Both *p*‐type BST and *n*‐type BTS TE layers screen printed on flexible glass fabrics have shown *zT* ≈ 0.65 and 0.81 respectively.^[^ ^377^ ^]^ In this method, the particles of *p*‐type‐Bi_0.5_Sb_1.5_Te_3_ and *n*‐type‐Bi_2_Te_2.7_Se_0.3_ were mixed with binder solvent (consisting of 0.45–0.60 wt% methyl cellulose as the binder, in a mixture of ethanol 60 wt% and water 40 wt%) and intimately mixed using soft ball milling; then applied to the fiberglass fabric substrate, and cured at 250–300 °C for 30 min to burn of the polymeric binders and to solidify the TE material (Figure 37, Frame B)). In another report,^[^ ^378^ ^]^ the *p*‐type Sb_2_Te_3_ and *n*‐type Bi_2_Te_3_ particles (1–2 µm) were prepared by BM high purity Bi, Sb, and Te powders. To prepare slurries, the particles were incorporated into a solution of water dispersed polystyrene nanoparticles as binder agent (100 nm) and *α*‐terpineol. A wearable TE generator (45 thermocouples) was then fabricated (Figure 37c). In a study, the screen‐printed Zn*_x_* Sb_1‐x_ film was fabricated employing BM. The a‐terpineol and DisperBYK‐110 were used as organic vehicles and dispersants, respectively. The paste was screen‐printed on SiO_2_/Si substrate after adjusting the viscosity of paste for screen printing (Figure 37d).^[^ ^395^ ^]^ In many cases, to prepare TE material for device fabrication, TE ingots were first prepared using solid‐state melting, and as‐obtained ingots were pulverized using BM and used for inks/pastes. One such device containing Bi_2_Te_3_ TE threads is shown in Figure 37e.^[^ ^379^ ^]^ In a step to prepare *p*‐type SnSe based ink, SnSe was prepared using BM (200 rpm/30 min, 30 min resting period, 60 cycles) and then mixed with a binder solution.^[^ ^380^ ^]^ An element printed from an ink (4% organic binder concentration) shows a *zT* of 1.7 at 758 K (Figure 37f).

*Summary: Ball Milling*: To conclude, ball‐milling (BM) is a valuable and versatile tool in the mass production of nanostructured TE materials, alloys, nanoparticles, and nanocomposites; it has infused some hope in economically viable method to prepare raw materials not only for conventional and wearable TE devices but also for myriads of other applications. It has been employed in almost all classes of thermoelectric materials. In many publications, milling variables are not well enunciated. Therefore, it is very important to enumerate all the variables if one decides to reproduce the results.

### 2.4 Challenges, Solutions, and Opportunities

HT/ST and MW‐assisted synthesis are beneficial for large quantities of TE nanoparticles. They are energy efficient and provide flexibility in tailoring shape, size, and chemical compositions. They also provide the opportunity for self‐doping during synthesis through reaction constituents: impurities in the precursors, solvents, and surfactants. The MW chemistry of TE materials is yet to mature to its full potential. Contrary to conventional HT/ST and MW (in presence of solvents) methods, in MW TE solid‐state synthesis the problem of removing surfactants does not arise as no surfactants are used.

For a well‐performing nanocomposite design, information about the effect of crystal size, nature of interfaces, and phase distribution on the TE characteristics is necessary. This necessitates the use of computational modeling coupled with data from experimental characterization of different systems with systematically tuned parameters. At the nanoparticle level, the charge carrier concentration depends on the type and concentration of extrinsic dopants, defects, surface chemistry (crystal atomic termination such as dangling bonds), on the concentration and type of ligands, unintentionally introduced impurities, atomic reorganization during the consolidation steps, and combinations of materials in a targeted composite. All these variables must be controlled to optimize the charge carrier concentration during material fabrication.

In bottom‐up synthesis of nanoparticles, one of the major limitations is the straightforward production of nanoparticles with the desired crystallographic texture, especially in Bi‐Sb‐Se‐Te alloys and SnSe that show highly anisotropic properties. As a result, the techniques of assembly and consolidation procedures must be improved. Development of nanoparticle assembly mediating directed attachment, crystallographic alignment of the nanoparticles during assembly, and consolidation are possible options. In this regard, the use of NPts could be very beneficial. Nanoparticle surface chemistry modification is very essential for regulating nanoparticle growth, assembly, and adjusting their functional characteristics. This information helps us in comprehending the impact of the surface on the assembly process and subsequent surface treatment (such as material diffusion).

The 0D materials of SnSe, SnTe, SnS, Bi_2_Te_3_, PbSe, and PbTe are attractive for TE applications. Using proper surfactants, precursors, and tuning the reaction conditions can result in narrow particle size distribution. This narrow distribution of particles can boost the *S* and reduce the *κ*_l_ via the classical size effect.

A solution‐based technique may be used to envision the filtering effect in nanoplating, where a material A with a suitable ionization energy (or work function) can be coated with another material B with an appropriate ionization energy (or work function) to create a filtering effect. Since the solution‐based method allows us to regulate the size/composition of the synthesized particles, if we can synthesize material A and coat it with material B in situ using a solution‐based method, then using a suitable sintering method, conducive energy barriers can be generated. During the consolidation phase, the secondary nanoprecipitates are squeezed into the voids, thus yielding high density. In other words, the secondary material can function as a glue between the bulk particles, enhancing the *σ, S*, and reducing *κ*.^[^ ^247^ ^]^ Band alignment at the interface of two species can block minority carriers more strongly than majority carriers because of different interfacial energy barriers of the valence band maximum and conduction band minimum (i.e., reducing bipolar effect). In addition, the nanoheterostructures scatter phonons of various wavelengths, lowering thermal conductivity. Using nanoheterostructures, double carrier filtering effect is also be possible.^[^ ^396^ ^]^ The quantitative determination of the exact amount of different nanostructures (and their shape) to achieve a filtering effect is still a challenge. In order to ensure the composite's long‐term stability, these barriers or secondary materials should have a low solubility in the main material. This will prevent the barrier from diffusing into the primary material.

Modulation doping can improve mobility in semiconductors. This increase in mobility is attributed to doping that is spatially inhomogeneous. More research is needed, particularly on the synthesis side, where the proper sintering temperature may be used to control the diffusion and dissolution of the minor phase into the major phase, resulting in nanocomposites with numerous doped areas that are differentially doped. 2D nanostructures synthesized (of different species in situ) via ST and solution‐based synthesis can provide good insight into modulation doping. Since Te is scarce and expensive compared to Se and S, selenide‐ and sulfide‐based nano‐heterostructures may be considered.

As we presented the results of some studies, incorporation of graphene nanosheets in TE nanoparticles during synthesis improved the TE property of the composite even though graphene is known to have a relatively high thermal conductivity. We need to think of two‐step HT/ST or solution‐based synthesis where functionalization of graphene occurs in the first step. As‐obtained graphene can be used as anchor (substrate) for TE nanomaterials. This strategy reduces the stress of residue problem to some extent, second it gives a somewhat a homogeneous material. Such homogeneous composites are easy to study using computations methods. Also, by choosing the type of TE nanoparticles on functionalized graphene, interface engineering can be manipulated. rGO can also be used as anchor point for the growth TE nanoparticles. TE/graphene composites are solution processible to prepare TE functional inks for different types of printers helping in mass fabrication of wearable TE devices.

In liquid‐like materials such as Cu_2_X (X = S, Se, Te) compounds and their alloys, the migration of Cu ions is a concern and Cu precipitates can degrade material stability. One strategy is to suppress long‐range migration of Cu ions while keeping short‐distance drift maintained by incorporating ion‐blocking barriers.^[^ ^397^ ^]^ The 3D network of graphene has proven to be useful in blocking Cu ions and the stability of Cu_2_S.^[^ ^339^ ^]^ Since graphene is easily processible, manipulation of graphene networks to improve the TE properties of Cu_2_S and other materials: Cu_2_X (X = Se, Te) needs investigation. Since Cu_2_Se and Cu_2_S can form a solid solution over the entire composition range,^[^ ^398^ ^]^ doping (polycrystalline Cu_2_Se_1‐_ *_x_* S*_x_*) using solution‐based/ST synthesis is vital since both materials contain earthly abundant materials. Cu_2−_ *_x_* Se and Cu_2−_ *_x_* Te are phase transition materials and have liquid‐like mobility of Cu. The effect of these materials on the characteristics of various TE (to generate different nano‐heterostructures) bulk needs further study.

Since C allotropies: graphene, graphite, C_60_, rGO have high loss factors, they can be used in the preparation of TE polymer/C based composites using MW‐assisted techniques. Functionalization of graphene can be achieved via MWs or ST processes and can be used to prepare different TE composites.

High *zT* values are found in single‐crystal SnSe produced by energy‐intensive techniques, but not in polycrystalline materials. The *κ* of polycrystalline SnSe is high (than the theoretical value) and its *σ* is lower than a single‐crystal SnSe (due to grain boundaries). More research is needed to see how ST and solution‐based techniques may help solve these difficulties by optimizing texturing, doping, and alloying in SnSe samples of various morphologies. As we have seen in numerous SnSe reports, vacancy engineering is one of the many benefits of solution‐based SnSe synthesis. However, in SnSe, the vacancy concentration has still not reached the optimized value. In *n*‐type SnSe, the vacancy formation energy for Se is far higher than Sn. Thus, achieving Se vacancies in pure SnSe is challenging. Therefore, substituting foreign atoms for Se sites to further reduce the formation energy of Se vacancies is a good choice and suitable candidates should be explored. On the other hand, in *p*‐type SnSe, doping with elements with a stable +2 valence state (but different atomic size than Sn) is a good alternative to reducing the vacancy formation energy, helping in a more optimized hole concentration as in the Cd‐doped SnSe we discussed.^[^ ^239^ ^]^ To further increase TE performance in polycrystalline SnSe along a particular direction, appropriate texturing is required in sintered polycrystalline SnSe that can be accomplished by HT/ST methods with a suitable morphology control. For wearable TE devices, SnSe grain optimization may be needed. Fast MW‐ST may be suitable to produce such crystals in a short time. HT/ST methods have proved that they can induce various types of nanosized local lattice imperfections, which aid in augmenting phonon scattering, as evidenced in SnSe. However, these local imperfections may also scatter charge carriers, reducing their mobility. In this case, the potential of balancing or even decoupling *σ* and *κ* values to achieve a high *zT* via appropriately tuned synthesis conditions should be investigated.

Solution‐based synthesis of PbTe is underexplored and PbTe suffers from low charge carrier concentration. Although a filtering effect was observed in thin films^[^ ^399^ ^]^ and bulk PbTe structures^[^ ^400^ ^]^ (with metallic nanoinclusions), there is no considerable improvement in the TE performance of PbTe using this phenomenon. Surfactant removal, carrier optimization, and consolidation continue to be difficult. In multiphased PbTe, how modulation doping can be accomplished needs further study.

The TE performance of 2D materials like Bi_2_Te_3_, SnS are still much lower than their conventional bulk counterparts. Precise chemical doping during the fabrication of these materials is required. This remains a challenging issue. The PF of a few layers of 2D material has been investigated, leading to a number of possible mechanisms. The exact mode of phonon and charge carrier interaction at the interfaces is not fully understood. Because existing techniques are inefficient for large‐scale synthesis, an energy‐efficient synthesis of high‐quality 2D TE materials is needed.

The SPS, HP technique, and pulsed‐electric current sintering are commonly used to densify the powders of various TE materials. SPS, or high‐temperature annealing, is particularly effective in removing organic residues and residual solvents. Depending on the particle size, shape, orientation (low angle, high angle), the mechanism of densification varies. The exact sintering mechanism (e.g., generation and propagation of sparks and plasmas) is not known at a microscopic level. A study shows the absence of plasma, sparks, or arcing during the SPS procedure.^[^ ^401^ ^]^ Understanding the evolution of nanostructure generation during consolidation is very critical.

We are unable to deliberate on GeTe and Mg_3_Sb_2_, two other high performing TE materials, considering the lack of literature on well‐established energy‐saving routes to synthesize these materials. However, we hope that the synthesis processes we reviewed, discussed, and analyzed in this review will be helpful in also developing these materials in large quantities in the near future.

### 2.5 Essence of In Situ Analysis in MW Synthesis

The area of MW‐assisted synthesis of inorganic materials has seen considerable growth in the past two decades, especially liquid‐based MW synthesis, which has retained widespread acceptance. The slow growth in solid‐state MW synthesis techniques is attributed to several reasons. First, there were no commercial laboratory MW applicators for solid‐state synthesis. Second, MWs and solid interactions appear to be very intricate. Third, the practical difficulties with accurate temperature measurement have made researchers conclude that some MW heating enhancement is due to nonthermal effects of the electric or magnetic field that are not easily verifiable.^[^ ^166^, ^402^ ^]^

The efficient coupling between MW radiation and the material depends on the type of material and the size of the particles, as we discussed in the case of MgSi_2_ and Ge–Si, attributed to skin depth (penetration depth). Some materials may exhibit good coupling even when not pulverized. When it comes to chalcogenide synthesis via MW‐solid state reaction, it is generally possible to make use of the dielectric properties of the sulfur, Se, or Te to improve the reaction processes. Chalcogens are commonly relatively poor MW absorbers. Therefore, irradiation of MW radiation on a mixture of metal powders and chalcogen aims to selectively heat the metallic component very rapidly, resulting in intense fast reactions with the chalcogen before it escapes by evaporation.^[^ ^403^ ^]^

As we have already discussed, in the MW‐solid‐state synthesis of Sb_2_Te_3_, Sb_2_Se_3_, Bi_2_Se_3_, and Bi_2_Te_3_ via MW irradiation of elemental powders, the effects of a number of variables on the reaction outcomes: sample amount on phase purity and position of the sample etc., prove that the MW–matter interaction is quite complex. Similarly, Co metal powder is known to couple with MWs at RT (Table S4 in Supporting Information).^[^ ^256a^ ^]^ However, while synthesizing In_0.2_Co_4_Sb_12_ utilizing MW radiation, a small sample (0.5 g, containing elemental powders of In, Co, and Sb) in a fused silica tube did not show any significant heating unless the tube was buried in the CuO susceptor.^[^ ^404^ ^]^ As a result, precise models for the MW field distribution and MW interaction with solids are critical for predicting reaction outcomes. Likewise, in MW liquid‐phase synthesis, various different factors decide the reaction outcome, as we have already discussed. Clearly, there is an urgency for in situ analysis of synthetic chemistry in the solid state and liquid state if we need to understand the mechanisms of chemical reactions, structure, bonding, and reaction kinetics in MW‐material interaction to a larger degree. Such in situ studies were reported by Vaucher.^[^ ^405^ ^]^

#### 2.5.1 In Situ Synchrotron X‐Ray Characterization of MW‐Assisted Synthesis

##### In Situ XRD

**Figure 38:**
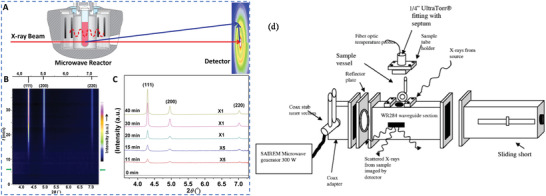
Time‐resolved HEXRD patterns recorded in the course of MW synthesis of Ag nanoparticles at 140 °C. a) Experimental setup of the in situ HEXRD; b) contour plot of the HEXRD patterns at different reaction times. The green arrows highlight the time when the temperature ramp ended and the temperature reached 140 °C. The standard XRD pattern of Ag was plotted as a reference (top sticks); c) HEXRD patterns at a number of key times. Adapted with permission.^[^ ^406^ ^]^ Copyright 2016, ACS. d) Schematic of in situ SAX and WAXS MW synthesis waveguide apparatus; Glass loop sample vessel; e) with silica lite reaction solution before and f) after reaction at 130 °C for 120 min. Reproduced with permission.^[^ ^407a^ ^]^ Copyright 2006, AIP Publishing.

High‐energy X‐rays (>300 keV) from a synchrotron beamline are a valuable tool for the structural characterization of a myriad of material types. Synchrotron sources enable the characterization of crystalline and noncrystalline materials from the microscale to the atomic scale due to their capability to produce a range of beam intensities, which the standard laboratory X‐ray sources (e.g., Cu K *α* radiation) do not endow with. Besides, synchrotron sources offer the capability to analyze materials both in situ and ex situ. In situ instrumentation is quite challenging as it demands a custom sample set‐up to simultaneously host the MW reactor and high‐energy X‐rays in addition to isolating the material of interest (i.e., the effect of X‐rays on the precursor solution and the walls of the MW reactor). Due to space constraints at the synchrotron beamline facility, the MW reactor and associated auxiliary components need to be compact and adaptable. However, such real‐time observations are very critical to understanding the influence of the MW field on reaction kinetics, intermediate phase formation, phase transition, or any transient phenomena. In one such in situ study, MW‐assisted growth of Ag nanoparticles was monitored.^[^ ^406^ ^]^ In this study, to probe the reaction kinetics, monowave 300 MW reactor (2.45 GHz) was modified to be compatible with 1 1D‐E beamline at the Advanced Photon Source (APS). The schematic illustration of the experimental setup is shown in **Figure** **38**a. Once the MW irradiation started, the beam shutter was raised to allow X‐ray beam (size ≈ 0.3 mm × 0.3 mm; with a photon energy of 70 keV) to monitor the growth of Ag nanoparticles in the reaction solution. The scattered signals were recorded at the rate of one pattern per min. It was found that the nucleation and seed growth follow a typical first‐order reaction and self‐catalytic reaction, respectively. Contrary to conventional heating (e.g., oil bath), only temperature played a significant effect on the MW synthesis of Ag nanoparticles; the concentration of the precursor AgNO_3_ and the surfactant PVP exhibited a minor effect. Further, it was revealed that the growth kinetics is different along with different crystallographic directions. The (111) direction had a higher rate constant than the (200) direction indicating that MW‐assisted Ag particle growth may be anisotropic and the surfaces of the Ag particle are terminated with {111} facets. This preferred orientation and attachment might be a significant factor in MW‐assisted synthesis rate improvement.

##### In Situ Small‐Angle X‐Ray Scattering (SAXS) and Wide‐Angle X‐Ray Scattering (WAXS)

SAXS and WAXS techniques are very common in many synchrotron laboratories to study the structural development/transformation in a reaction. These techniques can also be used for in‐situ monitoring of MW‐assisted synthesis. In SAXS only small‐angle 2*θ* values are collected by moving 2D large area plate detector far from the sample. These values are used to monitor crystallite size distributions and interactions. In WAXS, high angle 2*θ* scattering values are collected, which can be used to monitor the mechanism of long‐range ordering in materials and growth of crystalline phases.

A MW reactor was devised that could be used in in situ analysis of the role of precursor solution in silicate zeolite synthesis using X‐ray and Raman scatterings.^[^ ^407^ ^]^ As illustrated in Figure 38d, this equipment (with 2.45 GHz) included a rectangular waveguide, coaxial stub tuners, and a sliding short. The design allowed the frequency to be tuned to facilitate maximum heating in the chamber. MW power was supplied by a SAIREM type GMP 03 KS/M generator (maximum output power of 300 W), using a coaxial transition to the waveguide. Rectangular slots were carved on the side of the waveguide to allow an X‐ray beam and/or a Raman laser to pass through the waveguide at the sample position. The MWs propagated in the TE10 mode with an electric field, which was in the direction perpendicular to the slot. This study reveals that MWs enhanced the rate of zeolite crystallization irrespective of the precursor used. As SAXS data indicated, despite the rate increase, precursor concentrations had no effect on the mechanisms of zeolite formation. In situ Raman spectroscopy was also used to monitor the MW‐assisted reactions at higher temperatures in the presence of a MW field.

In situ powder X‐ray diffraction has been used to study the *β* to *α* phase transition in the ionic conductor AgI under the influence of MW radiation.^[^ ^408^ ^]^ AgI undergoes a phase transition from RT wurtzite‐type *β*‐AgI to bcc *α*‐AgI at high temperatures. It is speculated that the MW energy is able to engage with low‐energy transverse optical phonon modes in the *β*‐AgI through a multiphoton process, in contrast to interaction with rotational/vibrational modes as in conventional heating. The pace at which this energy is redistributed is slow in comparison to the rate at which energy from MWs enters the system, resulting in a nonclassical internal energy distribution. When there is enough energy in the transverse optical mode to generate enough silver ion defects or displace enough iodide ions to cause structural change, the phase transition commences. At this state, the average energy in the structure is lower than that at the point of phase transition (as compared to conventional heating), thus lowering the transition temperature. Although in‐situ synchrotron X‐ray characterization of MW‐assisted synthesis is very important, the synchrotron beamline accessibility and material costs for designing MW reactors are not within the reach of many researchers, so are in situ studies using neutrons and in situ X‐ray absorption spectroscopy.

#### 2.5.2 MW Heating Characteristics of Aqueous Electrolyte Solutions

**Figure 39:**
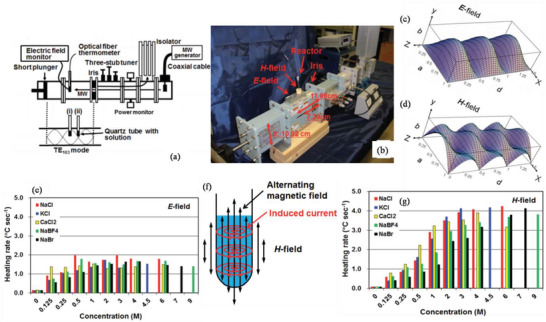
a) Schematics of the experimental setup; locations of the samples in the single‐mode MW (TE103) resonator; (i) location of the maximal *E*‐field density; (ii) location of the maximal *H*‐field density; b) photograph of the single‐mode MW resonator and the 2.45 GHz semiconductor MW generator; c) profile of the *E*‐field and d) *H*‐field component of MW radiation inside a waveguide in single‐mode operation, resonant condition. Note: *E*‐field occurs only in the direction of the *Y*‐axis. *E_y_*: *E*‐field vector in the *Y* direction; *H_x_* and *H_z_*: *H*‐field vector of the *x* and *z* direction; a: long side of the waveguide; b: short side of the waveguide; d: MW propagation direction in the waveguide; e,g): heating rates of an ultrapure water sample and aqueous electrolyte solutions of NaCl, KCl, CaCl_2_, NaBF_4_, and NaBr obtained for a 30 s irradiation period in a quartz tube under; e) predominantly *E*‐field and; g) predominantly *H*‐field irradiation conditions; f) heating model for electrolyte/water solutions under predominant *H*‐field irradiation. Adapted with permission.^[^ ^175^ ^]^ Copyright 2012, Taylor & Francis.

The following study helps in understanding the effect of concentration of electrolytic solution, *E* and *H*‐fields, and container influence on the MW heating mechanism. Because the *E*‐field and *H*‐field of MWs interact with matter differently, the *E* and *H*‐field penetration depth in solution may differ significantly, resulting in distinct heating processes. The MW heating characteristics of aqueous electrolyte solutions such as NaCl, CaCl_2_, KCl, NaBr, and NaBF_4_ (under 2.45 GHz MW, input power of 40 W) are reported.^[^ ^175^ ^]^ The quartz tube with electrolyte solutions was placed either at the position of maximal *E*‐field density or maximal *H*‐field density inside the waveguide (**Figure** **39**a). An optical fiber thermometer was used to measure the temperature of the solutions at 5‐second intervals. Under the *E*‐field, heating rates have plateaued at low electrolyte concentrations and remained constant at higher concentrations, as shown in Figure 39e. On the other hand, under *H*‐field irradiation, the heating rates of aqueous electrolyte solutions exhibited near‐exponential growth, demonstrating that *H*‐field heating efficiency is higher than *E*‐field heating (Figure 39g). This contrast was explained on the basis of penetration depth. When an electrolyte is mixed with water, MW heating is dominated by Joule heating under both *E*‐field and *H*‐field irradiation. Under *E*‐field irradiation, the penetration depth becomes shallow with an increase in electrolyte concentration. However, in the *H*‐field, the heating is independent of penetration depth as the solution does not absorb the *H*‐field component. Instead, as shown in Figure 39f, the oscillating *H*‐field component can penetrate the whole solution to induce ring currents, causing the solution to heat up. The MW heating effect of the addition of an electrolyte to polar solvents (e.g., H_2_O and EG) has been shown to be different. Therefore, the effect of MWs on water‐containing ions like salts is very significant relative to the effect of MWs on pure water alone. Near 0.5 m in NaCl, the heating rate at the middle of the electrolyte solution was rapid. However, for a higher concentration (2.0 m in NaCl), the heating rate decreased sharply. At the same time, when the electrolyte concentration increased, the heating rate at the container wall decreased smoothly. For a 2.0 m in NaCl solution, the heating rate at the wall surface was higher than at the center, indicating MWs are less likely to reach the center of the container. Such studies can help us select the best MW heating mode, aqueous electrolyte solutions, and containers for the synthesis of TE nanophase materials.

#### 2.5.3 Flow‐Type Tubular MW Reactor

**Figure 40:**
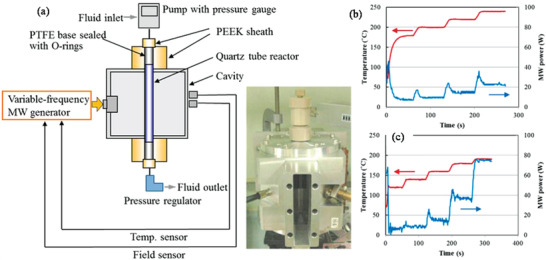
a) Experimental setup of the MW‐assisted flow reactor system; a photograph of the MW cavity. Temperature and applied MW power time profile for the stepwise heating of EG and EtOH at the flow rate of 10 mL h^−1^; b) EG (180–240 °C, 2 MPa); c) EtOH (120–190 °C, 6 MPa). Adapted with permission.^[^ ^409^ ^]^ Copyright 2013, ACS.

The MW penetration depth in most solvents is only a few cm. As a result, heat distribution is often inhomogeneous. Furthermore, in chemical reactions, increasing the temperature increases the rate of chemical reactions. However, the BP of solvents regulates the upper limit of the reaction temperature. Therefore, if the BP of the solvent is elevated beyond its natural limit via applying pressure, reactions can be performed at higher temperatures.^[^ ^159b^ ^]^ To address such problems, a flow‐type tubular MW reactor equipped with an elevated‐pressure attachment has been demonstrated (**Figure** **40**).^[^ ^409^ ^]^ This MW reactor may produce a uniform electromagnetic field along a quartz glass tube reactor (1.5 mm 100 mm) located at the center of a cylindrical MW cavity. The main feature of this flow‐type MW reactor system is the automatic detection and adjustment of the resonance frequency shift. The system consists of a variable frequency MW generator (2.5 GHz ± 200 MHz, 100 W), a pressure pump, and a TM010 single‐mode cavity. The pump pressurized the reaction fluid and supplied it to the quartz reactor tube inlet. The pressure can be controlled manually using a pressure regulator and a pressure gauge can be used to measure it. The quartz reactor tube is connected to the PEEK sheath that is designed to tolerate up to 10 MPa. Polar liquids with significantly higher dielectric loss characteristics were used to test the MW heating. The water temperature was raised step wise to ≈200 °C (without boiling) under a pressure of 2 MPa and a flow rate of 10 mL h^−1^. Similarly, EG (BP ≈ 195 °C) and EtOH (BP ≈ 78 °C) were heated gradually to 240 °C and 190 °C, respectively, by the application of 2 and 6 MPa and a flow rate of 10 mL h^−1^. Such systems are ideal for accelerating and scaling up nanomaterial production.

In the MW cavity, the MW field distribution in the absence of a sample may be different when compared to the distribution in the presence of a sample (i.e., the field gets distorted).^[^ ^410^ ^]^ Additionally, the exact temperature of the core of the condensed sample that is exposed to MWs is difficult to ascertain. Although the 2.45 GHz frequency is commonly used in thermoelectrics, it is interesting to see how some lower frequencies (e.g., 915 MHz) can play a role in TE material synthesis. The relationship between sample size and required MW frequency must be established through numerical modeling in order to grasp ideal heating rates and energy consumption efficiency especially in MW solid‐state synthesis.

Numerical modeling has been applied to ascertain the influences of sample dimension (cylinders of varying radii), susceptor, and physical properties on the heating behavior of samples under MWs. It was revealed that the effectiveness of MW heating depends on the sample size and its *κ*.^[^ ^411^ ^]^

It is clear from Equation 9 that MW absorption in a material is dependent on the incident field strength (square of *E* and *H*‐field strengths: $H_{\mathrm{rms}}^{2}$and $E_{\mathrm{rms}}^{2}$). The field strength can be enhanced by the interference of the waves inside the waveguide. For example, the introduction of foreign support: metallic support or metallic–ceramic support can be an effective way to strengthen constructive interference as well as samples of different shapes.^[^ ^176^, ^412^ ^]^

Characterization of temperature‐dependent dielectric and magnetic parameters is very important when the materials go into large‐scale production for a specific application. These data give an estimation of penetration depth and powder size. To overcome the hot spots problems, MWs can be used to carry out conventional heating without dielectric loss as done in the case of SiC vessel^[^ ^413^ ^]^ where the sample containing vessel itself gets heated under MW irradiation uniformly (via resistance heating), and then heats the sample solution via conduction and convention.

## 3 Conclusions

In this article, we provided a comprehensive review of the synthesis aspects of thermoelectric nanomaterials focusing on HT/ST, MW‐assisted, solution‐based and powder processing methods along with the summary of recent work carried out in the field and presented the TE properties of the reported materials. In terms of yield, crystallinity, energy efficiency, scalability, processability, and TE performance, ST/HT, solution‐based, and ball milling (mechanical alloying) methods are very appropriate for nanostructured material fabrication. As fabricated TE nanomaterials in conjunction with suitable conducting polymers can yield efficient functional inks/pastes for various types of printers: inkjet, dispenser, screen printers for the mass production of wearable devices. As mentioned early, as compared to solution‐based synthesis, MW‐assisted synthesis still has many deficiencies in term of level of flexibility and control over composition, size, shape, and crystal structure. Using MW technique, fabrication of multi‐component (composites) TE materials is sparsely tackled. It is very critical to investigate the interaction of MWs with the reagents and the evolution of the nanophase materials under MW electromagnetic field. High equipment cost is one of the major hurdles in the MW chemistry research. However, with the growing interest in MW synthesis of nanomaterials for various technological applications, the situation may change in the near future. Successfully obtaining a desired phase in a small MW reactor setting may be auspicious. However, to replicate the same technique for large‐scale synthesis, a minute detail of the influence of the MW field on every reaction step of the reaction is needed to design suitable equipment. For MW‐assisted methods, a well‐formulated etiquette and strict adherence to them is needed otherwise poor connection from theoretical chemical synthesis to technical engineering sciences may incur.

Solution‐based synthesis gives freedom to control particle size and their morphology and therefore such nanoparticles can be used to tune the microstructures of TE materials for better performance. The ball‐milling method continues to be a valuable and versatile tool in the fabrication of nanostructured TE materials and alloys. Many states of the art TE materials developed so far harmful to the environment. These materials are going to be in the realm of scientific research. Wherever possible, whether in industry or in the research lab, paying attention to 12 green chemistry principles can assist to reduce the environmental threat. Exploration of green pathways to replace the contemporary harmful reagents with environmentally benign materials is crucial.

## Conflict of Interest

The authors declare no conflict of interest.

## Supporting information

Supporting Information
