# Supplementary material for: Energy‐Saving Pathways for Thermoelectric Nanomaterial Synthesis: Hydrothermal/Solvothermal, Microwave‐Assisted, Solution‐Based, and Powder Processing
Source: Adv Sci (Weinh). 2022 Jul 17;9(25):2106052. doi: 10.1002/advs.202106052 (PMC9443476; doi:10.1002/advs.202106052)
Supplement: Supplementary file 1 — Supporting Information [file ADVS-9-2106052-s001.pdf]

ADVANCED SCIENCE
Open Access

Supporting Information

for *Adv. Sci.*, DOI 10.1002/advs.202106052

Energy-Saving Pathways for Thermoelectric Nanomaterial Synthesis: Hydrothermal/Solvothermal, Microwave-Assisted, Solution-Based, and Powder Processing

Nagaraj Nandihalli, Duncan H. Gregory and Takao Mori**

SUPPLEMENTARY INFORMATION

Energy-savingpathways for thermoelectric nanomaterial synthesis: Hydrothermal/solvothermal, microwave-assisted, solution-based, and powder processing

Nagaraj Nandihalli,[a,✉] Duncan H. Gregory[b],and Takao Mori[a,✉]

[a] National Institute for Materials Science (NIMS), International Center for Materials Nanoarchitectonics (WPI-MANA), Namiki 1-1, Tsukuba 305-0044, Japan

[b] WestCHEM, School of Chemistry, University of Glasgow, UK

Takao Mori ✉MORI.Takao@nims.go.jp |Nagaraj Nandihalli ✉nandihalli.nagaraj@gmail.com

List of Tables

Table 1: Physical parameters of common solvents used in ST synthesis.[1]

Abbreviations

M_r—Molecular weight, d—density (g cm^{-3}), M_p—melting point (°C), B_p—boiling point (°C), ε—dielectric constant (C^2 N^{-1} M^{-2}), μ_d—dipole moment, E^T_N—solvent polarity

Solvent	M_r	d	M_p	B_p	ε	μ_d	E^T_N
Water	18.01	1.000	0	100	80.4	1.94	1.000
Methyl alcohol	32.04	0.791	-98	65	32.6	1.70	0.762
Ethyl alcohol	46.07	0.785	-130	78	24.3	1.69	0.654
Propanol	60.10	0.804	-127	97	20.1	1.66	0.602
2-Propanol	60.10	0.785	-90	82	18.3	1.66	0.546
Butanol	74.12	0.810	-90	118	17.1	1.66	0.602
2-Butanol	74.12	0.807	-115	98	15.8		0.506
2-Methyl-1-propanol	74.12	0.802	-10	108	17.7	1.64	0.552

2-Methyl-2-propanol	74.12	0.786	25	83			0.389
Pentanol	88.15	0.811	-78	137	13.9	1.80	0.568
3-Methyl-1-butanol	88.15	0.809	-11	130	14.7	1.82	0.565
2-Methyl-2-butanol	88.15	0.805	-12	102	7.0	1.70	0.321
Hexyl alcohol	102.18	0.814	-52	157	13.3		0.559
1-Heptanol	116.20	0.822	-36	176	12.1		0.549
Cyclohexanol	100.16	0.963	21	160	15.0	1.90	0.500
Benzyl alcohol	108.14	1.045	-15	205	13.1	1.70	0.608
Ethylene glycol	62.07	1.109	-11	199	37.7	2.28	0.790
1,3-Propanediol	76.10	1.053	-27	214	35.0	2.50	0.747
1,2-Propanediol	76.10	1.036	-60	187	32.0	2.25	0.722
1,4-Butanediol	90.12	1.017	16	230	31.1	2.40	0.704
1,3-Butanediol	90.12	1.004	-50	207			0.682
Diethylene glycol	106.12	1.118	-10	245			0.713
Triethylene glycol	150.18	1.123	-7	287	23.7	5.58	0.704
Tetraethylene glycol	194.23	1.125	-6	314			0.664
Glycerol	92.09	1.261	20	180	42.5		0.812

Table 2: Loss tangent (tanδ) for different solvents.[2] Data acquired at Frequency of MW radiation: 2.45 GHz and 20 ^oC. [bmim]PF_6 = 1-butyl-3- methylimidazolium hexafluorophosphate. NMP=N-methyl-2-pyrrolidone; BP = boiling point

Solvent	BP (^oC)	tanδ	Solvent	BP (^oC)	tanδ
Ethylene glycol	198	1.350	DMF	153	0.161
Ethanol	78	0.941	1,2-dichloroethane	84	0.127
DMSO		0.825	Water	100	0.123
2-propanol	82	0.799	Chlorobenzene	131	0.101
formic acid	100.7	0.722	Acetonitrile		0.062

Methanol	65	0.659	Acetone	56-57	0.054
1,2-dichlorobenzene	180.5	0.280	Tetrahydrofuran	66	0.047
NMP	202	0.275	Dichloromethane	39.8	0.042
[bmim]PF_6	-	0.185	Toluene	111	0.040
acetic acid	118-119	0.174	Hexane	68-69	0.020
Glycerol		0.651	1,3-propanediol		1.30 (at 25 ^{o}C)
1,4-butanediol		0.783	1,5-pentanediol		0.456
Nitrobenzene		0.589	Benzaldehyde		0.337

Table 3: Relaxation times (at 20 °C) and dielectric properties of aliphatic alcohols in comparison to H_2O.[3] τ: relaxation time (10^{-12} sec); μ_d: Dipole moment (Debye); η: Viscosity (milli poise)

Compound	τ	μ_d	η	tanδ at 2.45 GHz
H_2O	9.04	1.84	10.1	0.123
MeOH	51.5	1.7	5.45	0.659
EtOH	170	1.69	10.8	0.941
Propan-1-ol	332	1.68	20	0.757
Propan-2-ol	237	1.66	17.7	0.799
Butan-1-ol	538	1.66	22.7	0.571
Butan-2-ol	562			0.447
2-Methylpropan-1-ol	644			0.522
Pentan-1-ol	792	1.8	33.5	0.427
Hexan-1-ol	976	1.67		0.344
Pent-4-en-1-ol	322			0.669
Pent-3-en-2-ol	169			0.72
3-Methyl but-2-en-1-ol	170			0.846
trans-Hex-2-en-1-ol	277			0.571
Benzyl alcohol	188			0.667

Table 4: MW-receptive elements, minerals, and compounds.[4]

Element/mineral/compound	time (min) of MW exposure	*T*(K)	Element/mineral/compound	time (min) of MW exposure	*T*(K)
Al	6	850	NiO	6.25	1578
C (amorphous, < 1 mm)	1	1556	V_2O_5	11	987
C (graphite, 200 mesh)	6	1053	WO_3	6	1543
C (graphite, < 1 μm)	1.75	1346	Ag_2S	5.25	925
Co	3	970	Cu_2S (chalcocite)	7	1019
Fe	7	1041	$CuFeS_2$ (chalcopyrite)	1	1193
Mo	4	933	$Fe_{1-x}S$ (phyrrhotite)	1.75	1159
V	1	830	FeS_2 (pyrite)	6.75	1292
W	6.25	963	MoS_2	7	1379
Zn	3	854	PbS	1.25	1297
TiB_2	7	1116	PbS (galena)	7	956
Co_2O_3	3	1563	CuBr	11	995
CuO	6.25	1285	CuCl	13	892
Fe_3O_4 (magnetite)	2.75	1531	$ZnBr_2$	7	847
MnO_2	6	1560	$ZnCl_2$	7	882

Table 5: Material types, synthesis/consolidation methods, morphologies/dimensions, and *zT* of recently reported TE nanomaterials and composites.

Abbreviations

ST—solvothermal method, HT—hydrothermal method, SB—solution-based method, MW—microwave, HP—hot press, SPS—spark-plasma-sintering, NWs—nanowires, NSs—nanosheets, NRs—nanorods, NPs—nanoparticles, NTs—nanotubes, NPts—nanoplates; NFs—nanoflakes; RT-room temperature

Material	Synthesis/sintering method	Morphology/size	*zT*/Temperature (K); lowest κ (W/m-K)	Ref
n-type Se-doped Bi_2Te_3	MW-assisted ST/SPS (250 ˚C, 40 MPa, 5 min)	NPts<br>Lateral size ~1 μm	For $Bi_2Te_{2.7}Se_{0.3}$, 1.23/480 K; 0.69	[5]

n-type Ce-doped Bi_2Te_3 NSs	HT/HP (773K, 60 MPa15 min)	Thickness of sheet crystals <100 nm	For $Ce_{0.2}Bi_{1.8}Te_3$ 1.29/398 K; 0.5	[6]
n-type Y-doped Bi_2Te_3	HT/HP (773 K, 60 MPa)	Sheet-like crystals; thickness < 100 nm	For $Y_{0.25}Bi_{1.75}Te_3$ 1.23/410 K; 0.82	[7]
p-type $Bi_{0.4}Sb_{1.6}Te_3$	HT/HP (673 K, 50 MPa, 20 min)	Platelets: 55 nm	1.26/398 K; ~1	[8]
n-type Bi_2Te_3	SB/SPS (678 K, 50 MPa, 5 min)	NWs: avg. diameter, 8 nm	0.96/380 K; 0.92	[9]
p-type $Bi_{0.5}Sb_{1.5}Te_3$	HT/SPS (400 ^oC, 30 MPa, 3 min)	Irregular hexagonal sheets (thickness = ~ 30-100 nm and the sidelength from hundreds of nm to several μm)	0.86/373 K; 0.82	[10]
n-type Bi_2Te_3/Bi_2Te_3 (NTs)	HT/HP (350 °C, 50 MPa, 30 min)	NTs: diameters ≈30-100 nm; few μm in length & spiral tube-walls; 20 nm thick walls	~1.0/450 K; κ_l = 0.3	[11]
p-type $(Bi,Sb)_2Te_3$	SB/HP	Nanocrystals < 20 nm	0.91/380 K	[12]
n-type $Bi_2Te_{3-x}Se_x$ ($0.6 \leq x \leq 0.75$)	HT/SPS (450 ^oC, 470 ^oC, 45 MPa, 5 min)	NanoplateletSheet size: 50 nm	For $Bi_2Te_{2.25}Se_{0.75}$ 0.96/490 K; κ_l~ 0.26	[13]
$(Sb, Bi)_2(Te, Se)_3$ (*n*-type and *p*-type)	Aqueous solution method/HP (400 ^oC, 50 MPa, 30 min)	NPs< 10 nm in size	0.86/498 K; 0.55-0.75	[14]
n-type $Bi_2Te_{2.85}Se_{0.15}$	Water-based chemical reaction/SPS (350 ^oC, 50 MPa, 2 min)	NPs < 100 nm	0.7/423 K; κ_l~ 0.57	[15]
n-type Bi_2Te_3 monolith with porosity	SB/HP (380 °C, 70 MPa)	NPs: 10 - 80 nm (SPSed)	0.7/480 K; < 1.2	[16]

n-type BiTe	Water-based chemical reaction/SPS (400 °C, 50 MPa, 10 min)	Irregular shaped NPs	0.91/373 K; ~1	[17]
Cu-doped Bi_2Te_3	SB/SPS (673 K, 50 MPa,5 min)	Flower-like agglomerates (~ 200 nm)	For Cu-doped Bi_2Te_3 (15.6 at% Cu), zT ~0.67/415 K	[18]
Bi_2Te_3 NWs/graphene Oxide (GO)	SB/HP (623 K, 50 MPa, 10 min)	Bi_2Te_3NW:<br>Length: Bi_2Te_3 ~μm;~15 nm in diameter; GO: several μms across and 4–5 nm thick	*PF* of Bi2Te3 NW/0.5 wt% graphene ~<br>688.9 μW/m-K^2 at RT	[19]
Bi_2Te_3	SB/SPS (350 ˚C,50 MPa, 2 min)	grain size: 300–500 nm	Parallel to pressing direction and 6T magnetic field: zT~0.5; κ~0.94	[20]
Bi_2Te_3	HT/SPS (673 K, 60 MPa,5 min)	NTs:<br>length: several μm; diameter ~100 nm	~ 0.77/464 K;0.62 W/m-K at 470 K	[21]
n-type $Bi_2Te_{2.7}Se_{0.3}$	Wetchemical synthesis/SPS (<br>350°C, 50 MPa, 2 min)	< 10 nm in size	0.82/298 K and<br>0.92/373 K	[22]
n-type La-doped Bi_2Te_3	HT/HP (523 K, 75MPa, 15 min)	Nanoflowers thickness: tens of nm	For $La_{0.2}Bi_{1.8}Te_3$<br>0.58/480 K; 0.9	[23]
p-type $Sn_xSb_2Te_{3+x}$ (x = 0, 0.02 and 0.05)	ST/SPS (300 °C, 50 MPa, 3 min)	Wire-like Sb_2Te_3NPs	For $Sn_{0.02}Sb_2Te_{3.02}$<br>0.42−0.58/323−423K; 0.7−0.5	[24]
n-type Bi_2Te_3/graphene quantum dots (GQD)	SB/SPS (350 °C, 50 MPa, 5 min)	GQDs: 20 nm<br>Bi_2Te_3/GQDs NSs: thickness of ~ 10 nm	0.55/425 K; 0.65	[25]
n-type Bi_2Te_3	ST/Cold pressing (20 MPa)	Hierarchical flower-like Bi_2Te_3composed of	0.6/600 K; 0.56	[26]

		smaller NPts (avg. thickness: 30 nm)		
n-type Bi_2Te_3/Graphene composite	HT/SPS (350 °C, 80 MPa, 6 min)	Bi_2Te_3 nanopowder Size: 30-200 nm	For 0.2 vol% graphene sample, 0.21/475 K; ~2.2	[27]
n-Type Bi_2Te_3 NW/graphene	Wet chemical synthesis/HP (673 K, 50 MPa, 10 min)	NWs length ~ 1 mm; diameter 15 nm	0.2/RT (20 wt%. NW content); 0.7	[28]
n-type Bi_2Te_3	HT/SPS or high pressure and high temperature (HPHT) sintering	NRs size ~ 500 nm in length; 20 nm in diameter	0.42 (SPSed) and 0.47 (HPHT) at 430 K; 0.58 & 0.28	[29]
p-type Sb_2Te_3 nanosheets	MW-assisted ST/ SPS (543 K, 67 MPa, 6 min)	NSs (thickness ~50-70 nm; edge length 300-500 nm)	0.58 at 420 K; κ (1.0–0.76 W/m-K)	[30]
p-type $Bi_{0.5}Sb_{1.5}Te_3$	SB/cold-press and sintering	Nanocrystals: 30 nm.	0.42/275 K for sintered pellet at 598 K; 0.79	[31]
p-type Sb_2Te_3	SB (low energy reflux method)/ Cold pressing and annealing	shape, size, and composition depended on organic surfactants	~0.24−0.37/323–573 K; 1.32-1.55	[32]
Sb_2Te_3	MW-heating, SB/cold pressing (815 MPa, 30 min)/annealing at 300 °C	Spherical agglomerates > 2 μm	~1.5 at 300 °C and κ of 0.29 to 0.27	[33]
p-type Cu_2SnSe_3	SB/SPS (92 MPa, 5 min)	Nanocrystals, 12- 15 nm	0.3/730 K; ~0.6	[34]
p-type$Cu_{2-x}Se$	Aqueous route/SPS (703 K, 65 MPa,10 min)	NW bundles diameter: 100-300 nm; length: tens of μm	0.29 at 750 K; κ = 1.75at 750 K	[35]
p-type I-doped Cu_2Se	HT/HP (773 K, 35 MPa, 40 min)	hexagonal NSs	For $Cu_2Se_{1-x}I_x$ (x = 0.03): 1.1/773; 0.8	[36]

p-type $Cu_{1.9}8Li_{0.02}Se$	ST/HP (1098 K, 60 MPa, 15 min)	hexagonal NPts; self-assembled plate-like (lateral size of 100–500 nm)	2.14/973 K; κ_l=0.3 at 973 K	[37]
$Cu_{2-x}Se$	ST (90 °C for 4 h)	Spherical (18 nm)	-	[38]
Cu_2Se + 0.8 wt% carbon nanodots(CDs)	HT/HP (1098 K, 60 MPa, 15 min)	typical hexagonal NPts with transverse sizes of 50-100 nm	1.98/973 K; κ~0.45 W/K-m/973K	[39]
Te-doped Cu_2Se	ST (230 °C for 24 h)/SPS (800K,50MPa, 5min)	NPts ~50 nm	For $Cu_2Se_{0.98}Te_{0.02}$, ~1.2/400 K - 850 K; κ ~ 0.3~0.6	[40]
p-type SnSe	SB/SPS (different temperatures)	polycrystalline (several hundred nm after SPS)	~0.47/430 °C for sample sintered at 550 °C, ; κ_l= 0.8	[41]
p-type $Sn_{0.95}Se$	ST (403 K for 36 h)/SPS (693 K,50 MPa, 7 min)	nanodomains (~5 nm)	~2.1 at 873 K	[42]
p-type SnSe	HT (150–170 °C)/evacuating-and-encapsulating sintering (450–580 °C)	polycrystalline (several tens of nm)	0.54/550 K; 0.35 to 0.5	[43]
p-type SnSe	HT/SPS (693 K, 50 MPa, 7 min)	oval and elongated precipitates (10–30 nm)	1.3/850 K; 0.26	[44]
p-type SnSe	ST/SPS (500 °C, 50 MPa, 5 min)	flower like, NR, plate-shaped particles (length ~200 nm & width ~ 2 to 5 μm)	0.6/773 K; < 1	[45]
p-type SnS	ST/cold isostatic pressing, rapid annealing	hexagonal NRs: size = ~150 nm × 1000 nm; thickness ~30 nm	0.25/873 K; ~0.35	[46]
$Sn_{1-x}Se$	ST (230 °C for 48	polycrystalline	1.5/823 K ;0.41	[47]

	h)/SPS (573 C, 70 MPa,5 min)			
p-type SnSe NSs /PEDOT:PSS composite	Films	sheet-like structures 200−500 nm	For 20 wt%SnSe NSs 0.32/300 K; < 0.40	[48]
n-type Ag_2Te (S-doping)	ST/HP (673 K, 50 MPa, 30 min)	NPs and NPts (5-10 nm)	0.62/550 K; 0.36; κ_l = 0.2± 0.1	[49]
$AgPb_{10}BiTe_{12}$	ST/cold pressing	$AgPb_{10}BiTe_{12}$ NSs: diameters = 20 - 100 nm	p to n transition at high temperature 0.46/570 K; 0.56	[50]
n-type $CoSb_3$	MW-assisted/cold isostatic pressing (CIP) and sintering	NPs ~10 nm	0.11/650 K; 5.71	[51]
Te-doped, and Sn-doped $CoSb_3$	MW-assisted modified polyol process	Spherical-like particles ~10 nm	-	[52]
p-type $Cu_{12}Sb_4S_{13}$	ST/HP (430 °C, 80 MPa, 30 min)	−	0.85/720 K; κ<0.4 at 300 K	[53]

MW Solid-state synthesis<br>*Abbreviations:* MA-Mechanical alloying				
Material	Starting materials/sintering	MW type, power, duration etc.	Outcome	Ref.
Mg_2Si doped with Ag, Sn, Sb, Co, and Bi	Dry ball-milled/SPS (775 °C, 90 MPa)	TE102 mode	For $Mg_{1.99}SiAg_{0.01}$ (p-type) & $Mg_2Si_{0.5875}Sn_{0.4}Sb_{0.0125}$ (n-type), zT ~ 0.35 & 0.7 at 775 K resp.	[54]
p-type $Bi_{0.4}Sb_{1.6}Te_3$	MA/MW activated HP (MAHP); 50 MPa; SiC cylindersurrounded graphite die	2.45 GHz	κ_l~0.41 W/m-K and zT of 1.13 (at 373 K) for sample sintered at 648 K and 15 min	[55]
n–type $Bi_2Te_{3-x}Se_x$	MA/MW activated HP (MAHP); 50 MPa; 613 K for 10 min; SiC mold	2.45 GHz	For $Bi_2Te_{2.55}Se_{0.45}$ zT of 0.73 at 423 K;	[56]

(x=0.15, 0.3, 0.45, 0.6)	surroundedgraphite die		Fine lamellar grains and irregular granular grains	
p-type $Bi_{0.49}Te_3Sb_{1.51}$	MA/MW-sintering; SiC barrel around the sample	Multimode cavity; 2.45 GHz; 600 W(400 to 420 °C); 10 min soaking	σ ~49,600 S/m; S ~230 μV/K; n ~2.3 × 10^{19} cm^{-3}; 133 cm^2/V-s; PF ~ 2.62 × 10^{-3}; 1.06 W/m-K; zT ~0.74	[57]
Se and Te doped skutterudite $Co_4Sb_{11.9-x}Te_xSe_{0.1}$ (x = 0.2, 0.3, 0.4, 0.5, 0.6)	Mixed Co (~48 μm), Sb (~20 μm), Te (~75 μm), & Se (~75 μm) powders in palette form/SPS (5 min, 903 K)	700 W; 5 min	For $Co_4Sb_{11.5}Te_{0.4}Se_{0.1}$ zT ~0.81 at 773 K and κ of 2.8 W/m-K	[58]
$Pb_{1-x}Yb_xTe$	Pb, Te, and Yb powders	2.54 GHz, 800W; 25 min	For x = 0.015, PF ~5.015 W/m-K^2 at RT; S ~ 265 μV/K; 71.4 S/cm	[59]
MgAgSb/Ag_3Sb composite	Mg strip, Ag and Sb powders/SPS (723 K, 10 min, 60 MPa)	1273 K for half one hour; SiC as susceptor	PF ~2000 μW/m-K^{-2}, κ_l ~0.70 W/m-K; zT ~0.76 at 548 K.	[60]
Hexagonal rods of $Bi_{0.4}Sb_{1.6}Se_{3x}Te_{3(1-x)}$ (0.0≤x≤1.0)	Mixed (mortar & pestle) Bi, Sb, Se, and Te powders/Cold pressing (10 ton)	800 W MW oven at 2.45 GHz/10 min (873 - 903 K)	$PF$of 7.47 mW/m-K^2 at 373 K (for x = 0.8); S~ 159 μV/K	[61]
α-$MoSi_2$	MA (4 h) /pressed palette (900 MPa); MW activated sintering (973 K - 1273 K for 1 h)	1400 W/90 min (1273 K); SiC powders bed as susceptor	PF of 6.2 μ W/m−K^2 at 673 K	[62]
TiNiSn and TiCoSb Half-Heuslers	Cold-pressed metal powders/Hot-pressed (1173 K, 84 MPa, 60 min)	Household MW; 700 W/1 min; granular C susceptor	For TiNiSn, zT ~0.45 at 740 K, κ~4.6; for TiCoSb,zT ~0.004 at 700 K, κ~12	[63]
ZnTe nanocrystals	Zn and Te powders	900 W;MW plasma/different time intervals	73.37 nm nanocrystals	[64]

n-type In filled skutterudite ($In_{0.2}Co_4Sb_{12}$)	compacted starting materials	2.45 GHz, 2 min at 750 W; CuO as susceptor	*zT* ~ 0.85 at 625 K	[65]
$Ca_3Co_4O_9$	BM, palettezed& MW reactive sintering	multimode 2.45 GH MWs	*ρ* (150-160 μΩ-m) &*PF* (94–145 μW/m-K^2) in 35–500 °C	[66]

Table 6: Reported work based on BM for different material types

Alloy type	Material	Method, *zT/T*(K)	Reference
Bi_2Te_3 based alloys	BiSbTe/SiC	MA + SPS; 1.33/373 K	[67]
	BiSbTe	Melt-BM + HP; 1.3/380 K	[68]
	n-type Bi_2Te_3	Melt-BM + HP; 0.72/373 K	[69]
	$ZnO/Bi_2Te_{2.7}Se_{0.3}$	BM + SPS; 1.3	[70]
	BiTeSe	MA + SPS; 0.82/473 K	[71]
	$Cu_{0.01}Bi_2 Te_{2.7}Se_{0.3}$	BM + dc-HP; 1.06/373 K	[72]
	$Bi_{0.5}Sb_{1.5}Te_3$	M + MA+ SPS; ~1.86/320 K	[73]
	p- type $Bi_{0.3}Sb_{1.7}Te_3$	BM + HP; 1.12/350- 375 K	[74]
	p-type $Bi_{0.5}Sb_{1.5}Te_3$ and *n*-type $Bi_2Te_{2.7}Se_{0.3}$/SiC nano (5 vol%)	MA+SPS;0.97 at 323 K for *p*-type $Bi_{0.5}Sb_{1.5}Te_3$(0.1 vol% SiC)	[75]
PbTe based alloys	$Pb_{1-x}Sn_xSe$	MA + SPS; 1.0/773 K	[76]
	Tl-doped PbTe	BM + HP; 1.3/673 K	[77]
	Tl-doped-PbSe	BM + HP; 1.3/850 K	[78]
	$Ag_{0.8}Pb_{18+x}SbTe_{20}$	MA + SPS; 1.37/673 K	[79]
	$AgPb_mSbTe_{m+2}$/1 vol% SiC nano	MA+SPS; 1.54/723 K	[80]

	$Pb_{0.95}Ce_{0.05}Te$	IM+BM+SPS; 0.88/673 K	[81]
	n-type PbTe dopedNa_2Te	BM+HP; 0.81/700 K	[82]
	$AgPb_mSbTe_{m+2}$	MA + SPS; 1.54/723 K	[83]
Selenides and sulfides	$CuFeS_2$	MA + SPS; 0.21/573 K	[84]
	SnS	MA + SPS; 0.16/823 K	[85]
	$CoSb_{3-x}Te_x$	MA + SPS; 1.1/823 K	[86]
	Cu_3SbSe_3	MA + SPS; 0.25/650 K	[87]
	Cu_3SbSe_4	MA + SPS; 0.7/673 K	[88]
Half-Heuslers	(ZrHf)Co(SbSn)	Ingot-BM + HP; 0.8/973 K	[89]
	FeVSb	MA + SPS; 0.31/573 K	[90]
	TiNiSn	MA + SPS; 0.32/785 K	[91]
	$ZrCoBi_{0.65}Sb_{0.15}Sn_{0.20}$	BM+HP; 1.42/973 K	[92]
	(HfZr)Ni(SnSb)	Melt-BM + HP; 1.0/873 K	[93]
	$(Hf_{0.25}Zr_{0.75})_{1-x}Nb_xNiSn$	For Nb doping (1.8 at% to 2.2 at%), ~0.9/700 °C	[94]
	$Ge_{0.99}Bi_{0.05}Te$	MA + SPS; ~2.0/650 K	[95]
	$Ge_{0.81}Mn_{0.15}Bi_{0.04}Te$	BM+SPS; ~1.1 (300-773 K)	[96]
	p-type $Si_{80}Ge_{20}$	BM+DC HP; $zT \sim 0.95$	[97]
	n-type SiGe	MA+SPS; 1.1/800 °C	[98]
	n-type SiGe	BM+HP; 1.84/1073 K	[99]
	p-type $Ge_{0.94}Bi_{0.06}Te$ + 0.2% nano-SiC	MA + SPS; ~2.1/723 K	[100]

Other materials	$Mg_2Sn_{0.75}Ge_{0.25}$	BM + HP; 1.4/723 K	[101]
	Cu_2Se/0.25 wt% graphene NPts	BM+SPS; 1.8/873 K	[102]
	p-type Cu_2Se + 0.15 wt% graphene	MA + annealing; 2.44/870 K	[103]
	p-type nano-Cu_2Se	MA + SPS; 2.1/973 K	[104]
	p-type Cu_2Se + 0.05 wt% SiC	MA + SPS; 2.0/850 K	[105]
	p-type Cu_2Se + 1 mol% In	MA + HP; 2.6/850 K	[106]
	Cu_2Se ($Cu_{1.98}Se$)	BM + SPS; 1.4/973 K	[107]
	Cu_2Se/0.15 wt% graphene	BM+melt-quench; ~2.44/873 K	[108]
	$K_{0.01}Sn_{0.99}Se$	MA + SPS; 1.1/773 K	[109]
	β- $Cu_{2-x}Ni_xSe$ (x = 0.0075 and 0.010)	Mealting+BM+quenching+SPS; 1.51/823 K	[110]
	$(Cu_2Se)_{0.9925}(CuGaSe_2)_{0.0075}$	melting +BM+SPS; 1.4 /823 K	[111]
	$Cu_{1.98}Se$	BM+SPS;~1.4 at 973 K,	[112]
	$Cu_2Se_{0.98}Te_{0.02}$	MA+SPS; 1.25/773 K	[113]
	$(Sn_{0.95}Pb_{0.05})_{0.99}Na_{0.01}Se$	MA + SPS; ~2.5/773 K	[114]
	SnSe	MA + 3D-film; 1.7/758 K	[115]
	SnSe	Fusion+BM+HP; 0.73/800 K	[116]
	SnSe	MA+SPS (100 MPa, zT ~0.71/873 K	[117]
	$Cu_{1.94}Al_{0.02}Se$	Melting+BM; 2.62/1029 K	[118]
	$Bi_{0.875}Ba_{0.125}Cu_{0.85}Ni_{0.15}SeO$	BM + SPS;0.97/923 K	[119]
	$Bi_{0.85}Na_{0.15}CuSeO$	BM+SPS; 0.7/873 K	[120]
	n-type Mg_2Ge	BM + SPS; 0.32/750 K	[121]

	Bi-doped $Mg_2Si_{0.6}Sn_{0.4}$	BM + sintering; 1.36/775 K	[122]

References

[1] R. Xu, Y. Xu, *Modern inorganic synthetic chemistry*, Elsevier, Amsterdam **2010**.

[2] J. Robinson, S. Kingman, D. Irvine, P. Licence, A. Smith, G. Dimitrakis, D. Obermayer, C. O. Kappe, *Phys. Chem. Chem. Phys.* **2010**, *12* (18), 4750, https://doi.org/10.1039/B922797K.

[3] C. Gabriel, S. Gabriel, E. H. Grant, B. S. J. Halstead, D. M. P. Mingos, *Chem. Soc. Rev.* **1998**, *27* (3), 213, https://doi.org/10.1039/A827213Z.

[4] a) S. L. McGill, J. W. Walkiewicz, G. A. Smyres, *MRS Online Proceedings Library Archive* **1988**, *124*, https://doi.org/10.1557/PROC-124-247; b) K. J. Rao, B. Vaidhyanathan, M. Ganguli, P. Ramakrishnan, *Chem. Mater.* **1999**, *11* (4), 882, https://doi.org/10.1021/cm9803859.

[5] M. Hong, T. C. Chasapis, Z. G. Chen, L. Yang, M. G. Kanatzidis, G. J. Snyder, J. Zou, *ACS nano* **2016**, *10* (4), 4719,

[6] F. Wu, H. Song, J. Jia, X. Hu, *Prog. Nat. Sci.: Mater. Int.* **2013**, *23* (4), 408,

[7] W. Shi, F. Wu, K. Wang, J. Yang, H. Song, X. Hu, *J. Electron. Mater.* **2014**, *43* (9), 3162,

[8] Z. Chen, G. D. Xu, S. Chen, J. Zhang, M. M. Wang, *J. Alloys Compd.* **2014**, *588*, 384,

[9] G. Zhang, B. Kirk, L. A. Jauregui, H. Yang, X. Xu, Y. P. Chen, Y. Wu, *Nano Lett.* **2012**, *12* (1), 56, https://doi.org/10.1021/nl202935k.

[10] Y. Zhang, G. Xu, J. Mi, F. Han, Z. Wang, C. Ge, *Mater. Res. Bull.* **2011**, *46* (5), 760, https://doi.org/10.1016/j.materresbull.2010.11.024.

[11] X. Zhao, X. Ji, Y. Zhang, T. Zhu, J. Tu, X. Zhang, *Appl. Phys. Lett.* **2005**, *86* (6), 062111,

[12] R. P. Gupta, J. Sharp, A. Peng, S. Perera, C. Ballinger, T. Zheng, B. Gnade, *J. Electron. Mater.* **2012**, *41* (6), 1573, https://doi.org/10.1007/s11664-011-1892-6.

[13] D. Li, X. Y. Qin, Y. F. Liu, N. N. Wang, C. J. Song, R. R. Sun, *RSC Adv.* **2013**, *3* (8), 2632, https://doi.org/10.1039/C2RA22562J.

[14] Z. Lu, L. P. Tan, X. Zhao, M. Layani, T. Sun, S. Fan, Q. Yan, S. Magdassi, H. H. Hng, *J. Mater. Chem. C* **2013**, *1* (39), 6271, https://doi.org/10.1039/C3TC31241K.

[15] C. Kim, D. H. Kim, Y. S. Han, J. S. Chung, S. Park, S. Park, H. Kim, *Mater. Res. Bull.* **2011**, *46* (3), 407,

[16] Y. Zhang, T. Day, M. L. Snedaker, H. Wang, S. Krämer, C. S. Birkel, X. Ji, D. Liu, G. J. Snyder, G. D. Stucky, *Adv. Mater.* **2012**, *24* (37), 5065, https://doi.org/10.1002/adma.201201974.

[17] C. Kim, D. H. Kim, H. Kim, J. S. Chung, *ACS Appl. Mater. Interfaces* **2012**, *4* (6), 2949, https://doi.org/10.1021/am3002764.

[18] J. An, M. K. Han, S. J. Kim, *J. Solid State Chem.* **2019**, *270*, 407,

[19] H. Ju, M. Kim, J. Kim, *J. Mater. Sci.: Mater. Electron.* **2016**, *27* (4), 3427,

[20] D. H. Kim, C. Kim, D. W. Ha, H. Kim, *J. Alloys Compd.* **2011**, *509* (17), 5211,

[21] H. T. Zhu, J. Luo, J. K. Liang, *J. Mater. Chem. A* **2014**, *2* (32), 12821,

[22] C. Kim, D. H. Kim, Y. K. Lee, J. T. Kim, Y. S. Han, H. Kim, *J. Alloys Compd.* **2014**, *584*, 108,

[23] Y. Zhang, T. Zhu, J. Tu, X. Zhao, *Mater. Chem. Phys.* **2007**, *103* (2-3), 484,

[24] H. Q. Yang, L. Miao, C. Y. Liu, X. Y. Wang, Y. Peng, A. J. Zhang, X. Y. Zhou, G. Y. Wang, C. Li, R. Huang, *Dalton Trans.* **2016**, *45* (17), 7483,

[25] S. Li, T. Fan, X. Liu, F. Liu, H. Meng, Y. Liu, F. Pan, *ACS Appl. Mater. Interfaces* **2017**, *9* (4), 3677,

[26] R. Jin, J. Liu, G. Li, *Cryst. Res. Technol.* **2014**, *49* (7), 460,

[27] B. Liang, Z. Song, M. Wang, L. Wang, W. Jiang, *J. Nanomater.* **2013**, *2013*, https://doi.org/10.1155/2013/210767.

[28] H. Ju, M. Kim, J. Kim, *Chem. Eng. J.* **2015**, *275*, 102,

[29] C. Yu, X. Zhang, M. Leng, A. Shaga, D. Liu, F. Chen, C. Wang, *J. Alloys Compd.* **2013**, *570*, 86, https://doi.org/10.1016/j.jallcom.2013.03.167.

[30] G. H. Dong, Y. J. Zhu, L. D. Chen, *J. Mater. Chem.* **2010**, *20* (10), 1976,

[31] J. S. Dyck, B. Mao, J. Wang, S. Dorroh, C. Burda, *J. Electron. Mater.* **2012**, *41* (6), 1408, https://doi.org/10.1007/s11664-012-1998-5.

[32] H. Q. Yang, L. Miao, C. Y. Liu, C. Li, S. Honda, Y. Iwamoto, R. Huang, S. Tanemura, *ACS Appl. Mater. Interfaces* **2015**, *7* (26), 14263, https://doi.org/10.1021/acsami.5b02504.

[33] S. Heimann, S. Schulz, J. Schaumann, A. Mudring, J. Stötzel, F. Maculewicz, G. Schierning, *J. Mater. Chem. C* **2015**, *3* (40), 10375, https://doi.org/10.1039/C5TC01248A.

[34] M. Ibáñez, D. Cadavid, U. Anselmi-Tamburini, R. Zamani, S. Gorsse, W. Li, A. M. López, J. R. Morante, J. Arbiol, A. Cabot, *J. Mater. Chem. A* **2013**, *1* (4), 1421, https://doi.org/10.1039/C2TA00419D.

[35] X. Chen, Z. Li, J. Yang, Q. Sun, S. Dou, *J. Colloid Interface Sci.* **2015**, *442*, 140,

[36] J. Wang, B. Liu, N. Miao, J. Zhou, Z. Sun, *J. Alloys Compd.* **2019**, *772*, 366,

[37] Q. Hu, Z. Zhu, Y. Zhang, X. J. Li, H. Song, Y. Zhang, *J. Mater. Chem. A* **2018**, *6* (46), 23417,

[38] W. Wang, P. Yan, F. Liu, Y. Xie, Y. Geng, Y. Qian, *J. Mater. Chem.* **1998**, *8* (11), 2321, https://doi.org/10.1039/A806166A.

[39] Q. Hu, Y. Zhang, Y. Zhang, X. J. Li, H. Song, *J. Alloys Compd.* **2020**, *813*, 152204,

[40] L. Yang, Z. G. Chen, G. Han, M. Hong, L. Huang, J. Zou, *J. Mater. Chem. A* **2016**, *4* (23), 9213,

[41] Q. K. Zhang, S. T. Ning, N. Qi, Z. Q. Chen, X. F. Tang, Z. Y. Chen, *J. Appl. Phys.* **2019**, *125* (22), 225109,

[42] W. Wei, C. Chang, T. Yang, J. Liu, H. Tang, J. Zhang, Y. Li, F. Xu, Z. Zhang, J. F. Li, *J. Mater. Chem. A* **2018**, *140* (1), 499,

[43] W. H. Chen, Z. R. Yang, F. H. Lin, C. J. Liu, *J. Mater. Sci.* **2017**, *52* (16), 9728,

[44] G. Tang, Q. Wen, T. Yang, Y. Cao, W. Wei, Z. Wang, Z. Zhang, Y. Li, *RSC Adv.* **2017**, *7* (14), 8258,

[45] Y. Li, F. Li, J. Dong, Z. Ge, F. Kang, J. He, H. Du, B. Li, J. F. Li, *J. Mater. Chem. C* **2016**, *4* (10), 2047,

[46] Q. Tan, C. F. Wu, W. Sun, J. F. Li, *RSC Adv.* **2016**, *6* (50), 43985,

[47] X. L. Shi, W. D. Liu, A. Y. Wu, V. T. Nguyen, H. Gao, Q. Sun, R. Moshwan, J. Zou, Z. G. Chen, *InfoMat* **2019**,

[48] H. Ju, J. Kim, *ACS nano* **2016**, *10* (6), 5730,
[49] W. Zhou, W. Zhao, Z. Lu, J. Zhu, S. Fan, J. Ma, H. H. Hng, Q. Yan, *Nanoscale* **2012**, *4* (13), 3926, https://doi.org/10.1039/C2NR30469D.
[50] Q. Wang, Y. Fang, H. Yin, J. Li, *Chem. Commun.* **2015**, *51* (9), 1594,
[51] Y. Zhu, H. Shen, H. Guan, *J. Mater. Sci.: Mater. Electron.* **2012**, *23* (12), 2210, https://doi.org/10.1007/s10854-012-0754-1.
[52] T. Kapanya, C. Thanachayanont, A. Tuantranont, T. Sarakonsri, in *Solid State Phenom.* Trans Tech Publ, **2020**, 123-134.
[53] D. J. James, X. Lu, D. T. Morelli, S. L. Brock, *ACS Appl. Mater. Interfaces* **2015**, *7* (42), 23623,
[54] D. Berthebaud, F. Gascoin, *J. Solid State Chem.* **2013**, *202*, 61, https://doi.org/10.1016/j.jssc.2013.03.014.
[55] X. Fan, F. Yang, Z. Rong, X. Cai, G. Li, *Ceram. Int.* **2015**, *41* (5), 6817,
[56] Z. Rong, F. Yang, X. Cai, X. Han, G. Li, *Mater. Res. Bull.* **2016**, *83*, 122,
[57] G. Delaizir, G. Bernard-Granger, J. Monnier, R. Grodzki, O. Kim-Hak, P. D. Szkutnik, M. Soulier, S. Saunier, D. Goeuriot, O. Rouleau, *Mater. Res. Bull.* **2012**, *47* (8), 1954, https://doi.org/10.1016/j.materresbull.2012.04.019.
[58] Y. Lei, W. Gao, R. Zheng, Y. Li, R. Wan, W. Chen, L. Ma, H. Zhou, P. K. Chu, *J. Alloys Compd.* **2019**, *806*, 537, https://doi.org/10.1016/j.jallcom.2019.07.231.
[59] A. Hmood, A. Kadhim, H. A. Hassan, *J. Alloys Compd.* **2012**, *520*, 1, https://doi.org/10.1016/j.jallcom.2011.12.044.
[60] S. Li, J. Yang, Y. Liu, J. Xin, S. Li, Q. Long, Q. Jiang, *Adv. Appl. Ceram.* **2020**, *119* (2), 107, https://doi.org/10.1080/17436753.2019.1705018.
[61] A. Kadhim, A. Hmood, H. A. Hassan, *Mater. Lett.* **2012**, *81*, 31,
[62] Y. Lan, M. Xie, T. Ouyang, S. Yue, *Mod. Phys. Lett. B* **2016**, *30* (19), 1650234,
[63] C. S. Birkel, W. G. Zeier, J. E. Douglas, B. R. Lettiere, C. E. Mills, G. Seward, A. Birkel, M. L. Snedaker, Y. Zhang, G. J. Snyder, *Chem. Mater.* **2012**, *24* (13), 2558, https://doi.org/10.1021/cm3011343.
[64] T. Suriwong, S. Thongtem, T. Thongtem, *Mater. Lett.* **2009**, *63* (24-25), 2103,
[65] K. Biswas, S. Muir, M. A. Subramanian, *Mater. Res. Bull.* **2011**, *46* (12), 2288, https://doi.org/10.1016/j.materresbull.2011.08.058.
[66] A. R. Annamalai, P. R. Teja, D. K. Agrawal, A. Muthuchamy, *Ceram. Int.* **2020**,
[67] J. Li, Q. Tan, J. F. Li, D. W. Liu, F. Li, Z. Y. Li, M. Zou, K. Wang, *Adv. Funct. Mater.* **2013**, *23* (35), 4317, https://doi.org/10.1002/adfm.201300146.
[68] L. Hu, T. Zhu, X. Liu, X. Zhao, *Adv. Funct. Mater.* **2014**, *24* (33), 5211, https://doi.org/10.1002/adfm.201400474.
[69] A. Kanatzia, C. H. Papageorgiou, C. H. Lioutas, T. H. Kyratsi, *J. Electron. Mater.* **2013**, *42* (7), 1652, https://doi.org/10.1007/s11664-012-2362-5.
[70] Q. Jiang, J. Yang, J. Xin, Z. Zhou, D. Zhang, H. Yan, *J. Alloys Compd.* **2017**, *694*, 864,

[71] Y. Pan, T. R. Wei, C. F. Wu, J. F. Li, *J. Mater. Chem. C* **2015**, *3* (40), 10583, https://doi.org/10.1039/C5TC02219C.
[72] W. S. Liu, Q. Zhang, Y. Lan, S. Chen, X. Yan, Q. Zhang, H. Wang, D. Wang, G. Chen, Z. Ren, *Adv. Energy Mater.* **2011**, *1* (4), 577,
[73] S. I. Kim, K. H. Lee, H. A. Mun, H. S. Kim, S. W. Hwang, J. W. Roh, D. J. Yang, W. H. Shin, X. S. Li, Y. H. Lee, *Science* **2015**, *348* (6230), 109,
[74] L. P. Bulat, V. T. Bublik, I. A. Drabkin, V. V. Karataev, V. B. Osvenskii, Y. N. Parkhomenko, G. I. Pivovarov, D. A. Pshenai-Severin, N. u. Tabachkova, *J. Electron. Mater.* **2010**, *39* (9), 1650,
[75] D. W. Liu, J. F. Li, C. Chen, B. P. Zhang, *J. Electron. Mater.* **2011**, *40* (5), 992,
[76] C. F. Wu, T. R. Wei, J. F. Li, *Phys. Chem. Chem. Phys.* **2015**, *17* (19), 13006,
[77] B. Yu, Q. Zhang, H. Wang, X. Wang, H. Wang, D. Wang, H. Wang, G. J. Snyder, G. Chen, Z. Ren, Thermoelectric property studies on thallium-doped lead telluride prepared by ball milling and hot pressing. American Institute of Physics: **2010**.
[78] Q. Zhang, H. Wang, W. Liu, H. Wang, B. Yu, Q. Zhang, Z. Tian, G. Ni, S. Lee, K. Esfarjani, *Energy Environ. Sci.* **2012**, *5* (1), 5246, https://doi.org/10.1039/C1EE02465E.
[79] H. Wang, J. F. Li, C. W. Nan, M. Zhou, W. Liu, B. P. Zhang, T. Kita, *Appl. Phys. Lett.* **2006**, *88* (9), 092104,
[80] Z.-Y. Li, J. F. Li, W. Y. Zhao, Q. Tan, T. R. Wei, C. F. Wu, Z. B. Xing, *Appl. Phys. Lett.* **2014**, *104* (11), 113905,
[81] J. Q. Li, S. P. Li, Q. B. Wang, L. Wang, F. S. Liu, W. Q. Ao, *J. Electron. Mater.* **2011**, *40* (10), 2063,
[82] F. R. Sie, H. J. Liu, C. H. Kuo, C. S. Hwang, Y. W. Chou, C. H. Yeh, *Intermetallics* **2018**, *92*, 113,
[83] Z. Y. Li, J. F. Li, *Adv. Energy Mater.* **2014**, *4* (2), 1300937,
[84] J. Li, Q. Tan, J. F. Li, *J. Alloys Compd.* **2013**, *551*, 143, https://doi.org/10.1016/j.jallcom.2012.09.067.
[85] Q. Tan, J. F. Li, *J. Electron. Mater.* **2014**, *43* (6), 2435,
[86] W. S. Liu, B. P. Zhang, L. D. Zhao, J. F. Li, *Chem. Mater.* **2008**, *20* (24), 7526,
[87] T. R. Wei, C. F. Wu, W. Sun, Y. Pan, J. F. Li, *RSC Adv.* **2015**, *5* (53), 42848,
[88] T. R. Wei, H. Wang, Z. M. Gibbs, C. F. Wu, G. J. Snyder, J. F. Li, **2014**,
[89] X. Yan, G. Joshi, W. Liu, Y. Lan, H. Wang, S. Lee, J. Simonson, S. Poon, T. Tritt, G. Chen, *Nano Lett.* **2011**, *11* (2), 556,
[90] M. Zou, J. F. Li, P. Guo, T. Kita, *J. Phys. D: Appl. Phys.* **2010**, *43* (41), 415403,
[91] M. Zou, J. F. Li, B. Du, D. Liu, T. Kita, *J. Solid State Chem.* **2009**, *182* (11), 3138,
[92] H. Zhu, R. He, J. Mao, Q. Zhu, C. Li, J. Sun, W. Ren, Y. Wang, Z. Liu, Z. Tang, *Nat. Commun.* **2018**, *9* (1), 1,
[93] S. Chen, K. C. Lukas, W. Liu, C. P. Opeil, G. Chen, Z. Ren, *Adv. Energy Mater.* **2013**, *3* (9), 1210, https://doi.org/10.1002/aenm.201300336.
[94] H. Zhang, Y. Wang, K. Dahal, J. Mao, L. Huang, Q. Zhang, Z. Ren, *Acta Mater.* **2016**, *113*, 41,

[95] J. Dong, F. H. Sun, H. Tang, J. Pei, H. L. Zhuang, H. H. Hu, B. P. Zhang, Y. Pan, J. F. Li, *Energy Environ. Sci.* **2019**, *12* (4), 1396,

[96] Z. Liu, J. Sun, J. Mao, H. Zhu, W. Ren, J. Zhou, Z. Wang, D. J. Singh, J. Sui, C. W. Chu, *PNAS* **2018**, *115* (21), 5332,

[97] G. Joshi, H. Lee, Y. Lan, X. Wang, G. Zhu, D. Wang, R. W. Gould, D. C. Cuff, M. Y. Tang, M. S. Dresselhaus, *Nano Lett.* **2008**, *8* (12), 4670, https://doi.org/10.1021/nl8026795.

[98] A. A. Usenko, D. O. Moskovskikh, M. V. Gorshenkov, A. V. Korotitskiy, S. D. Kaloshkin, A. I. Voronin, V. V. Khovaylo, *Scr. Mater.* **2015**, *96*, 9, https://doi.org/10.1016/j.scriptamat.2014.10.001.

[99] R. Basu, S. Bhattacharya, R. Bhatt, M. Roy, S. Ahmad, A. Singh, M. Navaneethan, Y. Hayakawa, D. K. Aswal, S. K. Gupta, *J. Mater. Chem. A* **2014**, *2* (19), 6922, https://doi.org/10.1039/C3TA14259K.

[100] Y. Jin, X. Zhang, Y. Xiao, W. He, D. Wang, J. Li, S. Zheng, D. Ren, Y. Qiu, L. D. Zhao, *Scr. Mater.* **2020**, *183*, 22,

[101] W. Liu, H. S. Kim, S. Chen, Q. Jie, B. Lv, M. Yao, Z. Ren, C. P. Opeil, S. Wilson, C. W. Chu, *PNAS* **2015**, *112* (11), 3269, https://doi.org/10.1073/pnas.1424388112.

[102] M. Li, S. Islam, S. Dou, X. Wang, *J. Alloys Compd.* **2018**, *769*, 59,

[103] M. Li, D. L. Cortie, J. Liu, D. Yu, S. Islam, L. Zhao, D. R. G. Mitchell, R. A. Mole, M. B. Cortie, S. Dou, *Nano Energy* **2018**, *53*, 993, https://doi.org/10.1016/j.nanoen.2018.09.041.

[104] B. Gahtori, S. Bathula, K. Tyagi, M. Jayasimhadri, A. Srivastava, S. Singh, R. Budhani, A. Dhar, *Nano Energy* **2015**, *13*, 36, https://doi.org/10.1016/j.nanoen.2015.02.008.

[105] J. Lei, Z. Ma, D. Zhang, Y. Chen, C. Wang, X. Yang, Z. Cheng, Y. Wang, *J. Mater. Chem. A* **2019**, *7* (12), 7006,

[106] A. A. Olvera, N. A. Moroz, P. Sahoo, P. Ren, T. P. Bailey, A. A. Page, C. Uher, P. Poudeu, *Energy Environ. Sci.* **2017**, *10* (7), 1668,

[107] K. Tyagi, B. Gahtori, S. Bathula, M. Jayasimhadri, N. K. Singh, S. Sharma, D. Haranath, A. Srivastava, A. Dhar, *J. Phys. Chem. Solids* **2015**, *81*, 100,

[108] M. Li, D. L. Cortie, J. Liu, D. Yu, S. Islam, L. Zhao, D. R. Mitchell, R. A. Mole, M. B. Cortie, S. Dou, *Nano Energy* **2018**, *53*, 993, https://doi.org/10.1016/j.nanoen.2018.09.041.

[109] Y. X. Chen, Z. H. Ge, M. Yin, D. Feng, X. Q. Huang, W. Zhao, J. He, *Adv. Funct. Mater.* **2016**, *26* (37), 6836,

[110] P. Peng, Z. N. Gong, F. Liu, M. J. Huang, W. Ao, Y. Li, J. Li, *Intermetallics* **2016**, *75*, 72,

[111] F. S. Liu, Z. N. Gong, M. J. Huang, W. Q. Ao, Y. Li, J. Q. Li, *J. Alloys Compd.* **2016**, *688*, 521,

[112] K. Tyagi, B. Gahtori, S. Bathula, M. Jayasimhadri, N. K. Singh, S. Sharma, D. Haranath, A. K. Srivastava, A. Dhar, *J. Phys. Chem. Solids* **2015**, *81*, 100,

[113] Y. B. Zhu, B. P. Zhang, Y. Liu, *Phys. Chem. Chem. Phys.* **2017**, *19* (40), 27664,

[114] Y. K. Lee, Z. Luo, S. P. Cho, M. G. Kanatzidis, I. Chung, *Joule* **2019**, *3* (3), 719,

[115] M. R. Burton, S. Mehraban, D. Beynon, J. McGettrick, T. Watson, N. P. Lavery, M. J. Carnie, *Adv. Energy Mater.* **2019**, *9* (26), 1900201,

[116] D. Li, J. C. Li, X. Y. Qin, J. Zhang, C. J. Song, L. Wang, H. X. Xin, *J. Electron. Mater.* **2017**, *46* (1), 79,

[117] H. Liu, X. Zhang, S. Li, Z. Zhou, Y. Liu, J. Zhang, *J. Electron. Mater.* **2017**, *46* (5), 2629,

[118] B. Zhong, Y. Zhang, W. Li, Z. Chen, J. Cui, W. Li, Y. Xie, Q. Hao, Q. He, *Appl. Phys. Lett.* **2014**, *105* (12), 123902,

[119] Q. Wen, C. Chang, L. Pan, X. Li, T. Yang, H. Guo, Z. Wang, J. Zhang, F. Xu, Z. Zhang, *J. Mater. Chem. A* **2017**, *5* (26), 13392,

[120] J. L. Lan, C. Deng, W. Ma, G. K. Ren, Y. H. Lin, X. Yang, *J. Alloys Compd.* **2017**, *708*, 955,

[121] R. Santos, M. Nancarrow, S. X. Dou, S. A. Yamini, *Sci. Rep.* **2017**, *7* (1), 1,

[122] W. Fan, S. Chen, B. Zeng, Q. Zhang, Q. Meng, W. Wang, Z. A. Munir, *ACS Appl. Mater. Interfaces* **2017**, *9* (34), 28635,
